# *MiR-126* Regulates Properties of SOX9^+^ Liver Progenitor Cells during Liver Repair by Targeting *Hoxb6*

**DOI:** 10.1016/j.stemcr.2020.07.005

**Published:** 2020-08-06

**Authors:** Yi Yan, Rui Wang, Xiongji Hu, Shichao Wang, Liang Zhang, Chenjiao Hou, Lisheng Zhang

**Affiliations:** 1 College of Veterinary Medicine, Bio-medical Center, Huazhong Agricultural University, Wuhan, Hubei 430070, China

**Keywords:** *MiR-126*, liver stem cells, injury repair, proliferation, differentiation

## Abstract

Liver progenitor cells (LPCs) have a remarkable contribution to the hepatocytes and ductal cells when normal hepatocyte proliferation is severely impaired. As a biomarker for LPCs, Sry-box 9 (*Sox9*) plays critical roles in liver homeostasis and repair in response to injury. However, the regulation mechanism of *Sox9* in liver physiological and pathological state remains unknown. In this study, we found that *miR-126* positively regulated the expression of *Sox9*, the proliferation and differentiation of SOX9^+^ LPCs by suppressing the translation of *homeobox b6* (*Hoxb6*). As a transcription factor, HOXB6 directly binds to the promoter of *Sox9* to inhibit *Sox9* expression, resulting in the destruction of the properties of SOX9^+^ LPCs in CCl_4_-induced liver injury. These findings revealed the role of *miR-126* in regulating SOX9^+^ LPCs fate by targeting *Hoxb6* in liver injury repair. Our findings suggest the potential role of *miR-126* as a nucleic acid therapy drug target for liver failure.

## Highlights

• *miR-126* promotes *Sox9* expression and maintains SOX9^+^ LPCs in adult mouse livers
• HOXB6 suppresses properties of SOX9^+^ LPCs in chronic liver injury model
• HOXB6 negatively regulates *Sox9 trans*-activity
• *miR-126* regulates properties of SOX9^+^ LPCs by targeting *Hoxb6*

## Graphical Abstract

**Figure:**
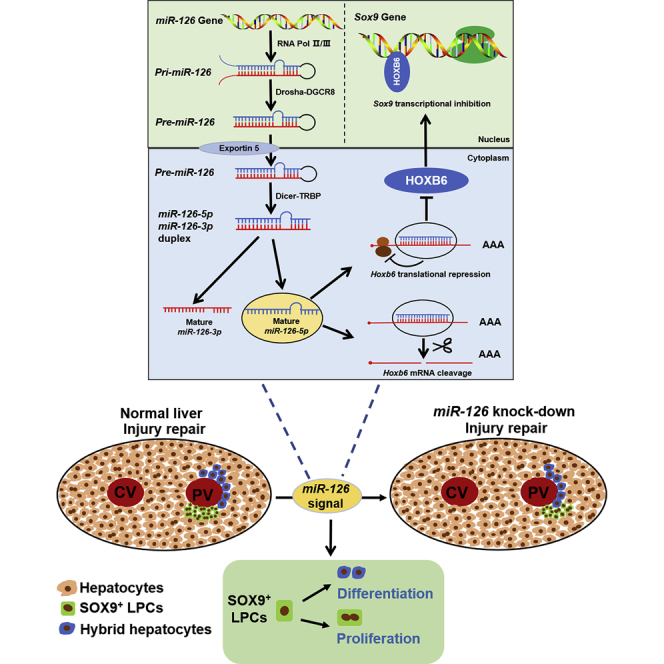

## Teaser

LPCs have a remarkable contribution to the physiological maintenance of liver homeostasis. However, the regulation mechanism of LPCs in liver physiology and pathology remains unknown. In this article, Zhang and his colleagues revealed the role of *miR-126* in regulating SOX9^+^ LPCs fate by targeting *Hoxb6* in liver injury repair. These findings suggest the potential role of *miR-126* in developing nucleic acid therapy drugs for liver failure.

## Introduction

*MicroRNAs (miRNAs)*, a class of endogenous, small non-coding RNAs composed of ∼22 nucleotides, bind to partial complementary sequences in the 3ʹ untranslated region (UTR) of their target transcripts and recruit RNA-induced silencing complex to inhibit the translation of these transcripts, they are involved in the regulation of cellular functions (Bartel, 2018; Mori et al., 2019). A recent study suggests that miRNAs, which are abundant in liver, can modulate a wide range of hepatocellular functions (Su et al., 2018). Our previous research showed that hepatic *miR-657* enhanced nuclear factor κB activity to promote hepatocellular carcinoma cell growth and transformation (Zhang et al., 2013). Moreover, *miR-*122 has been identified as a biomarker of acute liver failure in mice and humans (John et al., 2014; Luna et al., 2017). The newly reported *miR-221-3p* reduced secretion of CCR2 through post-transcription regulation of *Gnai2*, thus mitigating liver fibrosis (Tsay et al., 2019). The above-mentioned examples indicated that *miRNAs* play a critical role in liver physiological and pathological regulation. However, the specific roles of *miRNAs* in liver regeneration and repair, especially in regulating hepatic stem cell properties remain to be examined.

Our data showed that *miR-126* is encoded in the intron of *Egfl7* (Yan et al., 2020). Previous research reported that *miR-126* was highly expressed in normal hematopoietic stem cells (HSCs) and hematopoietic progenitor cells and restrained cell-cycle progression during hematopoiesis (Lechman et al., 2012). Recently, *miR-*126 has been reported to regulate the self-renewal of leukemia stem cells in chronic myelogenous leukemia (Zhang et al., 2018). Moreover, our previous research has shown that *miR-126* was involved in regulating liver aging. To be more specific, the knockdown of *miR-126* in bone marrow stromal cells (BMSCs) accelerated cell aging and inhibited hepatic repair functions of BMSCs (Yan et al., 2019). These results indicated that *miR-*126 might be involved in hepatic aging through liver stem cells (LSCs) or liver progenitor cells (LPCs). Although the roles of *miR-126* in stem cell function regulation and hepatic repair have been intensively studied, it remains largely unknown whether *miR-126* contributes to liver regeneration by regulating the LPC properties.

A few markers, including *Sox9*, *Axin2*, *Cd44*, and *Lgr5* were reported to be used to identify LSCs or LPCs (Huch et al., 2013; Wang et al., 2015). SOX9, a member of the sry-related high-mobility group box transcription factors, is closely related to cell proliferation and differentiation, and it regulates the stem cell homeostasis and differentiation (Ko et al., 2019; Mori-Akiyama et al., 2007). SOX9 is expressed throughout the biliary and pancreatic ductal epithelia (Alison and Lin, 2011). A previous study used *Sox9*-IRES-CreERT2 lineage tracing approach and found that the SOX9^+^ biliary compartment contributed to most new hepatocytes even during normal liver homeostasis. In addition, SOX9^+^ LPCs contributed to the formation of hepatocytes after liver injury (Furuyama et al., 2011; Tarlow et al., 2014). SOX9 was also weakly expressed in a population of periportal (PP) hepatocytes (named hybrid hepatocytes [HybHPs]) located in the portal triads of livers. HybHPs underwent extensive proliferation and replenished liver mass after chronic hepatocyte-depleting injury (Font-Burgada et al., 2015). The above-mentioned studies have demonstrated that SOX9 plays a critical role in liver regeneration and repair. However, the regulatory mechanisms of *Sox9* involved in liver regeneration and repair remain unclear.

In this study, we examined the effect of *miR-126* as regulatory factors on the properties of SOX9^+^ LPCs. Moreover, we revealed the regulatory mechanism by which HOXB6 was involved in *miR-126*-mediated liver injury repair. Our data demonstrated that the interaction between *miR-126-5p* and *Hoxb6* mRNA induced post-transcriptional silencing, leading to the stable expression of *Sox9*, thereby maintaining the stem cell properties of SOX9^+^ LPCs during the liver repair.

## Results

### *MiR-126* Promotes SOX9 Expression and Induces SOX9^+^ LPCs

**Figure 1:**
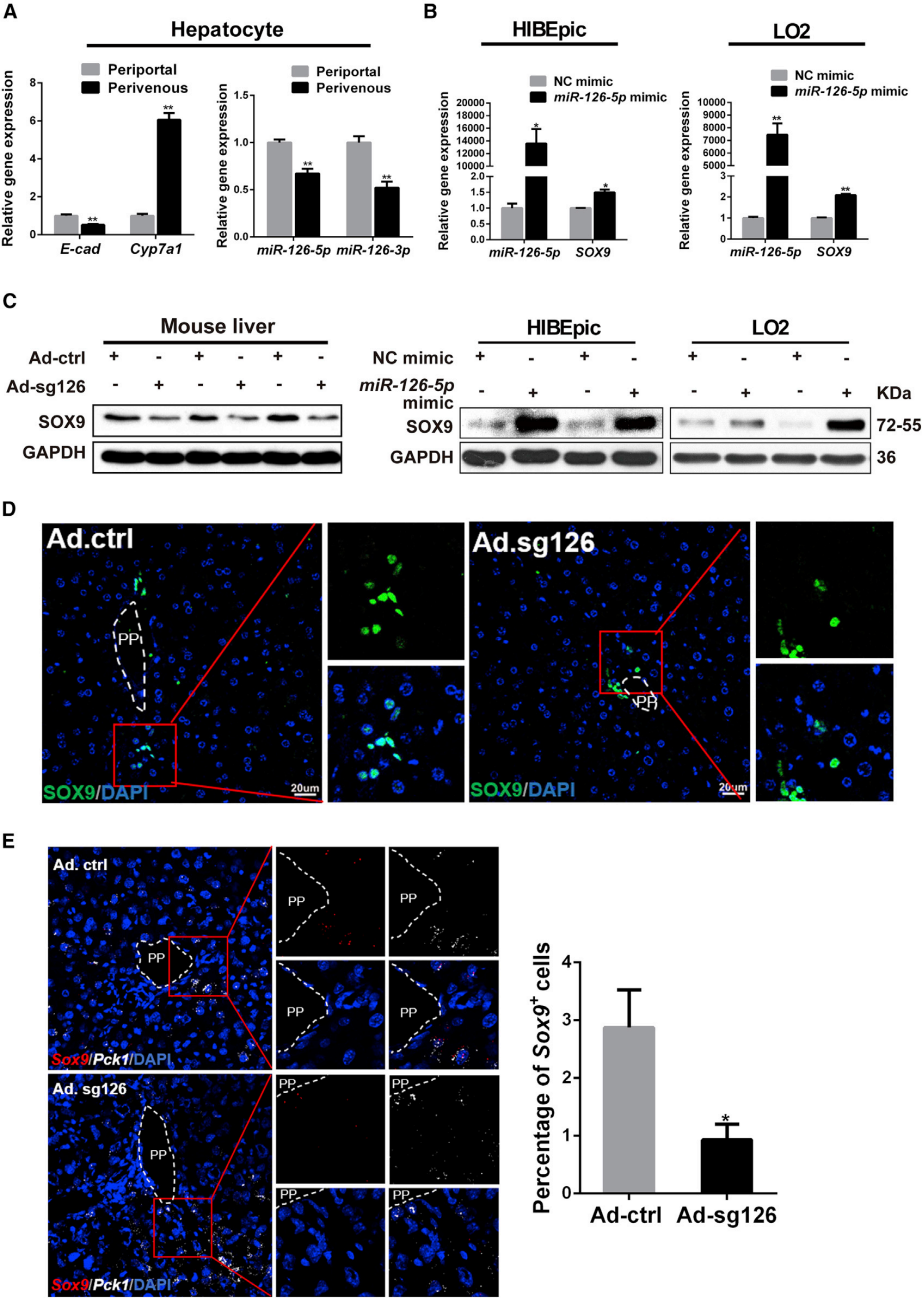
*miR-126* Regulates Liver Progenitor Cell Marker *Sox9* Expression and SOX9^+^ Hepatocyte-like Cell Number (A) Periportal (PP) hepatocytes and perivenous (PV) hepatocytes in 8-week-old mouse were isolated by using the digitonin/collagenase perfusion technique. Quantification of mature *miR-126-5p* and *miR-126-3p*, PP marker (*E-cad*), and PV marker (*Cyp7a1*) expressions in primary cultured PP hepatocytes and PV hepatocytes. (B) HIBEpic and LO2 cells were transfected with *miR-126-5p* mimics or negative control (NC) mimics for 48 h. Quantification of *miR-126-5p* and *SOX9* expressions in NC group and *miR-126-5p* group. The protein level of SOX9 was detected by western blotting. (C) Expression of SOX9 was measured at the protein level in Ad.ctrl and Ad.sg126 mouse livers. (D) Immunofluorescence was performed on the sections from paraffin-embedded tissue samples of Ad.ctrl mouse livers and Ad.sg126 mouse livers. Immunofluorescence of SOX9 was performed. DAPI (blue) shows nuclei. Scale bar represents 20 μm. (E) Representative images from RNAscope assays for *Sox9* mRNA levels and quantification of *Sox9*^+^ cells in livers of Ad.ctrl and Ad.sg126 mice. Red presents *Sox9*. White (*Pck1*) marks hepatocytes in the PP zone. Scale bar represents 20 μm. Data are expressed as means ± SD, n = 3 independent experiments containing three replicates (A and B), n = 6 mice per group containing three replicates (C–E). Significant difference is presented at the levels of ^∗^p < 0.05 and ^∗∗^p < 0.01 by two-tailed Student's t test. See also Figure S1.

Recent investigation has revealed that *miRNAs* are involved in liver regeneration and might serve as the therapeutic approach to liver fibrosis (John et al., 2014; Tsay et al., 2019). Our previous research has shown that *miR-126* contributes to hepatic anti-aging and damage repair (Yan et al., 2019). To reveal the effect of *miR-126* on anti-aging, we first detected the expression of stem cell-associated genes in C3H10 cells from which the *miR-*126 has been deleted. The result showed that there were no significant changes in the expression levels of *Axin2*, *Cd44*, and *Lgr5*, but *Sox9* abundance was significantly reduced, compared with that of control C3H10 cells (Figure S1A). Then, we measured the expression of the previously reported hepatic PP (*E-cad*) and perivenous (PV) (*Cyp7a1*) zonation genes in the purified hepatocytes separated from mouse livers (Han et al., 2019; Pu et al., 2016; Rocha et al., 2015). Expression levels of *miR-126-5p* and *miR-126-3p* genes were significantly higher in the PP hepatocytes than in the PV hepatocytes (Figures 1A and S1B). Previous studies reported that there were much more SOX9-expressing hepatocytes detected in the PP area than in the PV area (Font-Burgada et al., 2015; Halpern et al., 2017). Based on these findings, we hypothesized that there might be a correlation between these two genes.

To confirm the hypothesis, *miR-126-5p* was overexpressed by transfecting the *miR-126-5p* mimics into HIBEpic and LO2 cells, and we found that SOX9 expression was dramatically increased at the mRNA and protein levels (Figures 1B and S1B). We also inhibited *miR-126* expression by infecting BMSCs with sg126-expressing lentivirus (sg126). The result showed that the expression of *Sox9* in BMSCs was significantly decreased after infection with sg126-expressing lentivirus (Figure S1C). To further investigate the effects of *miR-126* on SOX9 expression in the liver, we disrupted *miR-*126 by delivering sg126-expressing adenovirus (Ad.sg126) into mouse livers, and we found that the SOX9 level in the Ad.sg126 group was much lower than that in the control adenovirus (Ad.ctrl) group by western blot (Figure 1C). Interestingly, a drastic decrease in the number of SOX9^+^ hepatocyte-like cells was observed around the PP area in *miR-126*-deleted mouse liver (Figures 1D and 1E).

### *Hoxb6* Is a Target of *miR-126-5p*

**Figure 2:**
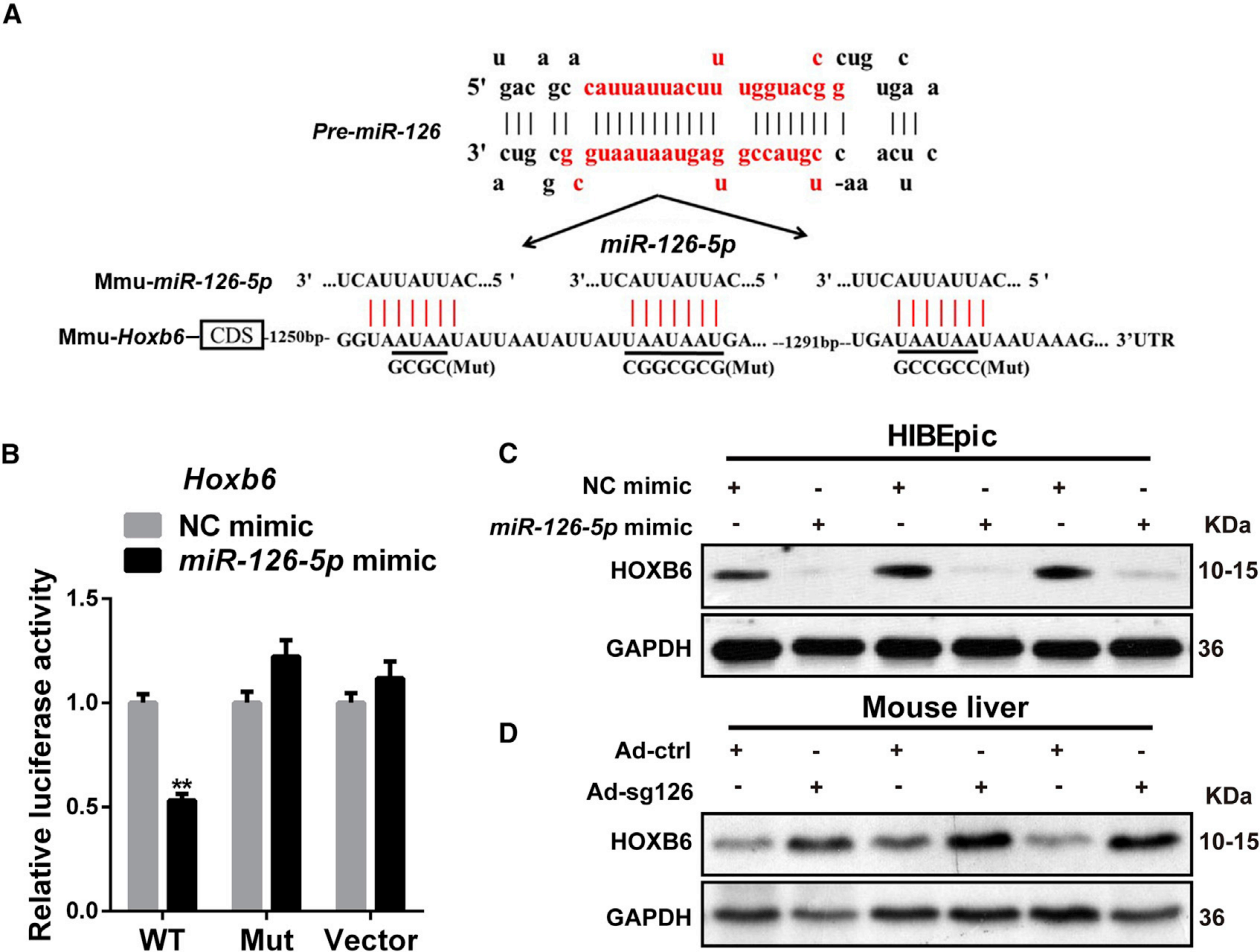
*Hoxb6* Is a Target of *miR-126-5p* (A) The 3′ UTR of *Hoxb6* gene contains binding sites for *miR-126-5p* according to TargetScan (http://www.targetscan.org/). Black underlined sequences indicate the point mutations used to generate mouse *Hoxb6* 3′ UTR Mut constructs. (B) Relative luciferase activity assays of luciferase reporter plasmid with mouse WT or Mut *Hoxb6* 3′ UTR, and that of vector were performed after co-transfection with *miR-126-5p* mimics or NC mimics in HeLa cells. *Hoxb6* 3′ UTR Mut seed complementary sites were shown in (A). (C) HOXB6 protein levels were measured by western blotting after transient transfection with *miR-126-5p* mimics or NC into HIBEpic cells. (D) Expression of HOXB6 was measured at the protein level in Ad.ctrl and Ad.sg126 mouse livers. Data are expressed as means ± SD, n = 3 independent experiments containing three replicates. Significant difference is presented at the level of ^∗∗^p < 0.01 by two-tailed Student's t test. See also Figure S2.

To identify putative targets of *miR-126-5p* contributing to the increase in SOX9^+^ LPCs in liver, we searched the candidate target genes by TargetScan and miRbase. Among various potential targets, we focused on *Hoxb6* gene since SOX9 was continuously expressed in *Hoxb6* mutant pancreata (Larsen et al., 2015), and *Hoxb6* regulates the generation, proliferation, or survival of erythroid progenitor cells in fetal livers, and disruption of the *Hoxb6* resulted in increased numbers of early erythrocyte progenitors (Kappen, 2000). So, we hypothesized that *miR-126* induces SOX9^+^ progenitors through *Hoxb6*. The 3′ UTR of the *Hoxb6* gene contains binding sites for *miR-126-5p* (Figure 2A). To determine whether *Hoxb6* is a direct target of *miR-126-5p*, we constructed luciferase reporter vector in which the *Hoxb6* 3′ UTR is placed behind the luciferase gene. We found that *miR-126-5p* inhibited luciferase activity, whereas no inhibition was observed when the *miR-126-5p* target site was mutated (Figures 2A, 2B, and S2A). Consistently, miRNA mimics-mediated overexpression of *miR-126-5p* suppressed the expression of HOXB6 in HIBEpic cells and HepG2 cells (Figures 2C and S2B). However, antagomir-mediated inhibition of *miR-126-5p* promoted the expression of HOXB6 (Figure S2B). Furthermore, lentivirus-mediated deletion of *miR-126* resulted in the upregulation of HOXB6 protein level in BMSCs and C3H10 cells (Figure S2C).

Consistent with the *in vitro* data, western blot analysis of the liver samples showed that deletion of *miR-126* increased hepatic HOXB6 levels (Figure 2D). Taken together, these results demonstrated that *miR-126-5p* directly targeted *Hoxb6*.

### HOXB6 Negatively Regulates SOX9 Expression *In Vitro* and *In Vivo*

**Figure 3:**
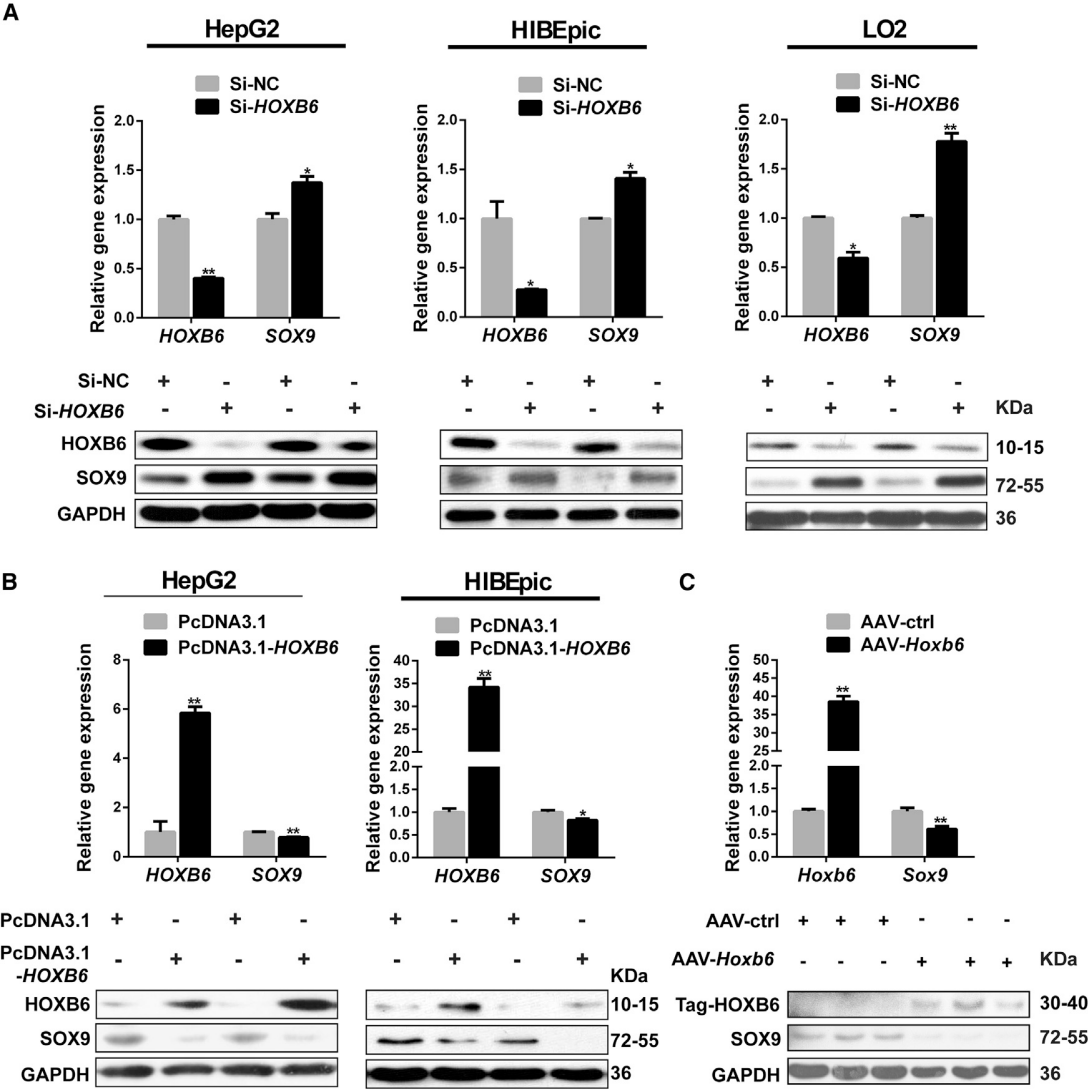
HOXB6 Negatively Regulates SOX9 Expression *In Vitro* and *In Vivo* (A) HepG2 cells, HIBEpic cells, and LO2 cells were transfected with the *HOXB6* siRNA (Si-*HOXB6*) or NC. At 48 h after transfection, western blotting and qRT-PCR analysis of HOXB6 and SOX9 expression in the NC group and the Si-*HOXB6* group were performed. (B) Western blotting and qRT-PCR analysis were conducted to detect HOXB6 and SOX9 expression in HepG2 and HIBEpic cells after HOXB6 exogenous overexpression. (C) Western blotting and qRT-PCR analysis of HOXB6 and SOX9 expression in AAV-ctrl and AAV-*Hoxb6* mice livers were performed. Data are expressed as means ± SD, n = 3 independent experiments containing three replicates (A and B), n = 6 mice per group containing three replicates (C). ^∗^p < 0.05 and ^∗∗^p < 0.01 by two-tailed Student's t test.

Based on the results described above, we explored whether HOXB6 regulated the expression of SOX9. *HOXB6* siRNA (Si-*HOXB6*) or PcDNA3.1-*HOXB6* was transfected into HepG2, HIBEpic, and LO2 cells to reduce or enhance HOXB6 function, respectively. Expression level of SOX9 was significantly increased in Si-*HOXB6*-transfected HepG2, HIBEpic, and LO2 cells (Figure 3A), whereas SOX9 was significantly reduced in PcDNA3.1-*HOXB6*-transfected HepG2 and HIBEpic cells (Figure 3B), indicating that HOXB6 suppressed SOX9 expression *in vitro*.

To confirm this observation, HOXB6 was overexpressed by tail vein injection of the adeno-associated virus (AAV)-*Hoxb6* or AAV control into mouse livers. We found that overexpression of HOXB6 significantly reduced *Sox9* mRNA abundance compared with the control group (Figure 3C). In line with the mRNA levels, SOX9 was even more profoundly downregulated at the protein level in the AAV-*Hoxb6* group (Figure 3C). Taken together, our data indicated that HOXB6 negatively regulated the expression of the LPC gene *Sox9*.

### HOXB6 Negatively Regulates *SOX9 trans*-Activity

**Figure 4:**
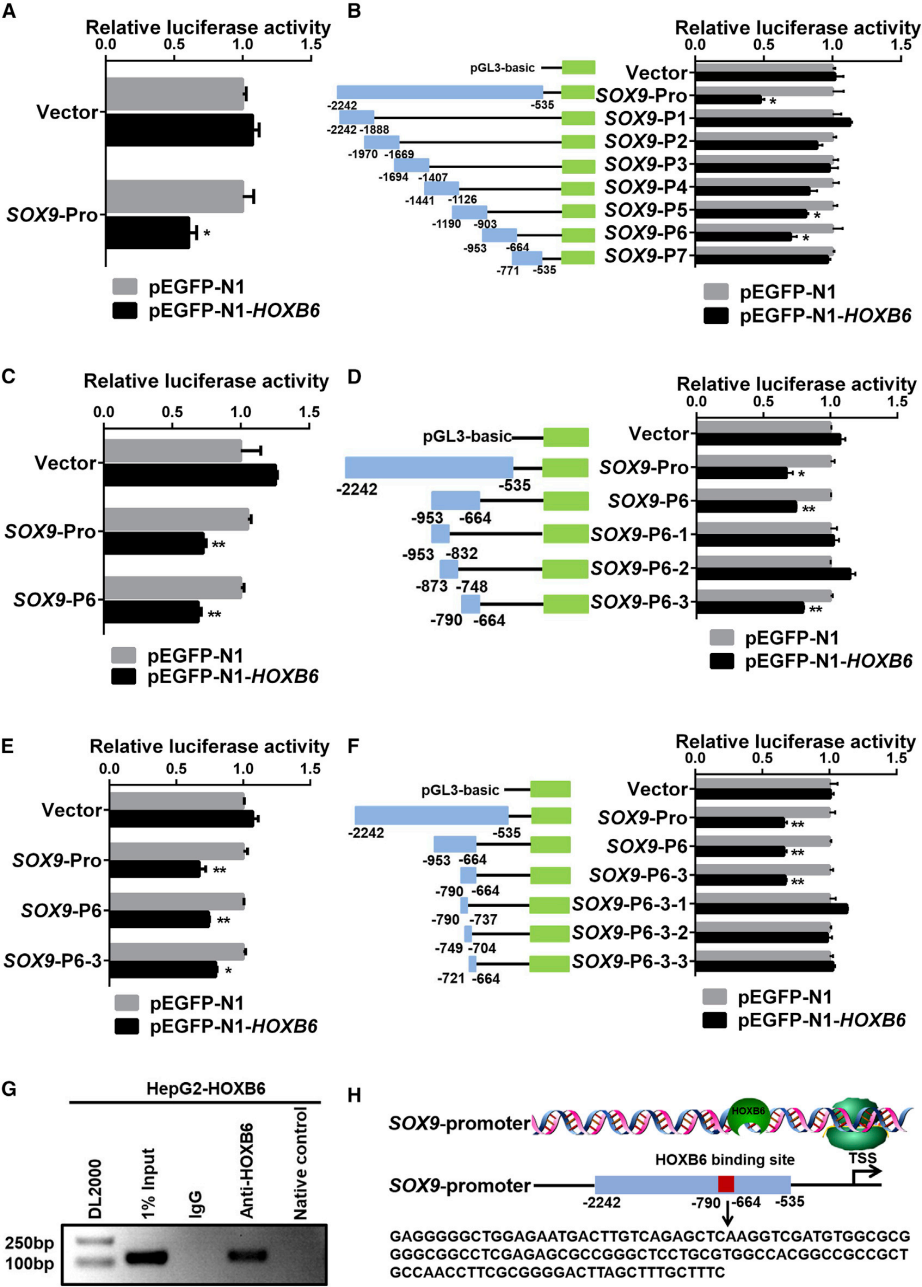
HOXB6 Negatively Regulates *SOX9 trans-*Activity (A) Relative luciferase activity analysis of the *SOX9* promoter-reporter constructs. pGL3-basic plasmid with a 1,700-base-pair fragment of the human *SOX9* promoter (position −2,242 to −535 relative to the transcription start site, *SOX9*-Pro) was transfected into HEK293T cells with the phRL-TK and pEGFP-N1 or pEGFP-N1-*HOXB6* eukaryotic expression plasmids. (B) Relative luciferase activity assays were performed in HEK293T cells by transiently co-transfecting *SOX9*-Pro (−2,242 to −535), *SOX9*-P1 (−2,242 to −1,888), *SOX9*-P2 (−1,970 to −1,669), *SOX9*-P3 (−1,694 to −1,407), *SOX9*-P4 (−1,441 to −1,126), *SOX9*-P5 (−1,190 to −903), *SOX9*-P6 (−953 to −664), *SOX9*-P7 (−771 to −535), and vector with expression plasmids of pEGFP-N1-*HOXB6* or pEGFP-N1. (C) Relative luciferase activity assays were performed in HEK293T cells by transiently co-transfecting *SOX9*-Pro (−2,242 to −535), *SOX9*-P6 (−953 to −664), and vector in a combination of expression plasmids of pEGFP-N1 or pEGFP-N1-*HOXB6*. (D) *SOX9*-P6 (−953 to −664) as well as various truncated fragments were inserted into pGL3-basic plasmid with luciferase reporter genes, including *SOX9*-P6-1 (−953 to 832), *SOX9*-P6-2 (−873 to −748), and *SOX9*-P6-3 (−790 to −664). Relative luciferase activity assays were performed in HEK293T cells by transiently transfecting the above plasmids with pEGFP-N1 or pEGFP-N1-*HOXB6*. (E) Relative luciferase activity assays were performed in HEK293T cells by transiently transfecting *SOX9*-Pro, *SOX9*-P6, *SOX9*-P6-3, and vector in a combination of expression plasmids of pEGFP-N1 or pEGFP-N1-*HOXB6*. (F) *SOX9*-P6-3 (−790 to −664) as well as various truncated fragments were inserted into the pGL3-basic plasmid with luciferase reporter gene, including *SOX9*-P6-3-1, *SOX9*-P6-3-2, and *SOX9*-P6-3-3. (G) Chromatin immunoprecipitation-PCR analysis. Chromatin was prepared and immunoprecipitated with specific antibodies against HOXB6 or IgG. The input DNA and DNA isolated from the precipitated chromatin were amplified by PCR using primers spanning the HOXB6 binding site, and the obtained PCR product was separated on a 1.5% agarose gel. Lanes: 1, marker; 2, input; 3, IgG; 4, HOXB6 antibody; 5, negative control. (H) Sequence of the *SOX9* promoter region. The listed nucleotide is the HOXB6 binding site. Data are expressed as means ± SD, n = 3 independent experiments containing three replicates. Significant difference is presented at the levels of ^∗^p < 0.05 and ^∗∗^p < 0.01 by two-tailed Student's t test. See also Figure S3.

*HOXB6* and other *HOX* genes have been thought to function as transcription factors, and they bind to DNA targets containing a TAAT sequence (Porcelli et al., 2019). Based on the description above, we hypothesized that HOXB6 could regulate *SOX9* transcription. We next attempted to identify the binding sites of HOXB6 by serially truncating regions within the *SOX9* promoter. At ∼3 kb immediately upstream of the *SOX9* transcriptional start site, we identified six potential TAAT-HOXB6 binding sites in the *SOX9* promoter region from −2,242 to −535 bp (Figure S3A). Next, we constructed a series of truncated promoter fragments aimed at removing these sites in a stepwise manner. As shown in Figures 4A and S3B, human or mouse *SOX9* promoter (*SOX9*-Pro), a full-length construct, exhibited a dramatic decrease in transcriptional activity by HOXB6 overexpression.

To determine the binding site in which the transcriptional activity of *SOX9* promoter was inhibited by HOXB6, seven ∼300-bp *SOX9* promoter-truncated fragments were constructed using *SOX9*-Pro as a template. These seven constructs were named *SOX9*-P1, *SOX9*-P2, *SOX9*-P3, *SOX9*-P4, *SOX9*-P5, *SOX9*-P6, and *SOX9*-P7 (Figure 4B). As shown in Figure 4B, luciferase activity of *SOX9*-P5 and *SOX9*-P6 were observed to be inhibited by HOXB6. But *SOX9*-P6 demonstrated a dramatically decreased transcriptional activity by HOXB6. Thus, these results revealed that the *SOX9*-P6 (−953 to −664 bp) region was responsible for *SOX9* transcriptional activity inhibition by HOXB6, and HOXB6 had the same effect on *SOX9*-P6 and *SOX9*-Pro (Figure 4C). Next, we constructed a series of *SOX9* promoter-truncated fragments with *SOX9*-P6 as a template. We named the three constructs *SOX9*-P6-1 (−953 to −832), *SOX9*-P6-2 (−873 to −748), and *SOX9*-P6-3 (−790 to −664) (Figure 4D). We detected the reduction in luciferase activity of the constructs containing only *SOX9*-P6-3 (−790 to −664) regions after overexpression of HOXB6 (Figures 4D and 4E). However, no reduction in luciferase activity was observed from three truncated fragments containing *SOX9*-P6-3-1 (−790 to −737), *SOX9*-P6-3-2 (−749 to −704), or *SOX9*-P6-3-3 (−721 to −664) promoter regions after overexpression of HOXB6 (Figure 4F).

To confirm the binding sites of HOXB6 to the *SOX9* promoter, we performed a chromatin immunoprecipitation (ChIP) assay. The crosslinked extracts were immunoprecipitated with antibodies against HOXB6 or control anti-IgG antibody. The crosslinked DNA was analyzed by using PCR with the primers designed to amplify the HOXB6-responsive region containing the *SOX9*-P6-3 (−790 to −664). HOXB6 was determined to be associated with the *SOX9* promoter region containing the SOX9-P6-3 fragments (Figure 4G). Taken together, our results suggested that *SOX9* was directly negatively regulated by HOXB6 with the binding sequences of *SOX9* promoter by HOXB6 shown in Figure 4H.

### AAV-Mediated Overexpression of HOXB6 Suppresses Proliferation and Differentiation of SOX9^+^ LPCs to Aggravate Damage in a CCl_4_ Chronic Liver Injury Model

**Figure 5:**
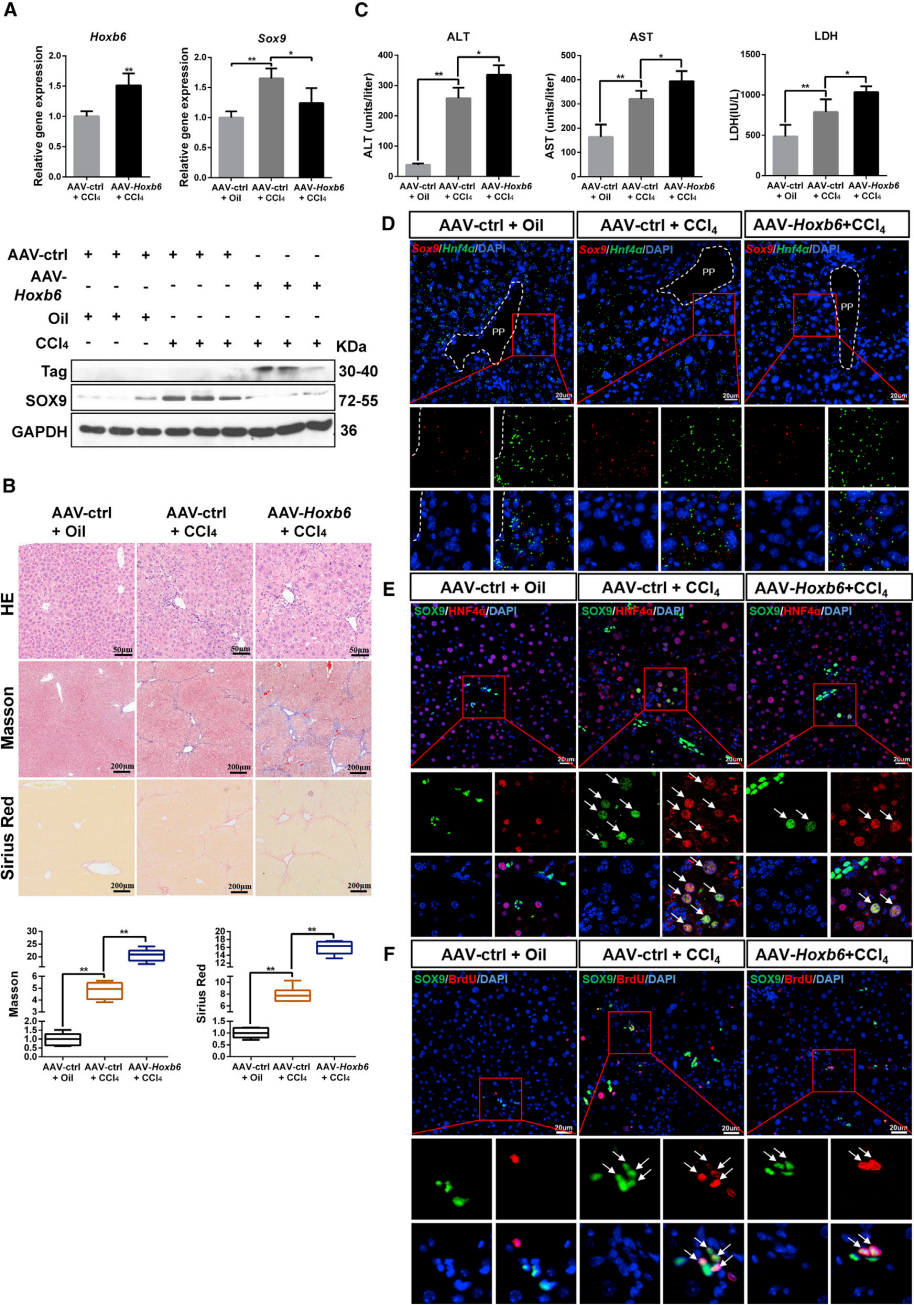
AAV-Mediated Overexpression of HOXB6 Suppresses the Proliferation and Differentiation of SOX9^+^ LPCs to Aggravate Damage in a CCl_4_ Chronic Liver Injury Model AAV-ctrl and AAV-*Hoxb6* were administered to 8-week-old C57 mice via tail vein injection. After 2 weeks, mice were treated with CCl_4_ (diluted in oil with 1:4) twice per week for 4 weeks, then livers and blood were collected. (A) Hepatic expression levels of SOX9 and HOXB6 were measured in AAV-ctrl, AAV-HOXB6 mice after CCl_4_ injury by qRT-PCR and western blot. (B) Representative H&E, Masson, and Sirius red staining in AAV-ctrl mice liver and AAV-*Hoxb6* mice liver after chronic hepatic injury by CCl_4_ treatment. Fibrosis was quantified by morphometric measurement of Masson and Sirius red. (C) Serum ALT, AST, and LDH levels of AAV-ctrl mice and AAV-HOXB6 mice after chronic hepatic injury by CCl4 treatment. (D) Representative images from RNAscope assays of *Hnf4α* and *Sox9* in livers of AAV-ctrl and AAV-*Hoxb6* mice after CCl_4_ injury. Red presents *Sox9*. Blue (DAPI) shows nuclei. Green (*Hnf4α*) marks hepatocytes. Scale bar represents 20 μm. (E) SOX9 and HNF4α double staining in periportal areas in the livers of AAV-ctrl and AAV-*Hoxb6* mice after CCl_4_ injury. Blue (DAPI) shows nuclei. Red (HNF4α) marks hepatocytes. Green presents SOX9. Scale bar represents 20 μm. (F) SOX9 and BrdU double staining in periportal areas in livers of AAV-ctrl and AAV-*Hoxb6* mice after CCl_4_ injury. Blue (DAPI) shows nuclei. Red (BrdU) marks the proliferating cells. Green presents SOX9. Scale bar represents 20 μm. Data are expressed as means ± SD, n = 6 mice per group containing three replicates. Significant difference is presented at the levels of ^∗^p < 0.05 and ^∗∗^p < 0.01 by two-tailed Student's t test. See also Figure S4.

Based on the above-mentioned regulation mechanism *in vitro*, an *in vivo* experiment was performed to reveal the mechanism by which HOXB6 regulates SOX9 in CCl_4_-induced liver injury model. To elucidate the regulation mechanism in liver, we first demonstrated the expression of HOXB6 and SOX9 in primary hepatocyte (Figure S4). Compared with control group, CCl_4_ treatment group exhibited a higher level of SOX9 expression (Figure 5A). After tail vein injection of 1 × 10^10^ particles of AAV-mediated overexpression of HOXB6 into the CCl_4_-treated mice, the expression of SOX9 was strongly suppressed at mRNA and protein levels (Figure 5A). Hematoxylin and eosin (H&E) staining results indicated that CCl_4_ treatment induced hepatotoxicity (Figure 5B). In addition, Masson and Sirius red staining results showed that CCl_4_ treatment promoted liver fibrosis (Figure 5B). At the same time, the levels of aspartate aminotransferase (ALT), alanine aminotransferase (AST), and lactic dehydrogenase (LDH) were observed significantly higher in the CCl_4_ group than in the control group (Figure 5C). Serological and histological analysis revealed that the treatment with AAV-*Hoxb6* resulted in a significantly increased liver damage and fibrosis as well as a significant increase in ALT, AST, and LDH levels (Figures 5B and 5C). Our results indicated that AAV-mediated overexpression of HOXB6 was able to obviously aggravate liver injury caused by CCl_4_.

To investigate the effect of HOXB6 on liver regeneration and injury repair through regulation of SOX9, we analyzed the behavior of SOX9^+^ cells during the regeneration period. Since SOX9^+^/HNF4α^+^ HybHPs was regenerated in the injured liver without developing into cancer (Font-Burgada et al., 2015), we focused on the *Hoxb6* effects on the HybHPs. *Hnf4α* and *Sox9* were co-stained to assess *Sox9*^+^ cell differentiation using an RNAscope assay. We found that the number of *Sox9*^+^/*Hnf4α*^+^ hepatocytes around the PP area in the CCl_4_ treatment group was larger than that in the control group. After AAV-*Hoxb6* treatment, CCl_4_-injured mice presented a smaller number of *Sox9*^+^/*Hnf4α*^+^ hepatocytes than the AAV control group (Figure 5D). The above results were also consistent with immunofluorescence results that HOXB6 decreased the number of SOX9^+^ hepatocytes (Figure 5E).

To investigate whether HOXB6 regulated SOX9^+^ LPC proliferation, we performed proliferation analysis in liver sections. Immunofluorescent double staining of the proliferation marker bromodeoxyuridine (BrdU) and SOX9 results showed an obvious increased proliferation of SOX9^+^ LPCs in the livers of the CCl_4_ injury group compared with that in control livers. After treatment with AAV-*Hoxb6*, a significant decrease in proliferation of SOX9^+^ LPCs was seen in the livers of the CCl_4_ injury group (Figure 5F). Collectively, these data demonstrated that overexpression of HOXB6 suppressed SOX9^+^ stem cell proliferation and propagation.

Taken together, our data indicated the downregulation of the LPC marker *Sox9* and the resultant decreased proliferation and differentiation of SOX9^+^ LPCs after HOXB6 exogenous expression in CCl_4_ chronic liver injury.

### Adenovirus-Mediated Deletion of *miR-126* Suppresses Proliferation and Differentiation of SOX9^+^ LPCs to Aggravate Damage in CCl_4_ a Chronic Liver Injury Model by Targeting *Hoxb6*

**Figure 6:**
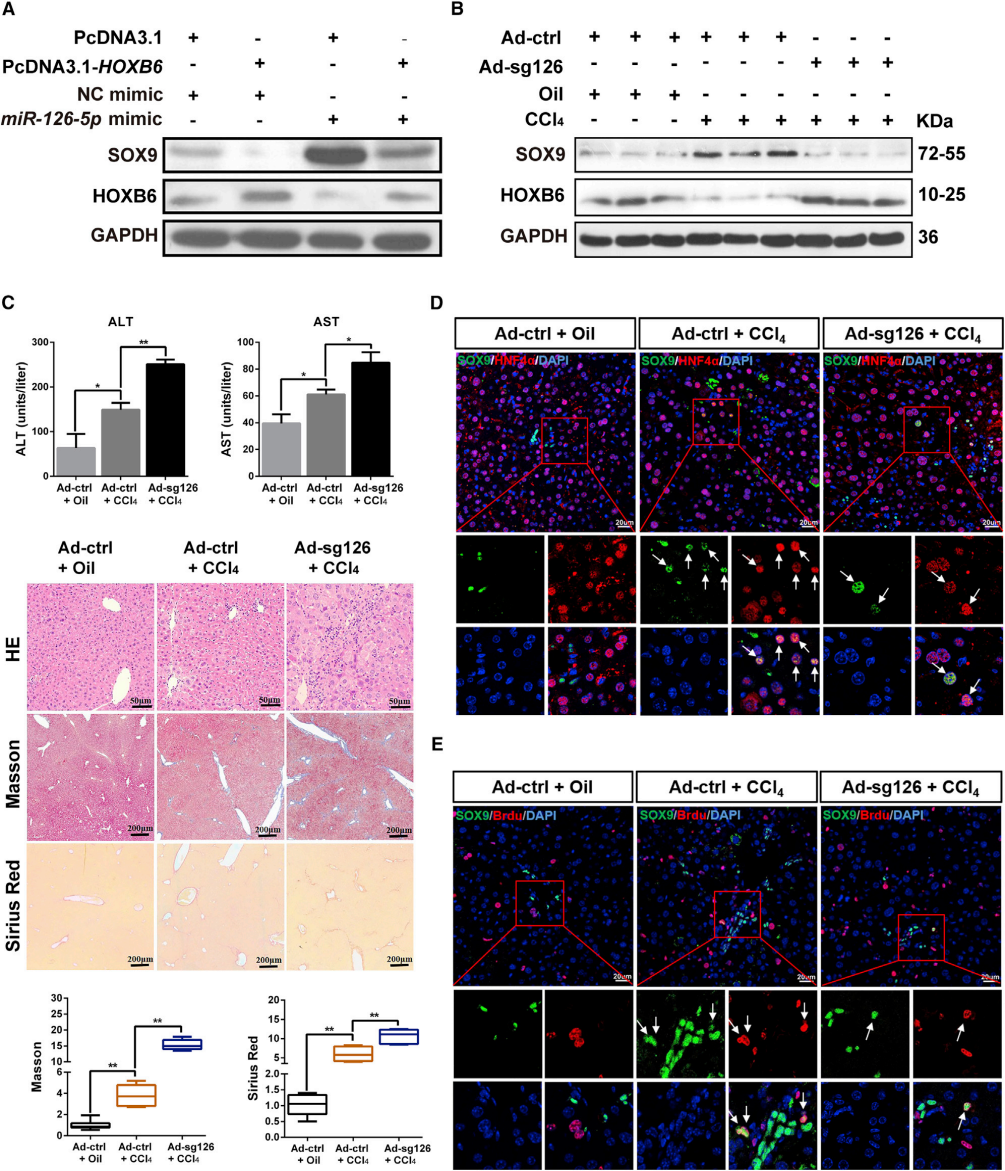
Adenovirus-Mediated Deletion of *miR-126* Suppresses the Proliferation and Differentiation of SOX9^+^ LPCs to Aggravate Liver Damage through *Hoxb6* in a CCl_4_ Chronic Injury Model (A) PcDNA3.1 or PcDNA3.1-*HOXB6* was co-transfected with *miR-126-5p* mimics or NC into HepG2 cells, respectively. HOXB6 and SOX9 levels were measured by western blotting. (B) Hepatic expression levels of SOX9 and HOXB6 were measured by western blot in CCl_4_-injured mice following CRISPR/Cas9-mediated *miR-126* gene disruption. (C) Hepatotoxicity and ALT/AST enzymes activities change in CCl_4_-injured mice following *miR-126* gene disruption. Representative H&E, Masson, and Sirius red staining in Ad-ctrl- and Ad-sg126-treated mice liver after chronic hepatic injury by CCl_4_. Fibrosis was quantified by morphometric measurement of Masson and Sirius red. (D) SOX9 and HNF4α double staining in periportal areas in livers of Ad-ctrl- or Ad-sg126-treated mice after CCl_4_ injury. Blue (DAPI) shows nuclei. Red (HNF4α) marks hepatocytes. Green presents SOX9. Scale bar represents 20 μm. (E) SOX9 and BrdU double staining in periportal areas in livers of Ad-ctrl- or Ad-sg126-treated mice after CCl_4_ injury. Blue (DAPI) shows nuclei. Red (BrdU) marks the proliferating cells. Green presents SOX9. Scale bar represents 20 μm. Data are expressed as means ± SD, n = 6 mice per group containing three replicates. Significant difference is presented at the levels of ^∗^p < 0.05 and ^∗∗^p < 0.01 by two-tailed Student's t test. See also Figure S5.

To further examine whether *miR-126* regulated *SOX9* by targeting *HOXB6*, HepG2 cells were transfected with negative control mimics, *miR-126-5p* mimics, PcDNA3.1, or PcDNA3.1-*HOXB6*, respectively. The qRT-PCR and western blot results revealed that HOXB6 overexpression abrogated the *miR-126-5p* mimics-induced promotion of SOX9 levels, suggesting that *miR-126-5p* regulated SOX9 by targeting HOXB6 (Figure 6A). The above results were consistent with in the cell line C3H10, from which the *miR-*126 has been deleted (Figure S5A).

We further studied *Sox9* regulation by *miR-126 in vivo* in a CCl_4_-induced chronic liver injury model. Mice were repeatedly administered a low dose of CCl_4_ to induce chronic liver injury. After tail vein injection of 1 × 10^10^ particles of Ad.ctrl or Ad.sg126 into the CCl_4_-treated mice, the expression of *miR-126* in liver was reduced (Figures S5B and S5C). To investigate whether *miR-126* contributed to liver injury repair, we collected the livers and blood for further analysis. Compared with the control group, the CCl_4_ treatment group exhibited a higher expression level of SOX9 and a lower level of HOXB6 (Figure 6B). After deletion of *miR-126* in the CCl_4_-treated mice, the drastically decreased expression of SOX9 and the increased expression of HOXB6 were observed (Figure 6B). We found that CCl_4_ treatment induced hepatotoxicity. Serological and histological analysis revealed that, after treatment with Ad.sg126, a significant increase in the severity of liver damage and fibrosis and in ALT and AST levels in the CCl_4_ injury group was observed (Figure 6C). In addition, the deletion of *miR-126* promoted a CCl_4_-induced increase in liver/body weight ratio (Figure S5D). These results indicated that deletion of *miR-126* could obviously aggravate liver injury by targeting *Hoxb6*.

To investigate whether *miR-126* affected liver regeneration and injury repair through *Sox9*, we analyzed the behavior of SOX9^+^ stem cells during the regeneration period after CCl_4_-induced liver injury. We found that the number of SOX9^+^/HNF4α^+^ hepatocytes around the PP area in the CCl_4_ administration group was larger than in the control group. After Ad.sg126 treatment, CCl_4_-injured mice presented a smaller number of SOX9^+^/HNF4α^+^ hepatocytes (Figure 6D). These results suggested that the deletion of *miR-126* suppressed the differentiation of SOX9^+^ LPCs into hepatocytes. Furthermore, immunofluorescent double staining of BrdU and SOX9 showed an obvious increase in SOX9^+^ LPC proliferation in the livers of the CCl_4_ injury group compared with control livers. After treatment with Ad.sg126, an obvious decrease in SOX9^+^ LPC proliferation in the livers of CCl_4_ injury group was observed (Figure 6E). The above results indicated that the deletion of *miR-126* suppressed SOX9^+^ LPC proliferation. Taken together, our data suggested that upregulation of the LPC marker *Sox9* and the resultant increased proliferation and differentiation of SOX9^+^ LPCs possibly explained why *miR-*126 might contribute to liver regeneration and repair.

## Discussion

The liver plays a pivotal role in the metabolism and detoxification of xenobiotics, which increases its possibility to be subjected to toxic damage, resulting in rapid loss of hepatic function, and high risk of losing regenerative and repair capacity (Font-Burgada et al., 2015). One previous study has shown that *miR-*302b and *miR-20a* repress hepatic functions by regulating transforming growth factor β (TGF-β) (Wei et al., 2013). Other previous studies have reported that *miRNAs* functioned by stimulating hepatocytes or by repressing cholangiocyte gene expression in mature hepatocytes (Gailhouste et al., 2013; Laudadio et al., 2012; Rogler et al., 2009). Our study found that *miR-126* was expressed in hepatocytes and biliary epithelial cells, and it was necessary for maintaining SOX9^+^ LSCs properties. Thus, the molecular mechanisms of *miR-126* regulation in LSCs are important for understanding liver repair.

LSCs or LPSs play important roles in the generation of hepatocytes and cholangiocytes (Suzuki et al., 2000). During persistent and severe liver damage, part of hepatocytes undergo dedifferentiation into LPCs for liver regeneration (Itoh and Miyajima, 2014). Although it has been well reported that the properties of LPCs were regulated by a number of intracellular and extracellular signaling pathways (Kitade et al., 2013; Parent and Beretta, 2008), the relationships among these signaling pathways and the contribution of *miR-126* in LPCs are largely unknown. To identify possible *miR-126* target genes involved in the regulation of LPCs properties, we performed a computational screen for genes with complementary sites of *miR-126* in their 3′ UTR by using open-access software. Here, we identified *Hoxb6* to be a *miR-126* target gene involved in regulating *Sox9* levels.

The *Hox* family of homeobox genes, as major transcriptional regulators in the body, encodes DNA binding proteins that play a crucial role in early body morphogenesis and hematopoietic development, and *Hox* family exhibits important effects on stem cell renewal, lineage commitment, and differentiation (Amsellem et al., 2003; Krumlauf, 1994; Larsen et al., 2015; Thorsteinsdottir et al., 1997). *Hoxb6*, as a member of the *Hox* family, was found to regulate HSC self-renewal, and its overexpression in mice resulted in HSC and myeloid progenitor cell expansion (Bhatlekar et al., 2018; Fischbach et al., 2005). However, HOXB6 functions in liver and the related molecular signals and mechanisms have not been investigated. The cell types that could respond to HOXB6 activation also remain unknown. The present study demonstrated that HOXB6 inhibited SOX9^+^ LPC proliferation and differentiation by reducing SOX9 levels in liver injury repair. Our results are also consistent with previous research findings that SOX9^+^ LPCs were a preexisting group around the PP area with the high regenerative and proliferative capability (Font-Burgada et al., 2015; Li et al., 2016), and the ability to differentiate into hepatocytes in livers (Ko et al., 2019). These studies support our results that upregulation of SOX9 promoted SOX9^+^ LPC proliferation and differentiation during liver fibrosis.

SOX9 has been reported to be regulated in a variety of cytokines and signaling pathways in different tissues. The deletion of *BMP type I receptor* gene in chondro-osteo progenitor cells led to chondrodysplasia and reduction in SOX9 (Yoon et al., 2005). In addition, SOX9 was induced by TGF-β in the kidney fibroblast, and it acted as an important downstream mediator of TGF-β signaling to promote renal fibrosis (Li et al., 2018). However, the regulation mechanism of *Sox9* transcription in the progression of liver injury repair remained unknown. Considering the principal role of HOXB6 in the HSC self-renewal and differentiation (Bhatlekar et al., 2018), and the fact that the HOXB6 protein repressed globin transcript levels in a DNA binding-dependent manner, thus repressing the erythroid phenotype in human leukemic cells (Shen et al., 2004), we investigated the effects of HOXB6 on SOX9 expression and properties of SOX9^+^ LPCs.

Although HOX homeodomain proteins were thought to function as transcription factors, most full-length HOX proteins, including HOXB6, bound only weakly to DNA targets containing a TAAT sequence, and the transcription of target genes was weakly activated or repressed (Catron et al., 1993; Graba et al., 1997; Sánchez-Higueras and Rastogi, 2019). In addition, in one previous study, transient reporter gene analysis results revealed that HOXB6 and other HOX proteins did not change the activity of luciferase reporter vectors containing synthetic TAAT multimers or putative gene regulatory regions (Shen et al., 2001). Similarly, in this study, our observations provided evidence that a binding region of HOXB6 was in the upstream of *SOX9* promoter, and that HOXB6 was responsible for the downregulation of SOX9 in hepatocytes and cholangiocytes. However, we found that HOXB6 did not change the luciferase activity in transient reporter gene assays using putative *SOX9* regulatory regions containing a TAAT sequence, but luciferase activity was found to be changed in *SOX9* promoter regions containing no TAAT sequences (*SOX9*-P5 and *SOX9*-P6). This result indicated that HOXB6 might exert transcriptional repression by binding to other specific sequences in the downstream target gene promoter region, which requires further study in the future. These findings indicated that HOXB6 affected the proliferation and differentiation of SOX9^+^ LPC by transcriptionally regulating the expression of SOX9. To our knowledge, this is the first attempt to report a LPC regulatory event during liver regeneration and repair.

Furthermore, the overall contribution of *miR-126* to the whole repair process, especially to liver injury repair, remains elusive. In this study, we found that, during liver injury repair, the expression of HOXB6 increases with deletion of *miR-126*, which leads to the inhibition of proliferation and differentiation in SOX9^+^ LPCs. In addition, we overexpressed *miR-126* or/and *HOXB6* in liver cancer cell lines, respectively, and we found that overexpression of HOXB6 abrogated *miR-126*-induced increases in stem cell-related gene *SOX9* expression. These results suggest that *miR-126-5p* can restrict the inhibition effect of HOXB6 on SOX9^+^ LPCs properties, thus contributing to hepatic repair and regeneration.

The *miR-*126 has broad biological and physiological implications. Thus, it is vital to fully understand the biological and physiological properties and functions of *miR-126*. In conclusion, we have identified *miR-126* as a regulator of stem cell-related gene *Sox9* expression and SOX9^+^ LPCs properties in liver repair by targeting *Hoxb6*. Our principal findings may have a clinical implication for treating liver diseases. In future studies, the molecular mechanisms of regulating the expression and function of *miR-126* in hepatocytes and cancer cells require further clarification and analysis. Knowledge of the molecular properties of liver injury repair and carcinogenesis will contribute to the development of anticarcinogenic agents.

## Experimental Procedures

Detailed methods are provided in the Supplemental Information.

### Mice and Injury Regimens

Adult C57BL/6J male mice were given standard rodent chow and water *ad libitum* under a standard 12-h light/dark cycle. For CCl_4_ injury experiments, CCl_4_ was injected into mice intraperitoneally at the dose of 2 mL/kg body weight, twice per week for 4 weeks (Tu et al., 2015). BrdU (Sigma-Aldrich, St. Louis, MO) was injected at the dose of 50 mg/kg 2 h before sacrifice (Zhang et al., 2015). Tissue and serum were collected at the end of the experiments. All procedures followed the Huazhong Agricultural University Guidelines for the Care and Use of Laboratory Animals.

### Cell Culture and Transient Transfection

The cell lines used in this study included HepG2, HIBEpic, LO2, BMSC, HeLa, and C3H10 cells. All cells were seeded into 6- or 24-well plates, and grown in high glucose DMEM (HyClone, Logan, UT) supplied with 10% (v/v) fetal bovine serum (Gibco BRL, Grand Island, NY) and 1% (v/v) penicillin-streptomycin. The following day, cells were transfected with plasmid, mimics, or siRNA. Transient transfection was performed using lipofectamine 2000 or lipofectamine RNAiMAX (Invitrogen, Carlsbad, CA). The primer sequences are listed in Table S1.

### Immunofluorescent Analysis

Liver tissues were immobilized with 4% paraformaldehyde (PFA), dehydrated, embedded in paraffin, sectioned at 5 μm, and processed for immunofluorescent staining. SOX9 antibody (AB5535, Millipore, USA) and HNF4α antibody (Ab41898, Abcam, Cambridge, England) were used to incubate sections at 4°C overnight. Slides were washed with PBS and incubated with corresponding secondary antibodies for 1 h, followed by PBS washes. The incubated slides were added with DAPI for nuclear staining and mounting. Images were acquired with a laser scanning confocal microscope (LSM710, Carl Zeiss Microscopy), were analyzed by Zen software with fixed parameters.

### Liver Histology and Immunohistochemical Staining

Liver tissues were immobilized with 4% PFA, dehydrated, embedded in paraffin, sectioned at 5 μm, and processed for H&E, Masson trichrome, and Sirius red staining.

### *In Situ* mRNA Hybridization

*In situ* detection of *Sox9* and *Hnf4α* RNA transcripts was carried out on OCT-embedded tissue sections using the RNAscope Multiplex Fluorescent Reagent Kit v.2 (Advanced Cell Diagnostics). RNAscope probes for *Sox9*, *Hnf4α*, standard negative probe for 4-hydroxy-tetrahydrodipicolinate reductase, and positive probe for peptidylprolyl isomerase B were used for *in situ* detection. The probe information for RNAscope assay is listed in Table S2.

### RNA Isolation and qRT-PCR

The RNAiso Plus (Takara, Japan) was used to isolate total RNA, including low-molecular-weight RNA from frozen samples and cell lines, according to the manufacturer's protocol. Then the first-strand cDNA was synthesized using the PrimeScript RT Reagent Kit with gDNA Eraser (Takara). Real-time PCR was performed using the MonAmp SYBR Green qPCR Mix (Low ROX). The relative levels were calculated using the comparative-Ct method (2^−ΔΔCt^ method). The primer sequences are listed in Table S3.

### Western Blots

For whole-cell protein extraction, liver tissues were prepared in lysis buffer (Beyotime, Jiangsu, China) according to the manufacturer's instructions. Protein lysates were separated by SDS-PAGE. Next, the gel was transferred to polyvinylidene difluoride membranes (Millipore). After being blocked with 5% skimmed milk, the membranes were incubated overnight with the anti-SOX9 (AB5535, Millipore), anti-HOXB6 (sc-166950X, Santa Cruz Biotechnology), anti-GAPDH (60004-I-Ig, Proteintech, Chicago), or Tag-3^∗^Flag-antibody (66008-3-Ig, Proteintech) at 4°C. Then, the membranes were incubated with the corresponding horseradish peroxidase-conjugated secondary antibodies at room temperature for 1.5 h. Finally, the membranes were visualized with enhanced chemiluminescence (Bio-Rad, USA).

### Biochemical Evaluation

Plasma was collected from blood after centrifugation (at 3,000 rpm) for 10 min at 4°C. Plasma ALT, AST, and LDH were determined to evaluate liver injury using a Multiskan MK3 microplate reader (Thermo Electron Corporation, USA) and commercial kits (Nanjing Jiancheng Bioengineering Institute, China), according to the manufacturer's instructions.

### Lentivirus, Adenovirus, and AAV Plasmid Construction and Production

Two pairs of CRISPR guide RNAs (gRNAs) targeting *pre-miR-126* gene were initially screened in NIH3T3cells. The gRNA targeting sgRNA3 displayed ∼50% mutagenesis at the on-target site in *pre-miR-126*, as determined by PCR and a T7EN1 cleavage assay (Yan et al., 2019). After targeting efficiency was confirmed, the sgRNAs were constructed into lentivirus and adenovirus plasmids. To completely overexpress HOXB6 *in vivo*, the *Hoxb6*-CDS sequence was inserted into the multiple cloning site in the pHBAAV-CMV-*Hoxb6*-3flag-T2A-ZsGreen plasmid. The two recombinant AAV plasmids were transfected into HEK293 cells with pAAV-RC and pHelper by using lipofectamine 2000 transfection reagent (Invitrogen, Carlsbad, CA). AAV-*Hoxb6* and AAV-ctrl (Hanbio, Shanghai, China) were packaged in AAV-293 cells.

### Intravenous Virus Injection for Liver Transduction

To intravenously inject adenovirus or AAVs, mice were restrained in a rodent restrainer, their tails were dilated using a heat lamp or warm water and sterilized by 70% ethanol, and 100 μL of concentrated AAV (1 × 10^10^ particles per mouse) was injected into the tail vein of each mouse.

### ChIP

ChIP assays were performed using the ChIP Assay kit (Beyotime) according to the user manual. In brief, HepG2 cells were transfected with pEGFP-N1-*HOXB6* for 24 h, and then incubated with formaldehyde at a final concentration of 1% (v/v) for 10 min at 37°C to crosslink the nuclear proteins to DNA. Subsequently, cells were sonicated and then immunoprecipitated with the antibody against HOXB6 (sc-166950, Santa Cruz Biotechnology), taking IgG as a negative control, or without anti-HOXB6 and IgG in the reaction as mock control. The captured chromatin was eluted and un-crosslinked, and the DNA was recovered. The ChIP-isolated DNA was subjected to PCR amplification using the primer pair spanning the *SOX9*-P6-3 in the *SOX9* promoter region. The primer sequences are listed in Table S4.

### Statistical Methods

Data are expressed as the means ± SD and were analyzed using Prism 6 (GraphPad). Statistical details of the experiments can be found in the Results and figure legends. Student's two-tailed t test (unpaired) was used to determine statistical significance differences between groups. Statistical significance was presented at the level of ^∗^p < 0.05, ^∗∗^p < 0.01, ^∗∗∗^p < 0.001.

### Data and Code Availability

The sequences of all primers are included in the paper.

## Authors Contributions

Y.Y. and Lisheng Zhang conceived and designed the study. Y.Y. provided the experimental data. Y.Y. and R.W. performed the cell and animal experiments. X.H., S.W., Liang Zhang, and C.H. provided assistance in molecular assays. Y.Y. and R.W. discussed and drafted the manuscript. Lisheng Zhang organized the data and wrote the manuscript.

## Supplemental Information

Document S1. Supplemental Experimental Procedures, Figures S1–S5, and Tables S1–S4

Document S2. Article plus Supplemental Information

## Footnotes

Supplemental Information can be found online at https://doi.org/10.1016/j.stemcr.2020.07.005.
