# Supplementary material for: MiR-126 Regulates Properties of SOX9+ Liver Progenitor Cells during Liver Repair by Targeting Hoxb6
Source: Stem Cell Reports. 2020 Aug 6;15(3):706–20. doi: 10.1016/j.stemcr.2020.07.005 (PMC7486193; doi:10.1016/j.stemcr.2020.07.005)
Supplement: Document S1. Supplemental Experimental Procedures, Figures S1–S5, and Tables S1–S4 [file mmc1.pdf]

Stem Cell Reports, Volume *15*

Supplemental Information

MiR-126 Regulates Properties of SOX9$^+$ Liver Progenitor Cells during Liver Repair by Targeting *Hoxb6*

Yi Yan, Rui Wang, Xiongji Hu, Shichao Wang, Liang Zhang, Chenjiao Hou, and Lisheng Zhang

Supplemental Information
This PDF file includes:

Figures S1 to S5
Tables S1 to S4
Supplemental Experimental Procedures
Supplemental References

Supplemental Figures

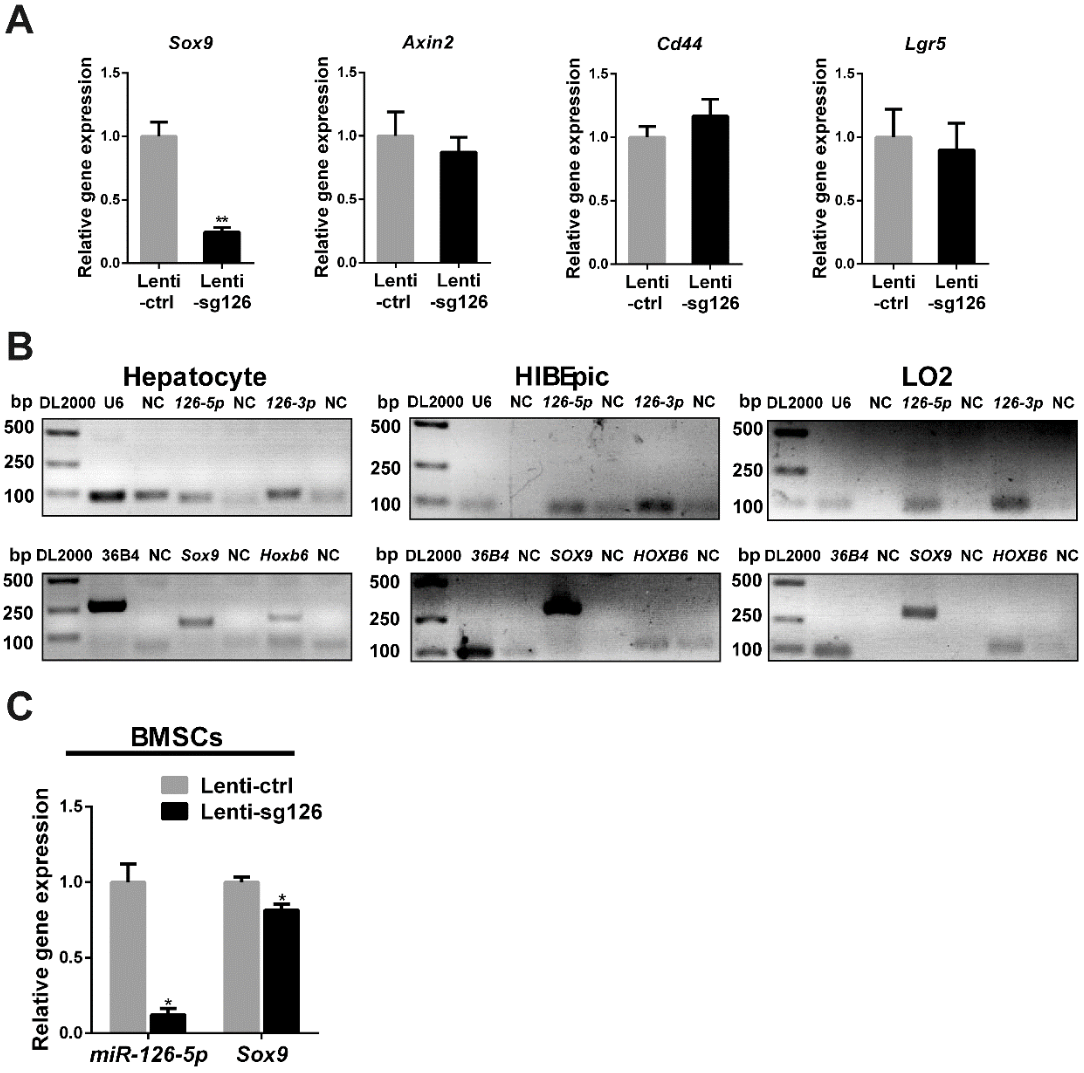

Figure S1. *miR-126* controls stem cell-specific gene *Sox9* expression *in vitro* and *in vivo*, related to Figure 1.

(A) C3H10 cells were infected with Lentivirus-control (Lenti-ctrl) or Lentivirus-sg126 (Lenti-sg126) to obtain stable cell lines. Quantification of *Sox9*, *Axin2*, *Cd44* and *Lgr5* expression in Lenti-ctrl C3H10 and Lenti-sg126 C3H10.

(B) PCR expression analysis of mature *miR-126-3p*, *miR-126-5p*, *HOXB6*, and *SOX9* in mouse primary hepatocytes (left), HIBEpic cells (middle) and LO2 (right).

(C) BMSCs were infected with Lentivirus-control (Lenti-ctrl) or Lentivirus-sg126 (Lenti-sg126) to obtain stable cell lines. Quantification of *miR-126-5p* and *Sox9* expression in Lenti-ctrl BMSCs and Lenti-sg126 BMSCs.

Data are expressed as means ± SD, n = 3 independent experiments containing three replicates. Significant difference is presented at the levels of $*p < 0.05$, $**p < 0.01$ by two-tailed Student's t-test.

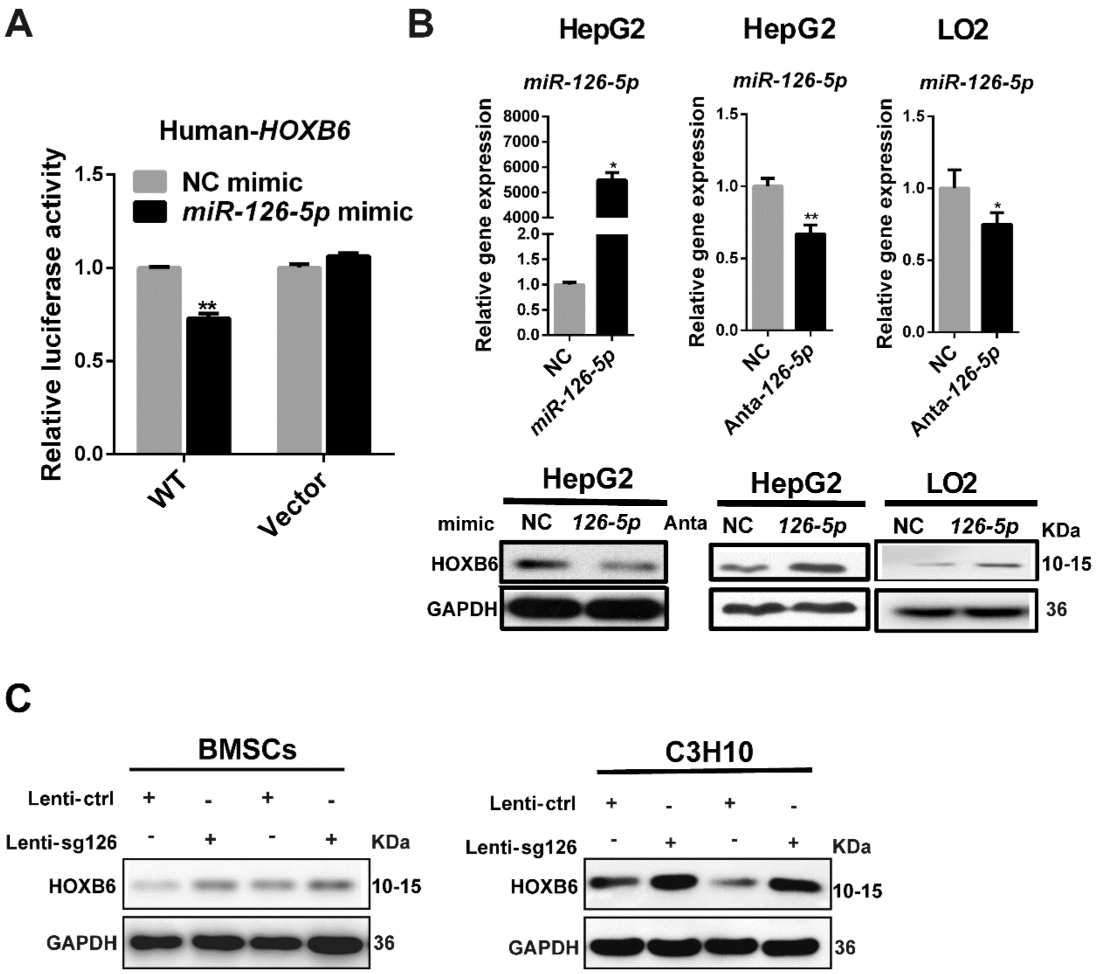

Figure S2. ***Hoxb6*** **is a target of** ***miR-126-5p*****, related to Figure 2.**

(A) Relative luciferase activity assays of luciferase reporters with human *HOXB6* 3ʹ UTR and psiCHECK2 were performed after co-transfection with *miR-126-5p* mimics or NC mimics in Hela cells.

(B) HepG2 cells or LO2 cells were transiently transfected with *miR-126-5p* mimics, antago*miR-126-5p* (Anta-126-5p) or stable negative control (NC). The mRNA levels of *miR-126-5p* were detected by qRT-PCR. The protein levels of HOXB6 were detected by western blot.

(C) BMSCs and C3H10 cells were infected with Lentivirus-control (Lenti-ctrl) or Lentivirus-sg126 (Lenti-sg126) to obtain stable cell lines. Western blotting analysis was performed to detect HOXB6 after deletion of *miR-126* in BMSCs and C3H10 cells.

Data are expressed as means ± SD, n = 3 independent experiments containing three replicates. Significant difference is presented at the levels of $*p < 0.05$, $**p < 0.01$ by two-tailed Student's t-test.

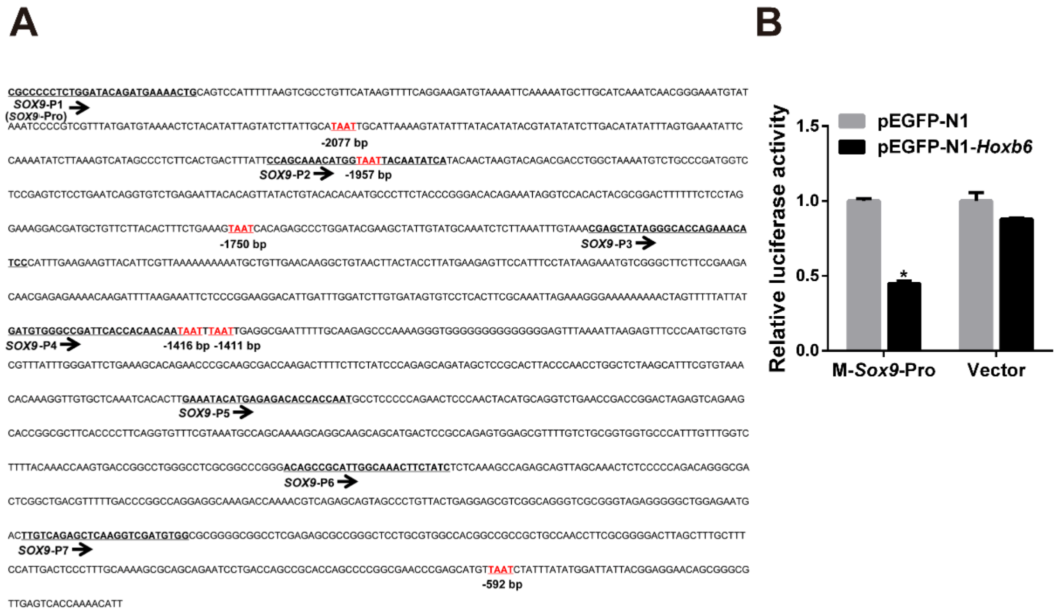

Figure S3. HOXB6 negatively regulates *SOX9* trans-activity, related to Figure 4.

(A) *SOX9*-Pro (1700bp) promoter sequence containing six putative HOXB6-binding sites (TAAT, red): I (-2077bp), II (-1957bp), III (-1750bp), IV (-1416bp), V (-1411bp), and VI (-592bp). The promoter truncated fragments were amplified by PCR. The primers were underlined.

(B) Relative luciferase activity analysis of the *Sox9* promoter–reporter constructs. A full-long fragment of the mouse *Sox9* promoter was co-transfected into HEK-293T cells with the phRL-TK and pEGFP-N1, or pEGFP-N1-*Hoxb6* eukaryotic expression plasmids. Forty-eight hours post transfection, the cell extracts were prepared and analyzed for luciferase activity.

Data are expressed as means ± SD, n = 3 independent experiments containing three replicates. Significant difference is presented at the levels of $*p < 0.05$ by two-tailed Student's t-test.

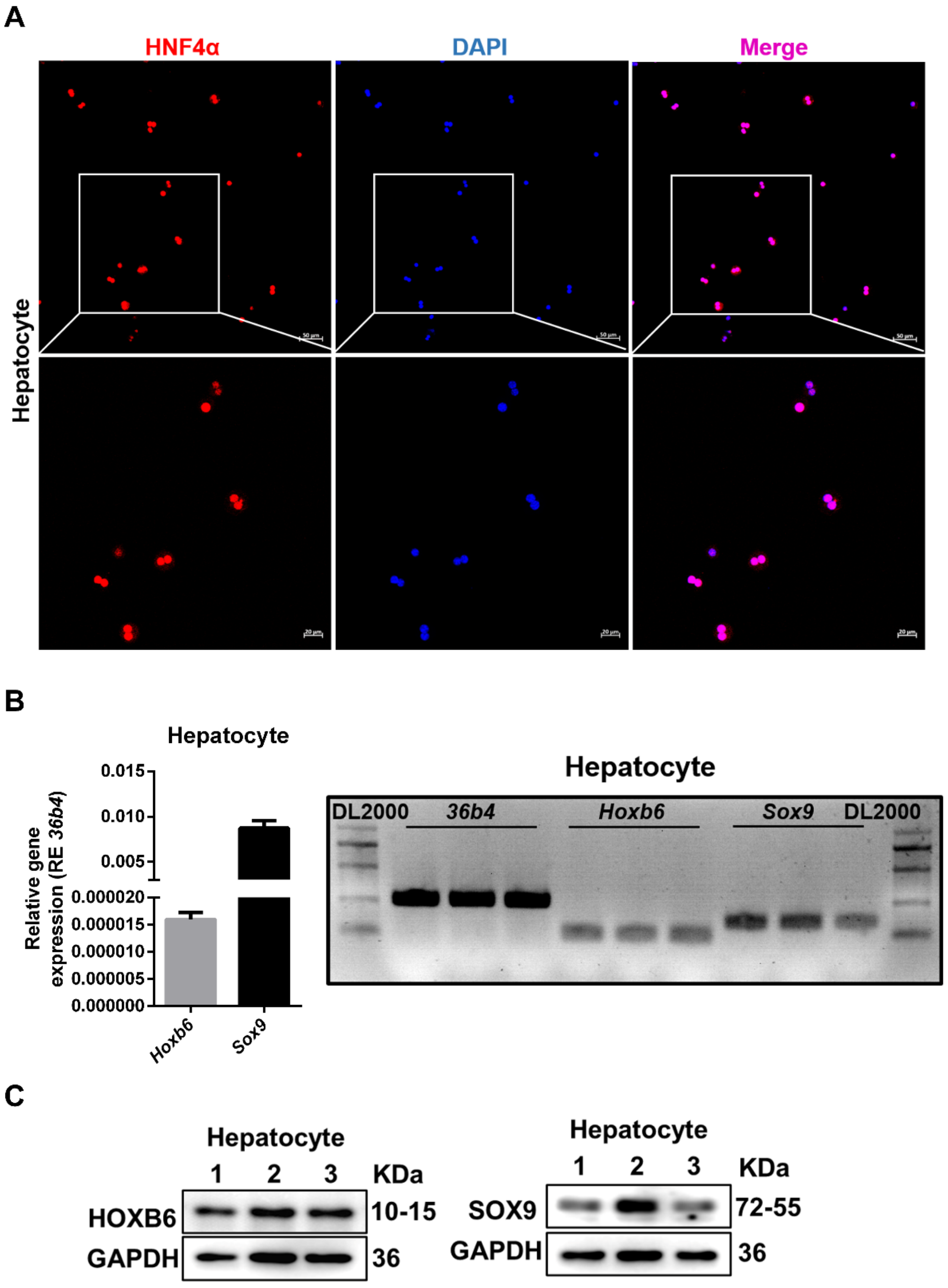

Figure S4. The expression of HOXB6 and SOX9 in primary hepatocyte, related to Figure 5.
Primary hepatocytes were isolated from normal fresh liver material obtained.
(A) Immunofluorescence micrographs of typical primary hepatocyte. hepatocyte sections labelled with antibodies against HNF4α. Blue (DAPI) shows nuclei. Red (HNF4α) marks hepatocytes. Scale bar represents 20μm.
(B) Normal mouse primary hepatocytes expression levels of *Hoxb6* and *Sox9* were measured by qRT-PCR and PCR.
(C) Normal mouse primary hepatocytes expression levels of HOXB6 and SOX9 were measured by western blot.
Data are expressed as means ± SD, n = 3 mice per group containing three replicates.

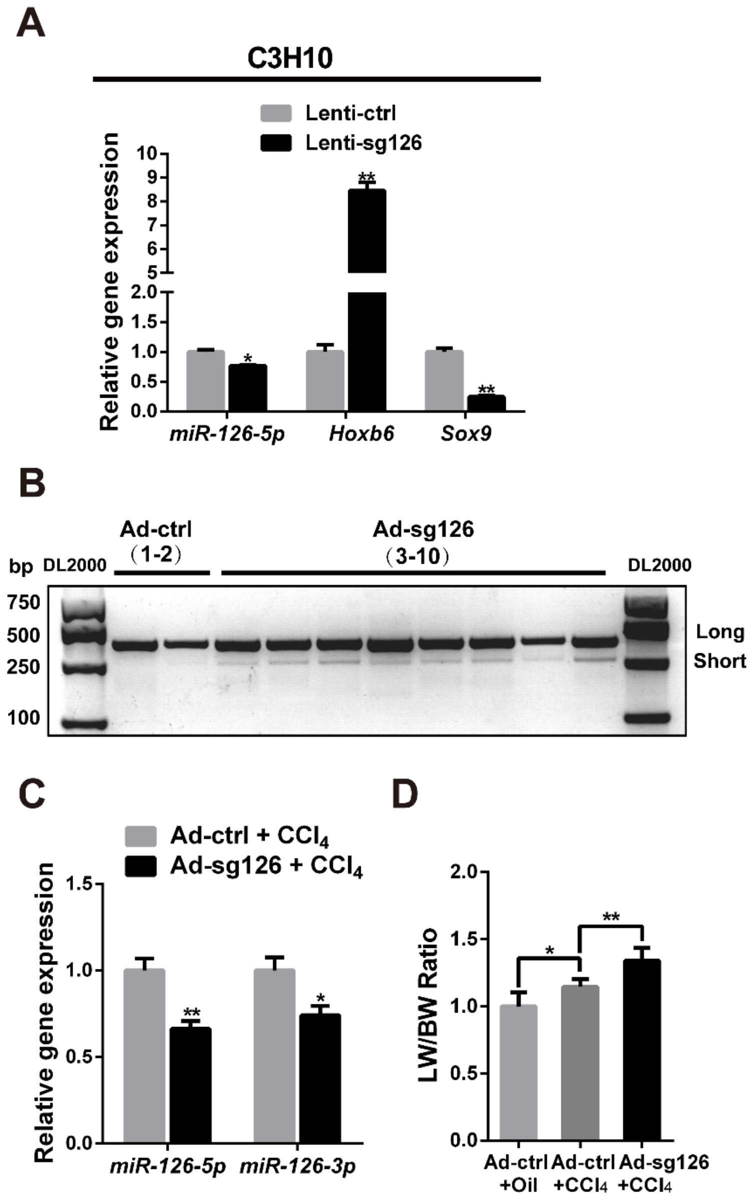

Figure S5. Deletion of *miR-126* suppresses functions of SOX9^{+} LPCs by targeting *Hoxb6*, related to Figure 6.

(A) C3H10 cells were infected with Lentivirus-control (Lenti-ctrl) or Lentivirus-sg126 (Lenti-sg126) to obtain stable cell lines. Quantification of *miR-126-5p*, *Hoxb6*, and *Sox9* expression in Lenti-ctrl C3H10 and Lenti-sg126 C3H10.

(B) PCR cleavage assays were performed with genomic DNA from liver samples from mice at day 7 post infection with Ad.ctrl (lanes 1–2) and Ad.sg126 (lanes 3–10).

(C) Hepatic expression levels of *miR-126-3p* and *miR-126-5p* genes were measured in Ad-ctrl-treated or Ad-sg126-treated mice after CCl_4 injury by qRT-PCR.

(D) Effect of *miR-126* deletion on the LW/BW ratio after CCl_4 injury.

Data are expressed as means ± SD, n = 3 independent experiments containing three replicates (A), n = 6 mice per group containing three replicates (C and D). Significant difference is presented at the levels of $*p < 0.05$, $**p < 0.01$ by two-tailed Student's t-test.

Supplemental Tables

Table S1. Oligonucleotide Sequences for *miR-126a* and *Hoxb6* siRNA in this study, Related to Figure 1, Figure 2, Figure 3, and Figure 6.

Gene name	Primer sequence (5’-3’)
miR-126-5p mimics sense	CAUUAUUACUUUUGGUACGCG
miR-126-5p mimics antisense	CGUACCAAAAGUAAUAAUGUU
M-*Hoxb6*-siRNA sense	CCACUGGAAGGAGCACAUATT
M-*Hoxb6*-siRNA antisense	UAUGUGCUCCUUCCAGUGGTT
Negative control sense	UUCUCCGAACGUGUCACGUTT
Negative control antisense	ACGUGACACGUUCGGAGAATT
miR-126a-5p-antagomir	CGCGUACCAAAAGUAAUAAUG

Table S2. Probe information for RNAscope® assay in this study, Related to Figure 1 and Figure 5.

Official symbol	Cat No.	Channel	Detection Kit
Sox9	401051	C3	RNAscope® Multiplex Fluorescent Reagent Kit v2
Hnf4α	497651	C2	
3-Plex Positive Control Probe-Mm	320881	C3	
3-Plex Negative Control Probe (*Dapb*)	320871	C3	

Table S3. Primer sequences for reverse transcription PCR and qRT-PCR in this study, Related to Figure 1, Figure 3, and Figure 5.

Gene name	Primer sequence (5’-3’)
RT-loop-*miR126-5p*	GTCGTATCCAGTGCAGGGTCCGAGGTATTCGCACTGGATAC GACCGCGTA
RT-loop-*miR126-3p*	GTCGTATCCAGTGCAGGGTCCGAGGTATTCGCACTGGATAC GACCGCATT
miR-126a-5p-F	GGCGACGGCATTATTACTTTTG
miR-126a-3p-F	AAGGTTGTTCGTACCGTGAGT
UR	CAGTGCAGGGTCCGAGGT
U6-F	GCTTCGGCAGCACATATACTAA
U6-R	CGAATTTGCGTGTCATCCTT
H-*GAPDH*-RT-F	CTCTGGTAAAGTGGATATTG
H-*GAPDH*-RT-R	GGTGGAATCATATTGGAAC
H-*SOX9*-RT-F	TCCTCAGGCTTTGCGATTT
H-*SOX9*-RT-R	TGCTCGGGCACTTATTGG
H-*HOXB6*-RT-F	CTACCGCGAGAAAGAGTCGG
H-*HOXB6*-RT-R	TGTTGCACGAATTCATCCGC
M-*36b4*-RT-F	TGGAGACAAGGTGGGAGCC
M-*36b4*-RT-R	CACAGACAATGCCAGGACGC
M-*Sox9*-RT-F	TGCAAGCTGGCAAAGTTGAT
M-*Sox9*-RT-R	TCAGTTCACCGATGTCCACG
M-*Hoxb6*-RT-F	CCTACACACGCTACCAGACC
M-*Hoxb6*-RT-R	TGAGACGCACTGAGCAGTTT
M-*Cyp7a1*-RT-F	GGGGATTGCTGTGGTAG
M-*Cyp7a1*-RT-R	CAGGGAGTTTGTGATGAAG
M-*E-cad*-RT-F	TGATGATGCCCCCAACACTC
M-*E-cad*-RT-R	CCAAGCCCTTGGCTGTTTTC

Table S4. Primer sequences for luciferase reporter assays and ChIP assays in this study, Related to Figure 2 and Figure 4.

Gene name	Primer sequence (5'-3')
m-*Hoxb6*-3UTR -F	GCGCTCGAGTGAGAATCGGACTCACTTGAT
m-*Hoxb6*-3UTR-R	GCGCGCGGCCGCTTGCGAGTTTTCACATCTTTA
m-*Hoxb6*-3UTR-mut1-F	GATTATGATGATGATGATAAGCCGCCTAAAGATGTGAAAACTCG
m-*Hoxb6*-3UTR-mut1-R	CGAGTTTTCACATCTTTAGGCGGCTTATCATCATCATCATAATC
m-*Hoxb6*-3UTR-mut2-F	GGTAGCGCTATTAATATTATCGGCGCGGATTATGATGATGATGATAAGCCGCCTAAAG
m-*Hoxb6*-3UTR-mut2-R	CCGCGCCGATAATATTAATAGCGCTACCGAAGTATTTCACGTCCGGAGCTAAGACAAG
H-*SOX9*-Pro-full-long-F	GGGGTACCCGCCCCCTCTGGATACAGATGAAAACTG
H-*SOX9*-Pro-full-long-R	CCCAAGCTTAATGTTTTGGTGACTCAACGCCCGCTG
H-*SOX9*-P1-F	GGGGTACCCGCCCCCTCTGGATACAGATGAAAACTG
H-*SOX9*-P1-R	CCCAAGCTTGAGACTCGGAGACCATCGGGCAGAC
H-*SOX9*-P2-F	GGGGTACCCCAGCAAACATGGTAATTACAATATCA
H-*SOX9*-P2-R	CCCAAGCTTGGATGTTTCTGGTGCCCTATAGC
H-*SOX9*-P3-F	GGGGTACCCGAGCTATAGGGCACCAGAAACATCC
H-*SOX9*-P3-R	CCCAAGCTTAATTAAATTATTGTTGTGGTGAATCGGCC
H-*SOX9*-P4-F	GGGGTACCGATGTGGGCCGATTCACCACAACAAT
H-*SOX9*-P4-R	CCCAAGCTTCGGTTCAGACCTGCATGTAGTTGGGAG
H-*SOX9*-P5-F	GGGGTACCGAAATACATGAGAGACACCACCAAT
H-*SOX9*-P5-R	CCCAAGCTTGTTTGCTAACTGCTCTGGCTTTG
H-*SOX9*-P6-F	GGGGTACCACAGCCGCATTGGCAAACTTCTATC
H-*SOX9*-P6-R	CCCAAGCTTGAAAGCAAAGCTAAGTCCCCGCG
H-*SOX9*-P7-F	GGGGTACCTTGTCAGAGCTCAAGGTCGATGTGG
H-*SOX9*-P7-R	CCCAAGCTTAATGTTTTGGTGACTCAACGCCC
H-*SOX9*-P6-1-F	GGGGTACCACAGCCGCATTGGCAAACTTCTATC
H-*SOX9*-P6-1-R	CCCAAGCTTGCTCTGACGTTTTGGTCTTTGCCT
H-*SOX9*-P6-2-F	GGGGTACCGTTTTTGACCCGGCCAGG
H-*SOX9*-P6-2-R	CCCAAGCTTCACATCGACCTTGAGCTCTGAC
H-*SOX9*-P6-3-F	GGGGTACCGAGGGGGCTGGAGAATGACTT
H-*SOX9*-P6-3-R	CCCAAGCTTGAAAGCAAAGCTAAGTCCCCG
H-*SOX9*-P6-3-1--F	GGGGTACCGAGGGGGCTGGAGAATGACTT
H-*SOX9*-P6-3-1--R	CCCAAGCTTCCGCCCCGCGCCA
H-*SOX9*-P6-3-2--F	GGGGTACCGGTCGATGTGGCGCGG
H-*SOX9*-P6-3-2--R	CCCAAGCTTCCGTGGCCACGCAGGAG
H-*SOX9*-P6-3-3--F	GGGGTACCGCTCCTGCGTGGCCACG
H-*SOX9*-P6-3-3--R	CCCAAGCTTGAAAGCAAAGCTAAGTCCCCG
H-*SOX9*-P6-3-ChIP-F	CTGGAGAATGACTTGTCAGAGC
H-*SOX9*-P6-3-ChIP-R	AAAGCTAAGTCCCCGCGAA

H: Human M: Mouse F: Forward R: Reverse

Supplemental Experimental Procedures

Mice and Injury Regimens

Adult C57BL/6J male mice (8 weeks old) were housed in a pathogen-free animal facility under a standard 12 hours light/dark cycle. Mice were given standard rodent chow and water ad libitum. For CCl_4 injury experiments, CCl_4 was diluted with paraffin oil to achieve a final concentration of 20%, and the diluted CCl_4 was injected into mice intraperitoneal at the dose of 2 ml/kg body weight, twice per week for four weeks. Control mice were provided with an equal volume of oil vehicle. Bromodeoxyuridine (BrdU) (Sigma-Aldrich, St. Louis, MO) was injected at the dose of 50 mg/kg 2 hours before sacrifice. After mice were killed, liver tissues were frozen in liquid nitrogen or fixed in 10% buffered formalin and embedded in paraffin and serum were collected. All procedures followed the Huazhong Agricultural University Guidelines for the Care and Use of Laboratory Animals.

Cell Culture and Transient Transfection

The cell lines used in this study included HepG2, HIBEpic, LO2, BMSC, HeLa, and C3H10. All cells were seeded into 6-well or 24-well plates, and grown in high glucose DMEM (Hyclone, Logan, UT, USA) supplied with 10% (vol/vol) fetal bovine serum (FBS; Gibco BRL, Grand Island, NY, USA) and 1% (vol/vol) penicillin-streptomycin. The following day, cells were transfected with plasmid, mimics, or siRNA. Transient transfection was performed using Lipofectamine 2000 or Lipofectamine RNAIMAX (Invitrogen, Carlsbad, CA). Cells were harvested and the luciferase activity was determined by using a dual-luciferase reporter assay system in accordance with the manufacturer's instructions (Promega, Madison, WI). Luciferase activities were normalized by co-transfection of the control Renilla luciferase plasmid, phRL-TK. The primer sequences were listed in Table S1.

Immunofluorescent Analysis

Liver samples were fixed in 4% paraformaldehyde (PFA) and processed by paraffin embedding and sectioning. Livers were embedded in identical orientation to allow direct comparison of tissue. The fixed tissues were dehydrated and embedded in paraffin. Tissue blocks were cut to 5 μm sections. After extensively washing with PBS, the slides were blocked with PBS-10% goat serum for 90 min. SOX9 antibody (AB5535, Millipore, USA), HOXB6 antibody (sc-166950, Santa Cruz Biotechnology), and hepatocyte nuclear factor (HNF) 4α antibody (Ab41898, Abcam, Cambridge, England) were diluted in PBS, and these antibodies were used to incubate sections at 4°C overnights. Slides were washed three times with PBS and incubated with corresponding secondary antibodies diluted in PBS for 1 hour, followed by PBS washes. The incubated slides were added with DAPI for nuclear staining and mounting. Images was acquired with a laser scanning confocal microscope (LSM710, Carl Zeiss Microscopy GmbH), were analyzed by Zen software with fixed parameters.

Western Blots

For whole-cell protein extraction, liver tissues were prepared in lysis buffer (Beyotime, China) according to the manufacturer's instructions. Protein lysates were separated by 10% or 12% SDS-PAGE (20 μg each lane). Next, the gel was transferred to polyvinylidene fluoride (PVDF) membranes (Millipore, USA). After being blocked with 5% skimmed milk or 5% BSA in Tris-buffered saline/Tween-20 (TBST), the membranes were incubated overnight with the anti-SOX9 (AB5535, Millipore, USA), anti-HOXB6 (sc-166950X, Santa Cruz Biotechnology), anti-GAPDH (60004-I-Ig, Proteintech, Chicago, USA), or Tag-3*Flag-antibody (66008-3-Ig, Proteintech, Chicago, USA) at 4°C. And then, the membranes were incubated with the corresponding HRP-conjugated secondary antibodies at room temperature for 1.5 hours. Finally, the membranes were visualized with enhanced chemiluminescence (ECL) (Bio-Rad, USA).

Lentivirus, Adenovirus, and Adeno-Associated Virus (AAV) Plasmid Construction and Production

The lentivirus plasmids and adenovirus plasmids were constructed and produced, as described previously. We initially screened candidate CRISPR gRNAs targeting sequences in sgRNA1 and -3 in mice upstream of the pre-miR-126a gene in NIH 3T3 cells. One of them formed a pair of sgRNAs with sgRNA2. The guide RNA targeting sgRNA3 displayed ~50% mutagenesis at the on-target site in *pre-miR-126a*, as determined by PCR and a T7 endonuclease 1 (T7EN1) cleavage assay. To completely overexpress HOXB6 in vivo, the pHBAAV-CMV-HOXB6-3flag-T2A-ZsGreen plasmid was constructed based on pHBAAV-CMV-MCS-3flag-T2A-ZsGreen (Hanbio, Shanghai, China) in which we inserted *Hoxb6*-CDS sequence into the multiple cloning site (CMV). The constructed plasmids were named pAAV-*Hoxb6* and

pAAV-ctrl. The two recombinant AAV plasmids were transfected into HEK293 cells with pAAV-RC and pHelper by using Lipofectamine 2000 transfection reagent (Invitrogen, Carlsbad, CA). AAV-*Hoxb6* and AAV-ctrl (Hanbio, Shanghai, China) were packaged in AAV-293 cells. The recombinant AAV in the AAV-293 cells were purified, and the titer of virus was measured by qRT-PCR. The titer range of stock solutions of AAV-*Hoxb6* and AAV-ctrl were 1.2~1.6X10^{12} vg/mL.

Isolation of Periportal (PP) and Perivenous (PV) Hepatocytes

Hepatocytes were obtained from the fed adult C57BL/6J male mice. Our experiments followed the procedures described previously with the usual digitonin collagenase perfusion technique. Intact mouse liver cells from the PV region were isolated by collagenase perfusion after first destroying the PP region by a brief portal infusion of digitonin. PP cells were isolated after retrograde digitonin infusion, as described previously (Berry and Friend, 1969; Gautam et al., 1987; Lindros and Penttila, 1985; Quistorff, 1985; Tordjmann et al., 1996).

Plasmid Construction

The 3'UTR of *Hoxb6*, which contained the predicted target site for *miR-126-5p*, was cloned into the psicheck-2 luciferase reporter vector which was cleaved at Not I and Xho I sites. The 3'UTR regions containing the potential binding sites and mutant sites of *Hoxb6* were cloned into the luciferase reporter system. The constructed vectors were designated WT and Mut. According to the published *Hoxb6* gene CDS sequence from GenBank, specific primers containing restriction enzyme sites were designed and amplified by PCR. After being digested with restriction endonucleases Kpn I and BamH I, the fragments were ligated into pcDNA3.1(+) to construct the HOXB6 overexpression vector. At ~3kb immediately upstream of the *SOX9* transcriptional start site, we constructed a series of truncated fragments into the vectors, then defined them as *SOX9*-Pro (~1.7kb, the region from -2242 to -535), *SOX9*-P1 (-2242 to -1888), *SOX9*-P2 (-1970 to -1669), *SOX9*-P3 (-1694 to 1407), *SOX9*-P4 (-1441 to 1126), *SOX9*-P5 (-1190 to -903), *SOX9*-P6 (-953 to -664), and *SOX9*-P7 (-771 to -535), *SOX9*-P6-1 (-953 to -832), *SOX9*-P6-2 (-873 to -748), *SOX9*-P6-3 (-790 to -664), *SOX9*-P6-3-1 (-790 to 737), *SOX9*-P6-3-2 (-749 to 704), and *SOX9*-P6-3-3 (-721 to -664). The primer sequences were listed in Supporting Table S4.

Supplemental References

Berry, M.N., and Friend, D.S. (1969). High-yield preparation of isolated rat liver parenchymal cells: a biochemical and fine structural study. The Journal of cell biology *43*, 506-520.

Gautam, A., Ng, O.C., and Boyer, J.L. (1987). Isolated rat hepatocyte couplets in short-term culture: structural characteristics and plasma membrane reorganization. Hepatology *7*, 216-223.

Lindros, K.O., and Penttila, K.E. (1985). Digitonin-collagenase perfusion for efficient separation of periportal or perivenous hepatocytes. The Biochemical journal *228*, 757-760.

Quistorff, B. (1985). Gluconeogenesis in periportal and perivenous hepatocytes of rat liver, isolated by a new high-yield digitonin/collagenase perfusion technique. The Biochemical journal *229*, 221-226.

Tordjmann, T., Berthon, B., Combettes, L., and Claret, M. (1996). The location of hepatocytes in the rat liver acinus determines their sensitivity to calcium-mobilizing hormones. Gastroenterology *111*, 1343-1352.
