# Supplementary material for: MiR-126 Regulates Properties of SOX9+ Liver Progenitor Cells during Liver Repair by Targeting Hoxb6
Source: Stem Cell Reports. 2020 Aug 6;15(3):706–20. doi: 10.1016/j.stemcr.2020.07.005 (PMC7486193; doi:10.1016/j.stemcr.2020.07.005)
Supplement: Document S2. Article plus Supplemental Information [file mmc2.pdf]

Stem Cell Reports
Article
OPEN ACCESS

MiR-126 Regulates Properties of SOX9+ Liver Progenitor Cells during Liver Repair by Targeting *Hoxb6*

Yi Yan,[1] Rui Wang,[1] Xiongji Hu,[1] Shichao Wang,[1] Liang Zhang,[1] Chenjiao Hou,[1] and Lisheng Zhang[1,*]
[1]College of Veterinary Medicine, Bio-medical Center, Huazhong Agricultural University, Wuhan, Hubei 430070, China
*Correspondence: lishengzhang@mail.hzau.edu.cn
https://doi.org/10.1016/j.stemcr.2020.07.005

SUMMARY

Liver progenitor cells (LPCs) have a remarkable contribution to the hepatocytes and ductal cells when normal hepatocyte proliferation is severely impaired. As a biomarker for LPCs, Sry-box 9 (*Sox9*) plays critical roles in liver homeostasis and repair in response to injury. However, the regulation mechanism of *Sox9* in liver physiological and pathological state remains unknown. In this study, we found that *miR-126* positively regulated the expression of *Sox9*, the proliferation and differentiation of SOX9+ LPCs by suppressing the translation of *homeobox b6* (*Hoxb6*). As a transcription factor, HOXB6 directly binds to the promoter of *Sox9* to inhibit *Sox9* expression, resulting in the destruction of the properties of SOX9+ LPCs in CCl_4-induced liver injury. These findings revealed the role of *miR-126* in regulating SOX9+ LPCs fate by targeting *Hoxb6* in liver injury repair. Our findings suggest the potential role of *miR-126* as a nucleic acid therapy drug target for liver failure.

INTRODUCTION

MicroRNAs (miRNAs), a class of endogenous, small non-coding RNAs composed of ∼22 nucleotides, bind to partial complementary sequences in the 3′ untranslated region (UTR) of their target transcripts and recruit RNA-induced silencing complex to inhibit the translation of these transcripts, they are involved in the regulation of cellular functions (Bartel, 2018; Mori et al., 2019). A recent study suggests that miRNAs, which are abundant in liver, can modulate a wide range of hepatocellular functions (Su et al., 2018). Our previous research showed that hepatic *miR-657* enhanced nuclear factor κB activity to promote hepatocellular carcinoma cell growth and transformation (Zhang et al., 2013). Moreover, *miR*-122 has been identified as a biomarker of acute liver failure in mice and humans (John et al., 2014; Luna et al., 2017). The newly reported *miR-221-3p* reduced secretion of CCR2 through post-transcription regulation of *Gnai2*, thus mitigating liver fibrosis (Tsay et al., 2019). The above-mentioned examples indicated that *miRNAs* play a critical role in liver physiological and pathological regulation. However, the specific roles of *miRNAs* in liver regeneration and repair, especially in regulating hepatic stem cell properties remain to be examined.

Our data showed that *miR-126* is encoded in the intron of *Egfl7* (Yan et al., 2020). Previous research reported that *miR-126* was highly expressed in normal hematopoietic stem cells (HSCs) and hematopoietic progenitor cells and restrained cell-cycle progression during hematopoiesis (Lechman et al., 2012). Recently, *miR*-126 has been reported to regulate the self-renewal of leukemia stem cells in chronic myelogenous leukemia (Zhang et al., 2018). Moreover, our previous research has shown that *miR-126* was involved in regulating liver aging. To be more specific, the knockdown of *miR-126* in bone marrow stromal cells (BMSCs) accelerated cell aging and inhibited hepatic repair functions of BMSCs (Yan et al., 2019). These results indicated that *miR*-126 might be involved in hepatic aging through liver stem cells (LSCs) or liver progenitor cells (LPCs). Although the roles of *miR-126* in stem cell function regulation and hepatic repair have been intensively studied, it remains largely unknown whether *miR-126* contributes to liver regeneration by regulating the LPC properties.

A few markers, including *Sox9*, *Axin2*, *Cd44*, and *Lgr5* were reported to be used to identify LSCs or LPCs (Huch et al., 2013; Wang et al., 2015). SOX9, a member of the sry-related high-mobility group box transcription factors, is closely related to cell proliferation and differentiation, and it regulates the stem cell homeostasis and differentiation (Ko et al., 2019; Mori-Akiyama et al., 2007). SOX9 is expressed throughout the biliary and pancreatic ductal epithelia (Alison and Lin, 2011). A previous study used *Sox9*-IRES-CreERT2 lineage tracing approach and found that the SOX9+ biliary compartment contributed to most new hepatocytes even during normal liver homeostasis. In addition, SOX9+ LPCs contributed to the formation of hepatocytes after liver injury (Furuyama et al., 2011; Tarlow et al., 2014). SOX9 was also weakly expressed in a population of periportal (PP) hepatocytes (named hybrid hepatocytes [HybHPs]) located in the portal triads of livers. HybHPs underwent extensive proliferation and replenished liver mass after chronic hepatocyte-depleting injury (Font-Burgada et al., 2015). The above-mentioned studies have demonstrated that SOX9 plays a critical role in liver regeneration and repair. However, the regulatory mechanisms of *Sox9* involved in liver regeneration and repair remain unclear.

This is an open access article under the CC BY license (http://creativecommons.org/licenses/by/4.0/).

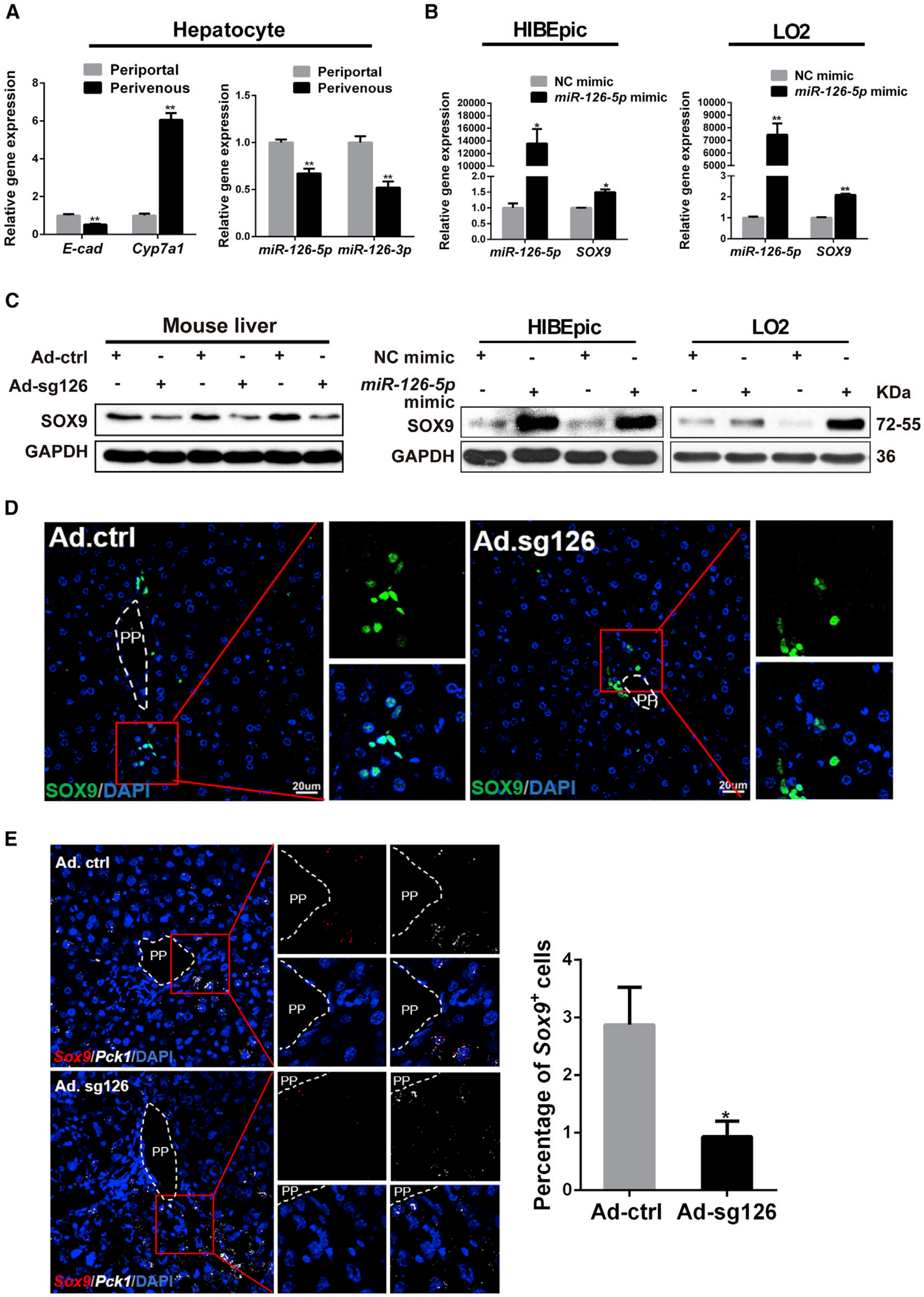

Figure 1. *miR-126* Regulates Liver Progenitor Cell Marker *Sox9* Expression and SOX9$^+$ Hepatocyte-like Cell Number

(A) Periportal (PP) hepatocytes and perivenous (PV) hepatocytes in 8-week-old mouse were isolated by using the digitonin/collagenase perfusion technique. Quantification of mature *miR-126-5p* and *miR-126-3p*, PP marker (*E-cad*), and PV marker (*Cyp7a1*) expressions in primary cultured PP hepatocytes and PV hepatocytes.

(legend continued on next page)

In this study, we examined the effect of *miR-126* as regulatory factors on the properties of SOX9+ LPCs. Moreover, we revealed the regulatory mechanism by which HOXB6 was involved in *miR-126*-mediated liver injury repair. Our data demonstrated that the interaction between *miR-126-5p* and *Hoxb6* mRNA induced post-transcriptional silencing, leading to the stable expression of *Sox9*, thereby maintaining the stem cell properties of SOX9+ LPCs during the liver repair.

RESULTS

MiR-126 Promotes SOX9 Expression and Induces SOX9+ LPCs

Recent investigation has revealed that *miRNAs* are involved in liver regeneration and might serve as the therapeutic approach to liver fibrosis (John et al., 2014; Tsay et al., 2019). Our previous research has shown that *miR-126* contributes to hepatic anti-aging and damage repair (Yan et al., 2019). To reveal the effect of *miR-126* on anti-aging, we first detected the expression of stem cell-associated genes in C3H10 cells from which the *miR*-126 has been deleted. The result showed that there were no significant changes in the expression levels of *Axin2*, *Cd44*, and *Lgr5*, but *Sox9* abundance was significantly reduced, compared with that of control C3H10 cells (Figure S1A). Then, we measured the expression of the previously reported hepatic PP (*E-cad*) and perivenous (PV) (*Cyp7a1*) zonation genes in the purified hepatocytes separated from mouse livers (Han et al., 2019; Pu et al., 2016; Rocha et al., 2015). Expression levels of *miR-126-5p* and *miR-126-3p* genes were significantly higher in the PP hepatocytes than in the PV hepatocytes (Figures 1A and S1B). Previous studies reported that there were much more SOX9-expressing hepatocytes detected in the PP area than in the PV area (Font-Burgada et al., 2015; Halpern et al., 2017). Based on these findings, we hypothesized that there might be a correlation between these two genes.

To confirm the hypothesis, *miR-126-5p* was overexpressed by transfecting the *miR-126-5p* mimics into HIBEpic and LO2 cells, and we found that SOX9 expression was dramatically increased at the mRNA and protein levels (Figures 1B and S1B). We also inhibited *miR-126* expression by infecting BMSCs with sg126-expressing lentivirus (sg126). The result showed that the expression of *Sox9* in BMSCs was significantly decreased after infection with sg126-expressing lentivirus (Figure S1C). To further investigate the effects of *miR-126* on SOX9 expression in the liver, we disrupted *miR*-126 by delivering sg126-expressing adenovirus (Ad.sg126) into mouse livers, and we found that the SOX9 level in the Ad.sg126 group was much lower than that in the control adenovirus (Ad.ctrl) group by western blot (Figure 1C). Interestingly, a drastic decrease in the number of SOX9+ hepatocyte-like cells was observed around the PP area in *miR-126*-deleted mouse liver (Figures 1D and 1E).

Hoxb6 Is a Target of *miR-126-5p*

To identify putative targets of *miR-126-5p* contributing to the increase in SOX9+ LPCs in liver, we searched the candidate target genes by TargetScan and miRbase. Among various potential targets, we focused on *Hoxb6* gene since SOX9 was continuously expressed in *Hoxb6* mutant pancreata (Larsen et al., 2015), and *Hoxb6* regulates the generation, proliferation, or survival of erythroid progenitor cells in fetal livers, and disruption of the *Hoxb6* resulted in increased numbers of early erythrocyte progenitors (Kappen, 2000). So, we hypothesized that *miR-126* induces SOX9+ progenitors through *Hoxb6*. The 3′ UTR of the *Hoxb6* gene contains binding sites for *miR-126-5p* (Figure 2A). To determine whether *Hoxb6* is a direct target of *miR-126-5p*, we constructed luciferase reporter vector in which the *Hoxb6* 3′ UTR is placed behind the luciferase gene. We found that *miR-126-5p* inhibited luciferase activity, whereas no inhibition was observed when the *miR-126-5p* target site was mutated (Figures 2A, 2B, and S2A). Consistently, miRNA mimics-mediated overexpression of *miR-126-5p* suppressed the expression of HOXB6 in HIBEpic cells and HepG2 cells (Figures 2C and S2B). However, antagomir-mediated inhibition of *miR-126-5p* promoted the expression of HOXB6 (Figure S2B). Furthermore, lentivirus-mediated deletion of *miR-126* resulted in the upregulation of HOXB6 protein level in BMSCs and C3H10 cells (Figure S2C).

(B) HIBEpic and LO2 cells were transfected with *miR-126-5p* mimics or negative control (NC) mimics for 48 h. Quantification of *miR-126-5p* and *SOX9* expressions in NC group and *miR-126-5p* group. The protein level of SOX9 was detected by western blotting.
(C) Expression of SOX9 was measured at the protein level in Ad.ctrl and Ad.sg126 mouse livers.
(D) Immunofluorescence was performed on the sections from paraffin-embedded tissue samples of Ad.ctrl mouse livers and Ad.sg126 mouse livers. Immunofluorescence of SOX9 was performed. DAPI (blue) shows nuclei. Scale bar represents 20 μm.
(E) Representative images from RNAscope assays for *Sox9* mRNA levels and quantification of *Sox9*+ cells in livers of Ad.ctrl and Ad.sg126 mice. Red presents *Sox9*. White (*Pck1*) marks hepatocytes in the PP zone. Scale bar represents 20 μm.
Data are expressed as means ± SD, n = 3 independent experiments containing three replicates (A and B), n = 6 mice per group containing three replicates (C–E). Significant difference is presented at the levels of $^*p < 0.05$ and $^{**}p < 0.01$ by two-tailed Student's t test. See also Figure S1.

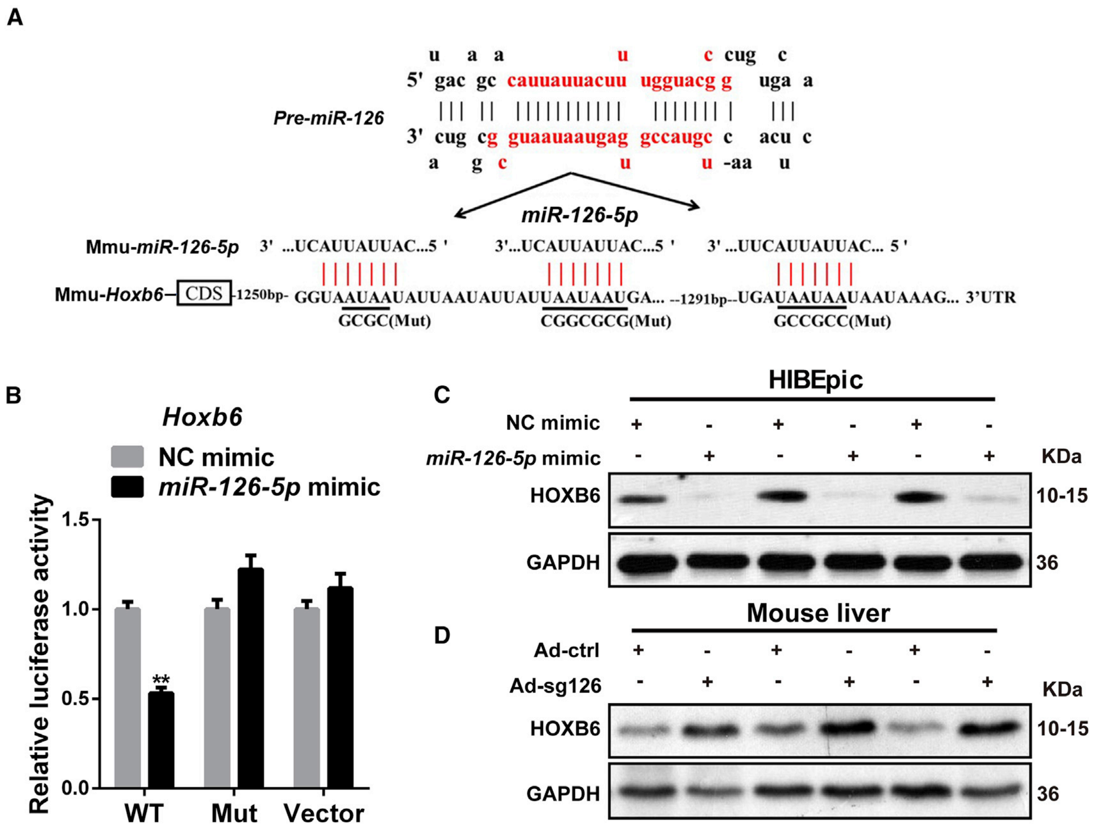

Figure 2. *Hoxb6* Is a Target of *miR-126-5p*

(A) The 3′ UTR of *Hoxb6* gene contains binding sites for *miR-126-5p* according to TargetScan (http://www.targetscan.org/). Black underlined sequences indicate the point mutations used to generate mouse *Hoxb6* 3′ UTR Mut constructs.
(B) Relative luciferase activity assays of luciferase reporter plasmid with mouse WT or Mut *Hoxb6* 3′ UTR, and that of vector were performed after co-transfection with *miR-126-5p* mimics or NC mimics in HeLa cells. *Hoxb6* 3′ UTR Mut seed complementary sites were shown in (A).
(C) HOXB6 protein levels were measured by western blotting after transient transfection with *miR-126-5p* mimics or NC into HIBEpic cells.
(D) Expression of HOXB6 was measured at the protein level in Ad.ctrl and Ad.sg126 mouse livers.
Data are expressed as means ± SD, n = 3 independent experiments containing three replicates. Significant difference is presented at the level of $^{**}p < 0.01$ by two-tailed Student's t test. See also Figure S2.

Consistent with the *in vitro* data, western blot analysis of the liver samples showed that deletion of *miR-126* increased hepatic HOXB6 levels (Figure 2D). Taken together, these results demonstrated that *miR-126-5p* directly targeted *Hoxb6*.

HOXB6 Negatively Regulates SOX9 Expression *In Vitro* and *In Vivo*

Based on the results described above, we explored whether HOXB6 regulated the expression of SOX9. *HOXB6* siRNA (Si-*HOXB6*) or PcDNA3.1-*HOXB6* was transfected into HepG2, HIBEpic, and LO2 cells to reduce or enhance HOXB6 function, respectively. Expression level of SOX9 was significantly increased in Si-*HOXB6*-transfected HepG2, HIBEpic, and LO2 cells (Figure 3A), whereas SOX9 was significantly reduced in PcDNA3.1-*HOXB6*-transfected HepG2 and HIBEpic cells (Figure 3B), indicating that HOXB6 suppressed SOX9 expression *in vitro*.

To confirm this observation, HOXB6 was overexpressed by tail vein injection of the adeno-associated virus (AAV)-*Hoxb6* or AAV control into mouse livers. We found that overexpression of HOXB6 significantly reduced *Sox9* mRNA abundance compared with the control group (Figure 3C). In line with the mRNA levels, SOX9 was even more profoundly downregulated at the protein level in the AAV-*Hoxb6* group (Figure 3C). Taken together, our data indicated that HOXB6 negatively regulated the expression of the LPC gene *Sox9*.

HOXB6 Negatively Regulates *SOX9 trans*-Activity

HOXB6 and other *HOX* genes have been thought to function as transcription factors, and they bind to DNA targets

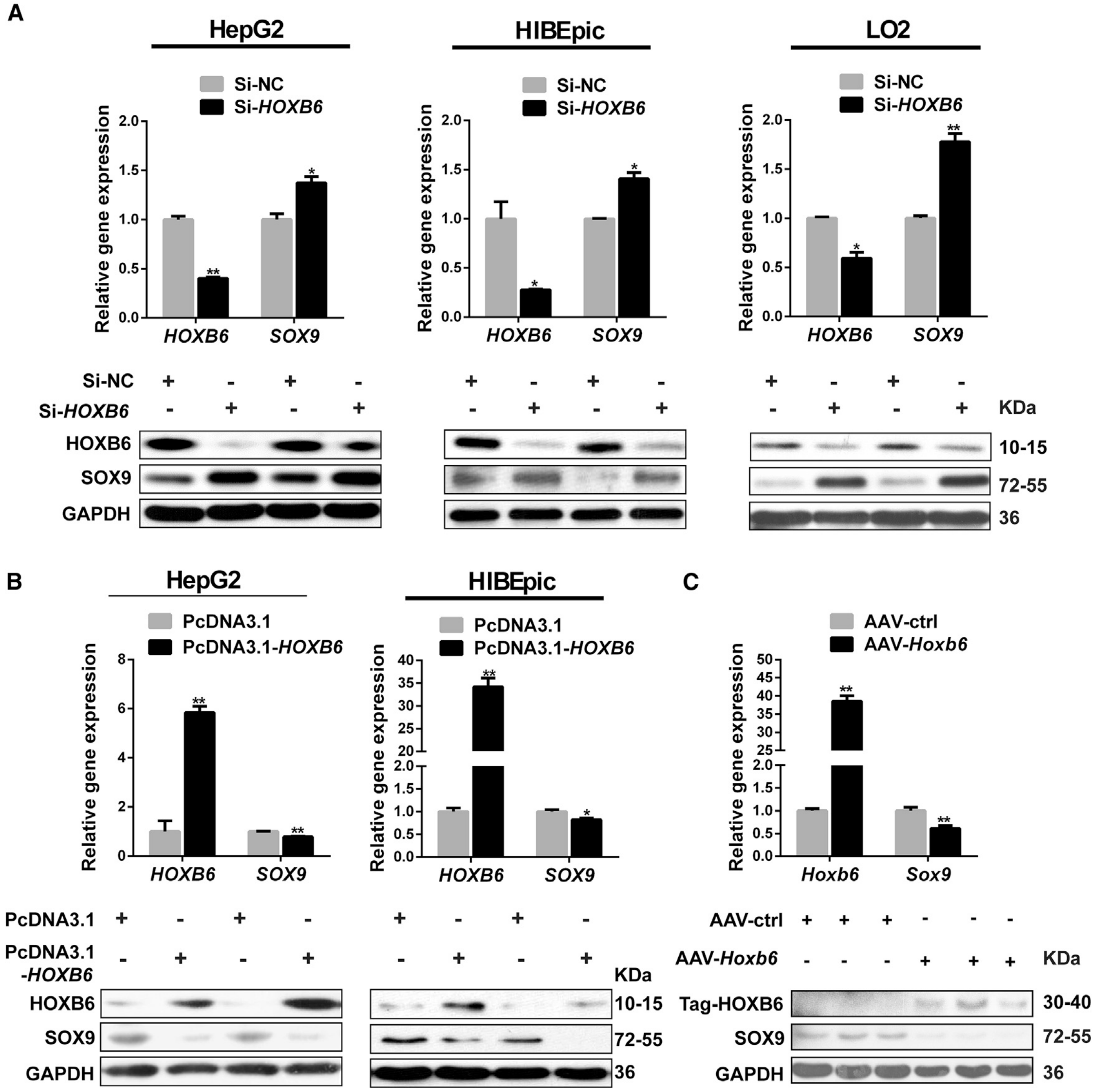

Figure 3. HOXB6 Negatively Regulates SOX9 Expression *In Vitro* and *In Vivo*

(A) HepG2 cells, HIBEpic cells, and LO2 cells were transfected with the *HOXB6* siRNA (Si-*HOXB6*) or NC. At 48 h after transfection, western blotting and qRT-PCR analysis of HOXB6 and SOX9 expression in the NC group and the Si-*HOXB6* group were performed.

(B) Western blotting and qRT-PCR analysis were conducted to detect HOXB6 and SOX9 expression in HepG2 and HIBEpic cells after HOXB6 exogenous overexpression.

(C) Western blotting and qRT-PCR analysis of HOXB6 and SOX9 expression in AAV-ctrl and AAV-*Hoxb6* mice livers were performed.

Data are expressed as means ± SD, n = 3 independent experiments containing three replicates (A and B), n = 6 mice per group containing three replicates (C). $^{*}p < 0.05$ and $^{**}p < 0.01$ by two-tailed Student's t test.

containing a TAAT sequence (Porcelli et al., 2019). Based on the description above, we hypothesized that HOXB6 could regulate *SOX9* transcription. We next attempted to identify the binding sites of HOXB6 by serially truncating regions within the *SOX9* promoter. At ~3 kb immediately upstream of the *SOX9* transcriptional start site, we identified six potential TAAT-HOXB6 binding sites in the *SOX9* promoter region from −2,242 to −535 bp (Figure S3A). Next, we constructed a series of truncated promoter fragments aimed at removing these sites in a stepwise manner. As shown in Figures 4A and S3B, human or mouse *SOX9* promoter (*SOX9*-Pro), a full-length construct, exhibited a dramatic decrease in transcriptional activity by HOXB6 overexpression.

To determine the binding site in which the transcriptional activity of *SOX9* promoter was inhibited by

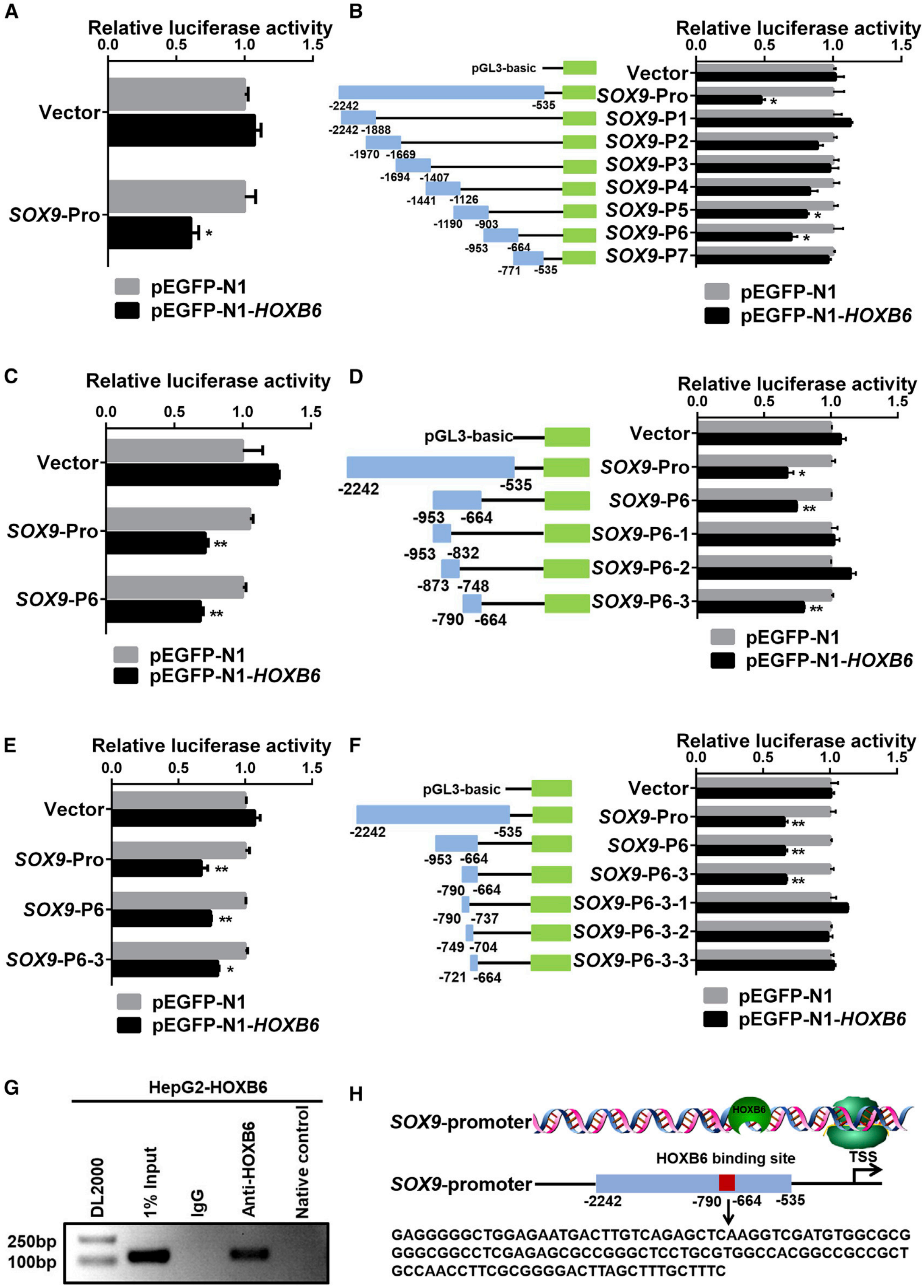

Figure 4. HOXB6 Negatively Regulates *SOX9 trans*-Activity

(A) Relative luciferase activity analysis of the *SOX9* promoter-reporter constructs. pGL3-basic plasmid with a 1,700-base-pair fragment of the human *SOX9* promoter (position −2,242 to −535 relative to the transcription start site, *SOX9*-Pro) was transfected into HEK293T cells with the phRL-TK and pEGFP-N1 or pEGFP-N1-*HOXB6* eukaryotic expression plasmids.

(legend continued on next page)

HOXB6, seven ~300-bp *SOX9* promoter-truncated fragments were constructed using *SOX9*-Pro as a template. These seven constructs were named *SOX9*-P1, *SOX9*-P2, *SOX9*-P3, *SOX9*-P4, *SOX9*-P5, *SOX9*-P6, and *SOX9*-P7 (Figure 4B). As shown in Figure 4B, luciferase activity of *SOX9*-P5 and *SOX9*-P6 were observed to be inhibited by HOXB6. But *SOX9*-P6 demonstrated a dramatically decreased transcriptional activity by HOXB6. Thus, these results revealed that the *SOX9*-P6 (−953 to −664 bp) region was responsible for *SOX9* transcriptional activity inhibition by HOXB6, and HOXB6 had the same effect on *SOX9*-P6 and *SOX9*-Pro (Figure 4C). Next, we constructed a series of *SOX9* promoter-truncated fragments with *SOX9*-P6 as a template. We named the three constructs *SOX9*-P6-1 (−953 to −832), *SOX9*-P6-2 (−873 to −748), and *SOX9*-P6-3 (−790 to −664) (Figure 4D). We detected the reduction in luciferase activity of the constructs containing only *SOX9*-P6-3 (−790 to −664) regions after overexpression of HOXB6 (Figures 4D and 4E). However, no reduction in luciferase activity was observed from three truncated fragments containing *SOX9*-P6-3-1 (−790 to −737), *SOX9*-P6-3-2 (−749 to −704), or *SOX9*-P6-3-3 (−721 to −664) promoter regions after overexpression of HOXB6 (Figure 4F).

To confirm the binding sites of HOXB6 to the *SOX9* promoter, we performed a chromatin immunoprecipitation (ChIP) assay. The crosslinked extracts were immunoprecipitated with antibodies against HOXB6 or control anti-IgG antibody. The crosslinked DNA was analyzed by using PCR with the primers designed to amplify the HOXB6-responsive region containing the *SOX9*-P6-3 (−790 to −664). HOXB6 was determined to be associated with the *SOX9* promoter region containing the SOX9-P6-3 fragments (Figure 4G). Taken together, our results suggested that *SOX9* was directly negatively regulated by HOXB6 with the binding sequences of *SOX9* promoter by HOXB6 shown in Figure 4H.

AAV-Mediated Overexpression of HOXB6 Suppresses Proliferation and Differentiation of SOX9$^+$ LPCs to Aggravate Damage in a CCl_4 Chronic Liver Injury Model

Based on the above-mentioned regulation mechanism *in vitro*, an *in vivo* experiment was performed to reveal the mechanism by which HOXB6 regulates SOX9 in CCl_4-induced liver injury model. To elucidate the regulation mechanism in liver, we first demonstrated the expression of HOXB6 and SOX9 in primary hepatocyte (Figure S4). Compared with control group, CCl_4 treatment group exhibited a higher level of SOX9 expression (Figure 5A). After tail vein injection of 1×10^{10} particles of AAV-mediated overexpression of HOXB6 into the CCl_4-treated mice, the expression of SOX9 was strongly suppressed at mRNA and protein levels (Figure 5A). Hematoxylin and eosin (H&E) staining results indicated that CCl_4 treatment induced hepatotoxicity (Figure 5B). In addition, Masson and Sirius red staining results showed that CCl_4 treatment promoted liver fibrosis (Figure 5B). At the same time, the levels of aspartate aminotransferase (ALT), alanine aminotransferase (AST), and lactic dehydrogenase (LDH) were observed significantly higher in the CCl_4 group than in the control group (Figure 5C). Serological and histological analysis revealed that the treatment with AAV-*Hoxb6* resulted in a significantly increased liver damage and fibrosis as well as a significant increase in ALT, AST, and LDH levels (Figures 5B and 5C). Our results indicated that AAV-mediated overexpression of HOXB6

(B) Relative luciferase activity assays were performed in HEK293T cells by transiently co-transfecting *SOX9*-Pro (−2,242 to −535), *SOX9*-P1 (−2,242 to −1,888), *SOX9*-P2 (−1,970 to −1,669), *SOX9*-P3 (−1,694 to −1,407), *SOX9*-P4 (−1,441 to −1,126), *SOX9*-P5 (−1,190 to −903), *SOX9*-P6 (−953 to −664), *SOX9*-P7 (−771 to −535), and vector with expression plasmids of pEGFP-N1-*HOXB6* or pEGFP-N1.
(C) Relative luciferase activity assays were performed in HEK293T cells by transiently co-transfecting *SOX9*-Pro (−2,242 to −535), *SOX9*-P6 (−953 to −664), and vector in a combination of expression plasmids of pEGFP-N1 or pEGFP-N1-*HOXB6*.
(D) *SOX9*-P6 (−953 to −664) as well as various truncated fragments were inserted into pGL3-basic plasmid with luciferase reporter genes, including *SOX9*-P6-1 (−953 to 832), *SOX9*-P6-2 (−873 to −748), and *SOX9*-P6-3 (−790 to −664). Relative luciferase activity assays were performed in HEK293T cells by transiently transfecting the above plasmids with pEGFP-N1 or pEGFP-N1-*HOXB6*.
(E) Relative luciferase activity assays were performed in HEK293T cells by transiently transfecting *SOX9*-Pro, *SOX9*-P6, *SOX9*-P6-3, and vector in a combination of expression plasmids of pEGFP-N1 or pEGFP-N1-*HOXB6*.
(F) *SOX9*-P6-3 (−790 to −664) as well as various truncated fragments were inserted into the pGL3-basic plasmid with luciferase reporter gene, including *SOX9*-P6-3-1, *SOX9*-P6-3-2, and *SOX9*-P6-3-3.
(G) Chromatin immunoprecipitation-PCR analysis. Chromatin was prepared and immunoprecipitated with specific antibodies against HOXB6 or IgG. The input DNA and DNA isolated from the precipitated chromatin were amplified by PCR using primers spanning the HOXB6 binding site, and the obtained PCR product was separated on a 1.5% agarose gel. Lanes: 1, marker; 2, input; 3, IgG; 4, HOXB6 antibody; 5, negative control.
(H) Sequence of the *SOX9* promoter region. The listed nucleotide is the HOXB6 binding site.
Data are expressed as means ± SD, n = 3 independent experiments containing three replicates. Significant difference is presented at the levels of *p < 0.05 and **p < 0.01 by two-tailed Student's t test. See also Figure S3.

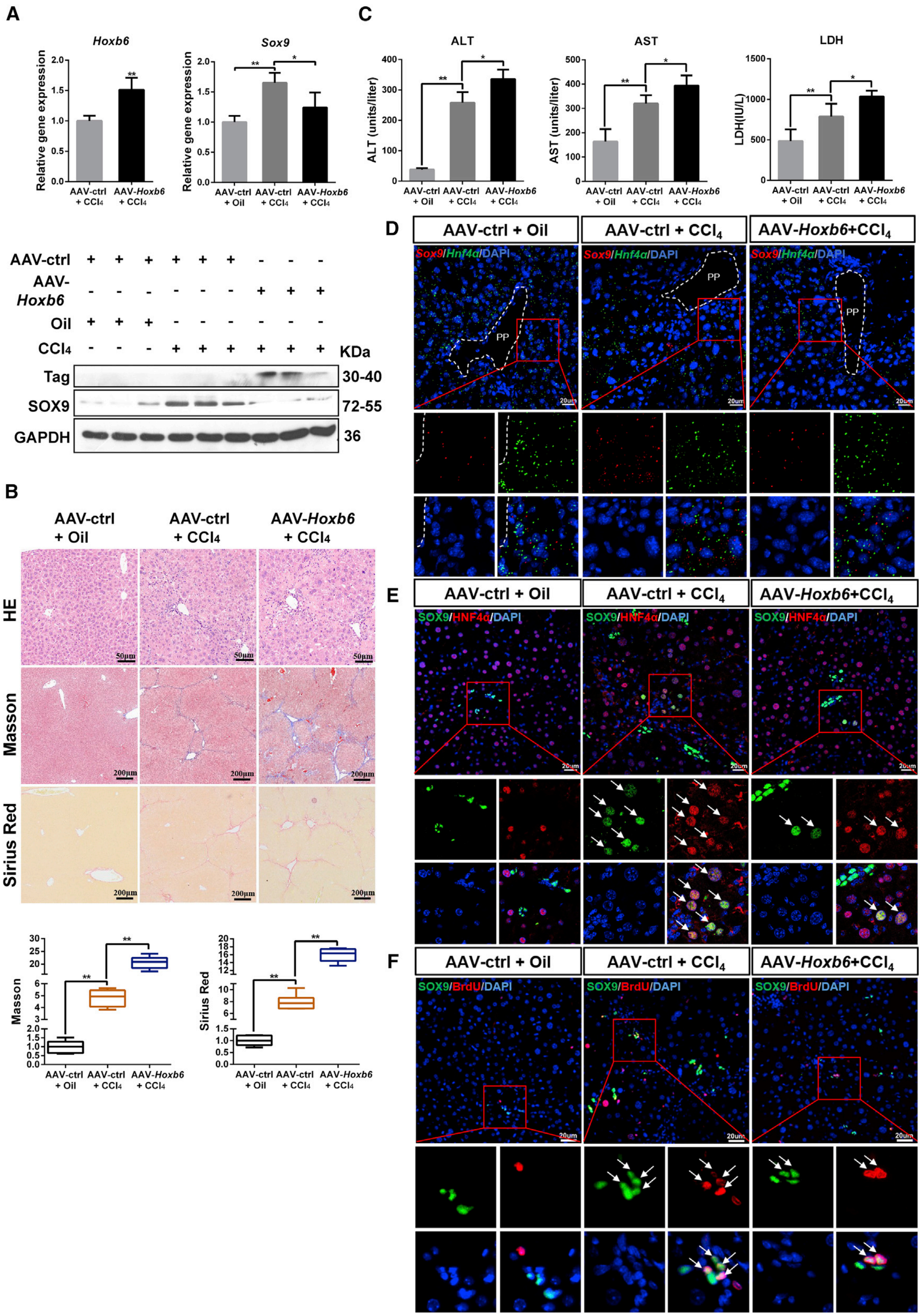

(legend on next page)

was able to obviously aggravate liver injury caused by CCl_4.

To investigate the effect of HOXB6 on liver regeneration and injury repair through regulation of SOX9, we analyzed the behavior of $SOX9^+$ cells during the regeneration period. Since $SOX9^+/HNF4\alpha^+$ HybHPs was regenerated in the injured liver without developing into cancer (Font-Burgada et al., 2015), we focused on the *Hoxb6* effects on the HybHPs. *Hnf4α* and *Sox9* were co-stained to assess $Sox9^+$ cell differentiation using an RNAscope assay. We found that the number of $Sox9^+/Hnf4\alpha^+$ hepatocytes around the PP area in the CCl_4 treatment group was larger than that in the control group. After AAV-*Hoxb6* treatment, CCl_4-injured mice presented a smaller number of $Sox9^+/Hnf4\alpha^+$ hepatocytes than the AAV control group (Figure 5D). The above results were also consistent with immunofluorescence results that HOXB6 decreased the number of $SOX9^+$ hepatocytes (Figure 5E).

To investigate whether HOXB6 regulated $SOX9^+$ LPC proliferation, we performed proliferation analysis in liver sections. Immunofluorescent double staining of the proliferation marker bromodeoxyuridine (BrdU) and SOX9 results showed an obvious increased proliferation of $SOX9^+$ LPCs in the livers of the CCl_4 injury group compared with that in control livers. After treatment with AAV-*Hoxb6*, a significant decrease in proliferation of $SOX9^+$ LPCs was seen in the livers of the CCl_4 injury group (Figure 5F). Collectively, these data demonstrated that overexpression of HOXB6 suppressed $SOX9^+$ stem cell proliferation and propagation.

Taken together, our data indicated the downregulation of the LPC marker *Sox9* and the resultant decreased proliferation and differentiation of $SOX9^+$ LPCs after HOXB6 exogenous expression in CCl_4 chronic liver injury.

Adenovirus-Mediated Deletion of *miR-126* Suppresses Proliferation and Differentiation of $SOX9^+$ LPCs to Aggravate Damage in CCl_4 a Chronic Liver Injury Model by Targeting *Hoxb6*

To further examine whether *miR-126* regulated *SOX9* by targeting *HOXB6*, HepG2 cells were transfected with negative control mimics, *miR-126-5p* mimics, PcDNA3.1, or PcDNA3.1-*HOXB6*, respectively. The qRT-PCR and western blot results revealed that HOXB6 overexpression abrogated the *miR-126-5p* mimics-induced promotion of SOX9 levels, suggesting that *miR-126-5p* regulated SOX9 by targeting HOXB6 (Figure 6A). The above results were consistent with in the cell line C3H10, from which the *miR*-126 has been deleted (Figure S5A).

We further studied *Sox9* regulation by *miR-126 in vivo* in a CCl_4-induced chronic liver injury model. Mice were repeatedly administered a low dose of CCl_4 to induce chronic liver injury. After tail vein injection of 1×10^{10} particles of Ad.ctrl or Ad.sg126 into the CCl_4-treated mice, the expression of *miR-126* in liver was reduced (Figures S5B and S5C). To investigate whether *miR-126* contributed to liver injury repair, we collected the livers and blood for further analysis. Compared with the control group, the CCl_4 treatment group exhibited a higher expression level of SOX9 and a lower level of HOXB6 (Figure 6B). After deletion of *miR-126* in the CCl_4-treated mice, the drastically decreased expression of SOX9 and the increased expression of HOXB6 were observed (Figure 6B). We found that CCl_4 treatment induced hepatotoxicity. Serological and histological analysis revealed that, after treatment with Ad.sg126, a significant increase in the severity of liver damage and fibrosis and in ALT and AST levels in the CCl_4 injury group was observed (Figure 6C). In addition, the deletion of *miR-126* promoted a CCl_4-induced increase in liver/body weight ratio (Figure S5D). These results

Figure 5. AAV-Mediated Overexpression of HOXB6 Suppresses the Proliferation and Differentiation of $SOX9^+$ LPCs to Aggravate Damage in a CCl_4 Chronic Liver Injury Model

AAV-ctrl and AAV-*Hoxb6* were administered to 8-week-old C57 mice via tail vein injection. After 2 weeks, mice were treated with CCl_4 (diluted in oil with 1:4) twice per week for 4 weeks, then livers and blood were collected.

(A) Hepatic expression levels of SOX9 and HOXB6 were measured in AAV-ctrl, AAV-HOXB6 mice after CCl_4 injury by qRT-PCR and western blot.

(B) Representative H&E, Masson, and Sirius red staining in AAV-ctrl mice liver and AAV-*Hoxb6* mice liver after chronic hepatic injury by CCl_4 treatment. Fibrosis was quantified by morphometric measurement of Masson and Sirius red.

(C) Serum ALT, AST, and LDH levels of AAV-ctrl mice and AAV-HOXB6 mice after chronic hepatic injury by CCl4 treatment.

(D) Representative images from RNAscope assays of *Hnf4α* and *Sox9* in livers of AAV-ctrl and AAV-*Hoxb6* mice after CCl_4 injury. Red presents *Sox9*. Blue (DAPI) shows nuclei. Green (*Hnf4α*) marks hepatocytes. Scale bar represents 20 μm.

(E) SOX9 and HNF4α double staining in periportal areas in the livers of AAV-ctrl and AAV-*Hoxb6* mice after CCl_4 injury. Blue (DAPI) shows nuclei. Red (HNF4α) marks hepatocytes. Green presents SOX9. Scale bar represents 20 μm.

(F) SOX9 and BrdU double staining in periportal areas in livers of AAV-ctrl and AAV-*Hoxb6* mice after CCl_4 injury. Blue (DAPI) shows nuclei. Red (BrdU) marks the proliferating cells. Green presents SOX9. Scale bar represents 20 μm.

Data are expressed as means ± SD, n = 6 mice per group containing three replicates. Significant difference is presented at the levels of $^*p < 0.05$ and $^{**}p < 0.01$ by two-tailed Student's t test. See also Figure S4.

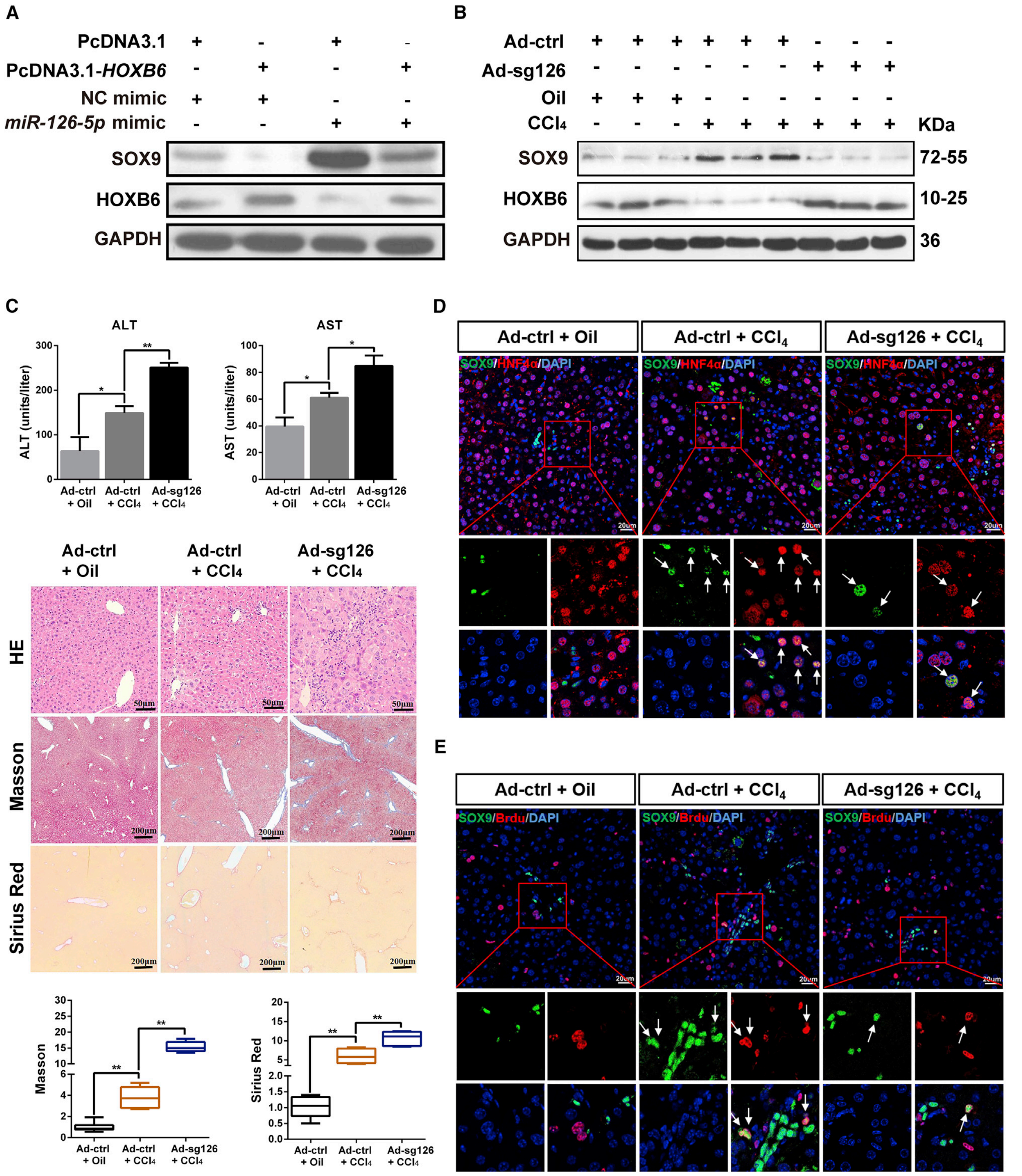

Figure 6. Adenovirus-Mediated Deletion of *miR-126* Suppresses the Proliferation and Differentiation of SOX9$^+$ LPCs to Aggravate Liver Damage through *Hoxb6* in a CCl_4 Chronic Injury Model

(A) PcDNA3.1 or PcDNA3.1-*HOXB6* was co-transfected with *miR-126-5p* mimics or NC into HepG2 cells, respectively. HOXB6 and SOX9 levels were measured by western blotting.

(legend continued on next page)

indicated that deletion of *miR-126* could obviously aggravate liver injury by targeting *Hoxb6*.

To investigate whether *miR-126* affected liver regeneration and injury repair through *Sox9*, we analyzed the behavior of $SOX9^+$ stem cells during the regeneration period after CCl_4-induced liver injury. We found that the number of $SOX9^+/HNF4\alpha^+$ hepatocytes around the PP area in the CCl_4 administration group was larger than in the control group. After Ad.sg126 treatment, CCl_4-injured mice presented a smaller number of $SOX9^+/HNF4\alpha^+$ hepatocytes (Figure 6D). These results suggested that the deletion of *miR-126* suppressed the differentiation of $SOX9^+$ LPCs into hepatocytes. Furthermore, immunofluorescent double staining of BrdU and SOX9 showed an obvious increase in $SOX9^+$ LPC proliferation in the livers of the CCl_4 injury group compared with control livers. After treatment with Ad.sg126, an obvious decrease in $SOX9^+$ LPC proliferation in the livers of CCl_4 injury group was observed (Figure 6E). The above results indicated that the deletion of *miR-126* suppressed $SOX9^+$ LPC proliferation. Taken together, our data suggested that upregulation of the LPC marker *Sox9* and the resultant increased proliferation and differentiation of $SOX9^+$ LPCs possibly explained why *miR*-126 might contribute to liver regeneration and repair.

DISCUSSION

The liver plays a pivotal role in the metabolism and detoxification of xenobiotics, which increases its possibility to be subjected to toxic damage, resulting in rapid loss of hepatic function, and high risk of losing regenerative and repair capacity (Font-Burgada et al., 2015). One previous study has shown that *miR*-302b and *miR-20a* repress hepatic functions by regulating transforming growth factor β (TGF-β) (Wei et al., 2013). Other previous studies have reported that *miRNAs* functioned by stimulating hepatocytes or by repressing cholangiocyte gene expression in mature hepatocytes (Gailhouste et al., 2013; Laudadio et al., 2012; Rogler et al., 2009). Our study found that *miR-126* was expressed in hepatocytes and biliary epithelial cells, and it was necessary for maintaining $SOX9^+$ LSCs properties. Thus, the molecular mechanisms of *miR-126* regulation in LSCs are important for understanding liver repair.

LSCs or LPSs play important roles in the generation of hepatocytes and cholangiocytes (Suzuki et al., 2000). During persistent and severe liver damage, part of hepatocytes undergo dedifferentiation into LPCs for liver regeneration (Itoh and Miyajima, 2014). Although it has been well reported that the properties of LPCs were regulated by a number of intracellular and extracellular signaling pathways (Kitade et al., 2013; Parent and Beretta, 2008), the relationships among these signaling pathways and the contribution of *miR-126* in LPCs are largely unknown. To identify possible *miR-126* target genes involved in the regulation of LPCs properties, we performed a computational screen for genes with complementary sites of *miR-126* in their 3′ UTR by using open-access software. Here, we identified *Hoxb6* to be a *miR-126* target gene involved in regulating *Sox9* levels.

The *Hox* family of homeobox genes, as major transcriptional regulators in the body, encodes DNA binding proteins that play a crucial role in early body morphogenesis and hematopoietic development, and *Hox* family exhibits important effects on stem cell renewal, lineage commitment, and differentiation (Amsellem et al., 2003; Krumlauf, 1994; Larsen et al., 2015; Thorsteinsdottir et al., 1997). *Hoxb6*, as a member of the *Hox* family, was found to regulate HSC self-renewal, and its overexpression in mice resulted in HSC and myeloid progenitor cell expansion (Bhatlekar et al., 2018; Fischbach et al., 2005). However, HOXB6 functions in liver and the related molecular signals and mechanisms have not been investigated. The cell types that could respond to HOXB6 activation also remain unknown. The present study demonstrated that HOXB6 inhibited $SOX9^+$ LPC proliferation and differentiation by reducing SOX9 levels in liver injury repair. Our results are also consistent with previous research findings that $SOX9^+$ LPCs were a preexisting group around the PP area with the high regenerative and proliferative capability (Font-Burgada et al., 2015; Li et al., 2016), and the ability to

(B) Hepatic expression levels of SOX9 and HOXB6 were measured by western blot in CCl_4-injured mice following CRISPR/Cas9-mediated *miR-126* gene disruption.
(C) Hepatotoxicity and ALT/AST enzymes activities change in CCl_4-injured mice following *miR-126* gene disruption. Representative H&E, Masson, and Sirius red staining in Ad-ctrl- and Ad-sg126-treated mice liver after chronic hepatic injury by CCl_4. Fibrosis was quantified by morphometric measurement of Masson and Sirius red.
(D) SOX9 and HNF4α double staining in periportal areas in livers of Ad-ctrl- or Ad-sg126-treated mice after CCl_4 injury. Blue (DAPI) shows nuclei. Red (HNF4α) marks hepatocytes. Green presents SOX9. Scale bar represents 20 μm.
(E) SOX9 and BrdU double staining in periportal areas in livers of Ad-ctrl- or Ad-sg126-treated mice after CCl_4 injury. Blue (DAPI) shows nuclei. Red (BrdU) marks the proliferating cells. Green presents SOX9. Scale bar represents 20 μm.
Data are expressed as means ± SD, n = 6 mice per group containing three replicates. Significant difference is presented at the levels of $^*p < 0.05$ and $^{**}p < 0.01$ by two-tailed Student's t test. See also Figure S5.

differentiate into hepatocytes in livers (Ko et al., 2019). These studies support our results that upregulation of SOX9 promoted SOX9+ LPC proliferation and differentiation during liver fibrosis.

SOX9 has been reported to be regulated in a variety of cytokines and signaling pathways in different tissues. The deletion of *BMP type I receptor* gene in chondro-osteo progenitor cells led to chondrodysplasia and reduction in SOX9 (Yoon et al., 2005). In addition, SOX9 was induced by TGF-β in the kidney fibroblast, and it acted as an important downstream mediator of TGF-β signaling to promote renal fibrosis (Li et al., 2018). However, the regulation mechanism of *Sox9* transcription in the progression of liver injury repair remained unknown. Considering the principal role of HOXB6 in the HSC self-renewal and differentiation (Bhatlekar et al., 2018), and the fact that the HOXB6 protein repressed globin transcript levels in a DNA binding-dependent manner, thus repressing the erythroid phenotype in human leukemic cells (Shen et al., 2004), we investigated the effects of HOXB6 on SOX9 expression and properties of SOX9+ LPCs.

Although HOX homeodomain proteins were thought to function as transcription factors, most full-length HOX proteins, including HOXB6, bound only weakly to DNA targets containing a TAAT sequence, and the transcription of target genes was weakly activated or repressed (Catron et al., 1993; Graba et al., 1997; Sánchez-Higueras and Rastogi, 2019). In addition, in one previous study, transient reporter gene analysis results revealed that HOXB6 and other HOX proteins did not change the activity of luciferase reporter vectors containing synthetic TAAT multimers or putative gene regulatory regions (Shen et al., 2001). Similarly, in this study, our observations provided evidence that a binding region of HOXB6 was in the upstream of *SOX9* promoter, and that HOXB6 was responsible for the downregulation of SOX9 in hepatocytes and cholangiocytes. However, we found that HOXB6 did not change the luciferase activity in transient reporter gene assays using putative *SOX9* regulatory regions containing a TAAT sequence, but luciferase activity was found to be changed in *SOX9* promoter regions containing no TAAT sequences (*SOX9*-P5 and *SOX9*-P6). This result indicated that HOXB6 might exert transcriptional repression by binding to other specific sequences in the downstream target gene promoter region, which requires further study in the future. These findings indicated that HOXB6 affected the proliferation and differentiation of SOX9+ LPC by transcriptionally regulating the expression of SOX9. To our knowledge, this is the first attempt to report a LPC regulatory event during liver regeneration and repair.

Furthermore, the overall contribution of *miR-126* to the whole repair process, especially to liver injury repair, remains elusive. In this study, we found that, during liver injury repair, the expression of HOXB6 increases with deletion of *miR-126*, which leads to the inhibition of proliferation and differentiation in SOX9+ LPCs. In addition, we overexpressed *miR-126* or/and *HOXB6* in liver cancer cell lines, respectively, and we found that overexpression of HOXB6 abrogated *miR-126*-induced increases in stem cell-related gene *SOX9* expression. These results suggest that *miR-126-5p* can restrict the inhibition effect of HOXB6 on SOX9+ LPCs properties, thus contributing to hepatic repair and regeneration.

The *miR*-126 has broad biological and physiological implications. Thus, it is vital to fully understand the biological and physiological properties and functions of *miR-126*. In conclusion, we have identified *miR-126* as a regulator of stem cell-related gene *Sox9* expression and SOX9+ LPCs properties in liver repair by targeting *Hoxb6*. Our principal findings may have a clinical implication for treating liver diseases. In future studies, the molecular mechanisms of regulating the expression and function of *miR-126* in hepatocytes and cancer cells require further clarification and analysis. Knowledge of the molecular properties of liver injury repair and carcinogenesis will contribute to the development of anticarcinogenic agents.

EXPERIMENTAL PROCEDURES

Detailed methods are provided in the Supplemental Information.

Mice and Injury Regimens

Adult C57BL/6J male mice were given standard rodent chow and water *ad libitum* under a standard 12-h light/dark cycle. For CCl_4 injury experiments, CCl_4 was injected into mice intraperitoneally at the dose of 2 mL/kg body weight, twice per week for 4 weeks (Tu et al., 2015). BrdU (Sigma-Aldrich, St. Louis, MO) was injected at the dose of 50 mg/kg 2 h before sacrifice (Zhang et al., 2015). Tissue and serum were collected at the end of the experiments. All procedures followed the Huazhong Agricultural University Guidelines for the Care and Use of Laboratory Animals.

Cell Culture and Transient Transfection

The cell lines used in this study included HepG2, HIBEpic, LO2, BMSC, HeLa, and C3H10 cells. All cells were seeded into 6- or 24-well plates, and grown in high glucose DMEM (HyClone, Logan, UT) supplied with 10% (v/v) fetal bovine serum (Gibco BRL, Grand Island, NY) and 1% (v/v) penicillin-streptomycin. The following day, cells were transfected with plasmid, mimics, or siRNA. Transient transfection was performed using lipofectamine 2000 or lipofectamine RNAiMAX (Invitrogen, Carlsbad, CA). The primer sequences are listed in Table S1.

Immunofluorescent Analysis

Liver tissues were immobilized with 4% paraformaldehyde (PFA), dehydrated, embedded in paraffin, sectioned at 5 μm, and processed for immunofluorescent staining. SOX9 antibody (AB5535,

Millipore, USA) and HNF4α antibody (Ab41898, Abcam, Cambridge, England) were used to incubate sections at 4°C overnight. Slides were washed with PBS and incubated with corresponding secondary antibodies for 1 h, followed by PBS washes. The incubated slides were added with DAPI for nuclear staining and mounting. Images were acquired with a laser scanning confocal microscope (LSM710, Carl Zeiss Microscopy), were analyzed by Zen software with fixed parameters.

Liver Histology and Immunohistochemical Staining

Liver tissues were immobilized with 4% PFA, dehydrated, embedded in paraffin, sectioned at 5 μm, and processed for H&E, Masson trichrome, and Sirius red staining.

In Situ mRNA Hybridization

In situ detection of *Sox9* and *Hnf4α* RNA transcripts was carried out on OCT-embedded tissue sections using the RNAscope Multiplex Fluorescent Reagent Kit v.2 (Advanced Cell Diagnostics). RNAscope probes for *Sox9*, *Hnf4α*, standard negative probe for 4-hydroxy-tetrahydrodipicolinate reductase, and positive probe for peptidylprolyl isomerase B were used for *in situ* detection. The probe information for RNAscope assay is listed in Table S2.

RNA Isolation and qRT-PCR

The RNAiso Plus (Takara, Japan) was used to isolate total RNA, including low-molecular-weight RNA from frozen samples and cell lines, according to the manufacturer's protocol. Then the first-strand cDNA was synthesized using the PrimeScript RT Reagent Kit with gDNA Eraser (Takara). Real-time PCR was performed using the MonAmp SYBR Green qPCR Mix (Low ROX). The relative levels were calculated using the comparative-Ct method ($2^{-\Delta\Delta Ct}$ method). The primer sequences are listed in Table S3.

Western Blots

For whole-cell protein extraction, liver tissues were prepared in lysis buffer (Beyotime, Jiangsu, China) according to the manufacturer's instructions. Protein lysates were separated by SDS-PAGE. Next, the gel was transferred to polyvinylidene difluoride membranes (Millipore). After being blocked with 5% skimmed milk, the membranes were incubated overnight with the anti-SOX9 (AB5535, Millipore), anti-HOXB6 (sc-166950X, Santa Cruz Biotechnology), anti-GAPDH (60004-I-Ig, Proteintech, Chicago), or Tag-3*Flag-antibody (66008-3-Ig, Proteintech) at 4°C. Then, the membranes were incubated with the corresponding horseradish peroxidase-conjugated secondary antibodies at room temperature for 1.5 h. Finally, the membranes were visualized with enhanced chemiluminescence (Bio-Rad, USA).

Biochemical Evaluation

Plasma was collected from blood after centrifugation (at 3,000 rpm) for 10 min at 4°C. Plasma ALT, AST, and LDH were determined to evaluate liver injury using a Multiskan MK3 microplate reader (Thermo Electron Corporation, USA) and commercial kits (Nanjing Jiancheng Bioengineering Institute, China), according to the manufacturer's instructions.

Lentivirus, Adenovirus, and AAV Plasmid Construction and Production

Two pairs of CRISPR guide RNAs (gRNAs) targeting *pre-miR-126* gene were initially screened in NIH3T3cells. The gRNA targeting sgRNA3 displayed ~50% mutagenesis at the on-target site in *pre-miR-126*, as determined by PCR and a T7EN1 cleavage assay (Yan et al., 2019). After targeting efficiency was confirmed, the sgRNAs were constructed into lentivirus and adenovirus plasmids. To completely overexpress HOXB6 *in vivo*, the *Hoxb6*-CDS sequence was inserted into the multiple cloning site in the pHBAAV-CMV-*Hoxb6*-3flag-T2A-ZsGreen plasmid. The two recombinant AAV plasmids were transfected into HEK293 cells with pAAV-RC and pHelper by using lipofectamine 2000 transfection reagent (Invitrogen, Carlsbad, CA). AAV-*Hoxb6* and AAV-ctrl (Hanbio, Shanghai, China) were packaged in AAV-293 cells.

Intravenous Virus Injection for Liver Transduction

To intravenously inject adenovirus or AAVs, mice were restrained in a rodent restrainer, their tails were dilated using a heat lamp or warm water and sterilized by 70% ethanol, and 100 μL of concentrated AAV (1×10^{10} particles per mouse) was injected into the tail vein of each mouse.

ChIP

ChIP assays were performed using the ChIP Assay kit (Beyotime) according to the user manual. In brief, HepG2 cells were transfected with pEGFP-N1-*HOXB6* for 24 h, and then incubated with formaldehyde at a final concentration of 1% (v/v) for 10 min at 37°C to crosslink the nuclear proteins to DNA. Subsequently, cells were sonicated and then immunoprecipitated with the antibody against HOXB6 (sc-166950, Santa Cruz Biotechnology), taking IgG as a negative control, or without anti-HOXB6 and IgG in the reaction as mock control. The captured chromatin was eluted and un-crosslinked, and the DNA was recovered. The ChIP-isolated DNA was subjected to PCR amplification using the primer pair spanning the *SOX9*-P6-3 in the *SOX9* promoter region. The primer sequences are listed in Table S4.

Statistical Methods

Data are expressed as the means ± SD and were analyzed using Prism 6 (GraphPad). Statistical details of the experiments can be found in the Results and figure legends. Student's two-tailed t test (unpaired) was used to determine statistical significance differences between groups. Statistical significance was presented at the level of *$p < 0.05$, **$p < 0.01$, ***$p < 0.001$.

Data and Code Availability

The sequences of all primers are included in the paper.

SUPPLEMENTAL INFORMATION

Supplemental Information can be found online at https://doi.org/10.1016/j.stemcr.2020.07.005.

AUTHORS CONTRIBUTIONS

Y.Y. and Lisheng Zhang conceived and designed the study. Y.Y. provided the experimental data. Y.Y. and R.W. performed the cell and animal experiments. X.H., S.W., Liang Zhang, and C.H. provided assistance in molecular assays. Y.Y. and R.W. discussed and drafted the manuscript. Lisheng Zhang organized the data and wrote the manuscript.

ACKNOWLEDGMENTS

This work was supported by National Key R&D plan nos. 2017YFA0103202 and 2017YFA0103200, the Fundamental Research Funds for the Central Universities (2662017PY106, 2662016PY087, and 2662019YJ008), and HZAU Startup funds to L.Z.

Received: January 14, 2020
Revised: July 7, 2020
Accepted: July 8, 2020
Published: August 6, 2020

REFERENCES

Alison, M.R., and Lin, W.R. (2011). Hepatocyte turnover and regeneration: virtually a virtuoso performance. Hepatology *53*, 1393–1396.

Amsellem, S., Pflumio, F., Bardinet, D., Izac, B., Charneau, P., Romeo, P.H., Dubart-Kupperschmitt, A., and Fichelson, S. (2003). Ex vivo expansion of human hematopoietic stem cells by direct delivery of the HOXB4 homeoprotein. Nat. Med. *9*, 1423–1427.

Bartel, D.P. (2018). Metazoan MicroRNAs. Cell *173*, 20–51.

Bhatlekar, S., Fields, J.Z., and Boman, B.M. (2018). Role of HOX genes in stem cell differentiation and cancer. Stem Cells Int. *2018*, 3569493.

Catron, K.M., Iler, N., and Abate, C. (1993). Nucleotides flanking a conserved TAAT core dictate the DNA binding specificity of three murine homeodomain proteins. Mol. Cell. Biol. *13*, 2354–2365.

Fischbach, N.A., Rozenfeld, S., Shen, W., Fong, S., Chrobak, D., Ginzinger, D., Kogan, S.C., Radhakrishnan, A., Le Beau, M.M., Largman, C., et al. (2005). HOXB6 overexpression in murine bone marrow immortalizes a myelomonocytic precursor in vitro and causes hematopoietic stem cell expansion and acute myeloid leukemia in vivo. Blood *105*, 1456–1466.

Font-Burgada, J., Shalapour, S., Ramaswamy, S., Hsueh, B., Rossell, D., Umemura, A., Taniguchi, K., Nakagawa, H., Valasek, M.A., Ye, L., et al. (2015). Hybrid periportal hepatocytes regenerate the injured liver without giving rise to cancer. Cell *162*, 766–779.

Furuyama, K., Kawaguchi, Y., Akiyama, H., Horiguchi, M., Kodama, S., Kuhara, T., Hosokawa, S., Elbahrawy, A., Soeda, T., Koizumi, M., et al. (2011). Continuous cell supply from a Sox9-expressing progenitor zone in adult liver, exocrine pancreas and intestine. Nat. Genet. *43*, 34–41.

Gailhouste, L., Gomez-Santos, L., Hagiwara, K., Hatada, I., Kitagawa, N., Kawaharada, K., Thirion, M., Kosaka, N., Takahashi, R.U., Shibata, T., et al. (2013). miR-148a plays a pivotal role in the liver by promoting the hepatospecific phenotype and suppressing the invasiveness of transformed cells. Hepatology *58*, 1153–1165.

Graba, Y., Aragnol, D., and Pradel, J. (1997). *Drosophila* Hox complex downstream targets and the function of homeotic genes. BioEssays *19*, 379–388.

Halpern, K.B., Shenhav, R., Matcovitch-Natan, O., Toth, B., Lemze, D., Golan, M., Massasa, E.E., Baydatch, S., Landen, S., Moor, A.E., et al. (2017). Single-cell spatial reconstruction reveals global division of labour in the mammalian liver. Nature *542*, 352–356.

Han, X., Wang, Y., Pu, W., Huang, X., Qiu, L., Li, Y., Yu, W., Zhao, H., Liu, X., He, L., et al. (2019). Lineage tracing reveals the bipotency of $SOX9^{+}$ hepatocytes during liver regeneration. Stem Cell Reports *12*, 624–638.

Huch, M., Dorrell, C., Boj, S.F., van Es, J.H., Li, V.S., van de Wetering, M., Sato, T., Hamer, K., Sasaki, N., Finegold, M.J., et al. (2013). In vitro expansion of single $Lgr5^{+}$ liver stem cells induced by Wnt-driven regeneration. Nature *494*, 247–250.

Itoh, T., and Miyajima, A. (2014). Liver regeneration by stem/progenitor cells. Hepatology *59*, 1617–1626.

John, K., Hadem, J., Krech, T., Wahl, K., Manns, M.P., Dooley, S., Batkai, S., Thum, T., Schulze-Osthoff, K., and Bantel, H. (2014). MicroRNAs play a role in spontaneous recovery from acute liver failure. Hepatology *60*, 1346–1355.

Kappen, C. (2000). Disruption of the homeobox gene Hoxb-6 in mice results in increased numbers of early erythrocyte progenitors. Am. J. Hematol. *65*, 111–118.

Kitade, M., Factor, V.M., Andersen, J.B., Tomokuni, A., Kaji, K., Akita, H., Holczbauer, A., Seo, D., Marquardt, J.U., Conner, E.A., et al. (2013). Specific fate decisions in adult hepatic progenitor cells driven by MET and EGFR signaling. Genes Dev. *27*, 1706–1717.

Ko, S., Russell, J.O., Tian, J., Gao, C., Kobayashi, M., Feng, R., Yuan, X., Shao, C., Ding, H., Poddar, M., et al. (2019). Hdac1 regulates differentiation of bipotent liver progenitor cells during regeneration via Sox9b and Cdk8. Gastroenterology *156*, 187–202.e14.

Krumlauf, R. (1994). Hox genes in vertebrate development. Cell *78*, 191–201.

Larsen, B.M., Hrycaj, S.M., Newman, M., Li, Y., and Wellik, D.M. (2015). Mesenchymal Hox6 function is required for mouse pancreatic endocrine cell differentiation. Development *142*, 3859–3868.

Laudadio, I., Manfroid, I., Achouri, Y., Schmidt, D., Wilson, M.D., Cordi, S., Thorrez, L., Knoops, L., Jacquemin, P., Schuit, F., et al. (2012). A feedback loop between the liver-enriched transcription factor network and miR-122 controls hepatocyte differentiation. Gastroenterology *142*, 119–129.

Lechman, E.R., Gentner, B., van Galen, P., Giustacchini, A., Saini, M., Boccalatte, F.E., Hiramatsu, H., Restuccia, U., Bachi, A., Voisin, V., et al. (2012). Attenuation of miR-126 activity expands HSC in vivo without exhaustion. Cell Stem Cell *11*, 799–811.

Li, D., Li, W., and Hui, L. (2016). Hybrid hepatocyte: a newly identified player for regeneration in hepatic injuries. Hepatology *64*, 2244–2246.

Li, H., Cai, H., Deng, J., Tu, X., Sun, Y., Huang, Z., Ding, Z., Dong, L., Chen, J., Zang, Y., et al. (2018). TGF-beta-mediated upregulation of Sox9 in fibroblast promotes renal fibrosis. Biochim. Biophys. Acta *1864*, 520–532.

Luna, J.M., Barajas, J.M., Teng, K.Y., Sun, H.L., Moore, M.J., Rice, C.M., Darnell, R.B., and Ghoshal, K. (2017). Argonaute CLIP defines a deregulated miR-122-bound transcriptome that correlates with patient survival in human liver cancer. Mol. Cell *67*, 400–410.e7.

Mori-Akiyama, Y., van den Born, M., van Es, J.H., Hamilton, S.R., Adams, H.P., Zhang, J., Clevers, H., and de Crombrugghe, B. (2007). SOX9 is required for the differentiation of paneth cells in the intestinal epithelium. Gastroenterology *133*, 539–546.

Mori, M.A., Ludwig, R.G., Garcia-Martin, R., Brandão, B.B., and Kahn, C.R. (2019). Extracellular miRNAs: from biomarkers to mediators of physiology and disease. Cell Metab. *30*, 656–673.

Parent, R., and Beretta, L. (2008). Translational control plays a prominent role in the hepatocytic differentiation of HepaRG liver progenitor cells. Genome Biol. *9*, R19.

Porcelli, D., Fischer, B., Russell, S., and White, R. (2019). Chromatin accessibility plays a key role in selective targeting of Hox proteins. Genome Biol. *20*, 115.

Pu, W., Zhang, H., Huang, X., Tian, X., He, L., Wang, Y., Zhang, L., Liu, Q., Li, Y., Li, Y., et al. (2016). Mfsd2a^{+} hepatocytes repopulate the liver during injury and regeneration. Nat. Commun. *7*, 13369.

Rocha, A.S., Vidal, V., Mertz, M., Kendall, T.J., Charlet, A., Okamoto, H., and Schedl, A. (2015). The angiocrine factor Rspondin3 is a key determinant of liver zonation. Cell Rep. *13*, 1757–1764.

Rogler, C.E., Levoci, L., Ader, T., Massimi, A., Tchaikovskaya, T., Norel, R., and Rogler, L.E. (2009). MicroRNA-23b cluster microRNAs regulate transforming growth factor-beta/bone morphogenetic protein signaling and liver stem cell differentiation by targeting Smads. Hepatology *50*, 575–584.

Sánchez-Higueras, C., and Rastogi, C. (2019). In vivo Hox binding specificity revealed by systematic changes to a single cis regulatory module. Nat. Commun. *10*, 3597.

Shen, W., Chrobak, D., Krishnan, K., Lawrence, H.J., and Largman, C. (2004). HOXB6 protein is bound to CREB-binding protein and represses globin expression in a DNA binding-dependent, PBX interaction-independent process. J. Biol. Chem. *279*, 39895–39904.

Shen, W.F., Krishnan, K., Lawrence, H.J., and Largman, C. (2001). The HOX homeodomain proteins block CBP histone acetyltransferase activity. Mol. Cell Biol. *21*, 7509–7522.

Su, Q., Kumar, V., Sud, N., and Mahato, R.I. (2018). MicroRNAs in the pathogenesis and treatment of progressive liver injury in NAFLD and liver fibrosis. Adv. Drug Deliv. Rev. *129*, 54–63.

Suzuki, A., Zheng, Y., Kondo, R., Kusakabe, M., Takada, Y., Fukao, K., Nakauchi, H., and Taniguchi, H. (2000). Flow-cytometric separation and enrichment of hepatic progenitor cells in the developing mouse liver. Hepatology *32*, 1230–1239.

Tarlow, B.D., Finegold, M.J., and Grompe, M. (2014). Clonal tracing of Sox9+ liver progenitors in mouse oval cell injury. Hepatology *60*, 278–289.

Thorsteinsdottir, U., Sauvageau, G., Hough, M.R., Dragowska, W., Lansdorp, P.M., Lawrence, H.J., Largman, C., and Humphries, R.K. (1997). Overexpression of HOXA10 in murine hematopoietic cells perturbs both myeloid and lymphoid differentiation and leads to acute myeloid leukemia. Mol. Cell Biol. *17*, 495–505.

Tsay, H.C., Yuan, Q., Balakrishnan, A., Kaiser, M., Mobus, S., Kozdrowska, E., Farid, M., Tegtmeyer, P.K., Borst, K., Vondran, F.W.R., et al. (2019). Hepatocyte-specific suppression of microRNA-221-3p mitigates liver fibrosis. J. Hepatol. *70*, 722–734.

Tu, X., Zheng, X., Li, H., Cao, Z., Chang, H., Luan, S., Zhu, J., Chen, J., Zang, Y., and Zhang, J. (2015). MicroRNA-30 protects against carbon tetrachloride-induced liver fibrosis by attenuating transforming growth factor beta signaling in hepatic stellate cells. Toxicol. Sci. *146*, 157–169.

Wang, B., Zhao, L., Fish, M., Logan, C.Y., and Nusse, R. (2015). Self-renewing diploid Axin2^{+} cells fuel homeostatic renewal of the liver. Nature *524*, 180–185.

Wei, W., Hou, J., Alder, O., Ye, X., Lee, S., Cullum, R., Chu, A., Zhao, Y., Warner, S.M., Knight, D.A., et al. (2013). Genome-wide microRNA and messenger RNA profiling in rodent liver development implicates mir302b and mir20a in repressing transforming growth factor-beta signaling. Hepatology *57*, 2491–2501.

Yan, Y., Qin, D., Hu, B., Zhang, C., Liu, S., Wu, D., Huang, W., Huang, X., Wang, L., Chen, X., et al. (2019). Deletion of miR-126a promotes hepatic aging and inflammation in a mouse model of cholestasis. Mol. Ther. Nucleic Acids *16*, 494–504.

Yan, Y., Wang, S., Wang, R., Jiang, P., Chen, Y., Zhang, L., Hou, C., and Zhang, L. (2020). Transcriptional regulation of microRNA-126a by farnesoid X receptor in vitro and in vivo. Biotechnol. Lett. *27*, 2020. https://doi.org/10.1007/s10529-020-02864-7.

Yoon, B.S., Ovchinnikov, D.A., Yoshii, I., Mishina, Y., Behringer, R.R., and Lyons, K.M. (2005). Bmpr1a and Bmpr1b have overlapping functions and are essential for chondrogenesis in vivo. Proc. Natl. Acad. Sci. U S A *102*, 5062–5067.

Zhang, B., Nguyen, L.X.T., Li, L., Zhao, D., Kumar, B., Wu, H., Lin, A., Pellicano, F., and Hopcroft, L. (2018). Bone marrow niche trafficking of miR-126 controls the self-renewal of leukemia stem cells in chronic myelogenous leukemia. Nat. Med. *24*, 450–462.

Zhang, L., Yang, L., Liu, X., Chen, W., Chang, L., Chen, L., Loera, S., Chu, P., Huang, W.C., Liu, Y.R., et al. (2013). MicroRNA-657 promotes tumorigenesis in hepatocellular carcinoma by targeting transducin-like enhancer protein 1 through nuclear factor kappa B pathways. Hepatology *57*, 1919–1930.

Zhang, R.R., Zheng, Y.W., Li, B., Tsuchida, T., Ueno, Y., Nie, Y.Z., and Taniguchi, H. (2015). Human hepatic stem cells transplanted into a fulminant hepatic failure Alb-TRECK/SCID mouse model exhibit liver reconstitution and drug metabolism capabilities. Stem Cell Res. Ther. *6*, 49.

Stem Cell Reports, Volume *15*

Supplemental Information

MiR-126 Regulates Properties of SOX9^{+} Liver Progenitor Cells during Liver Repair by Targeting *Hoxb6*

Yi Yan, Rui Wang, Xiongji Hu, Shichao Wang, Liang Zhang, Chenjiao Hou, and Lisheng Zhang

Supplemental Information
This PDF file includes:
Figures S1 to S5
Tables S1 to S4
Supplemental Experimental Procedures
Supplemental References

Supplemental Figures

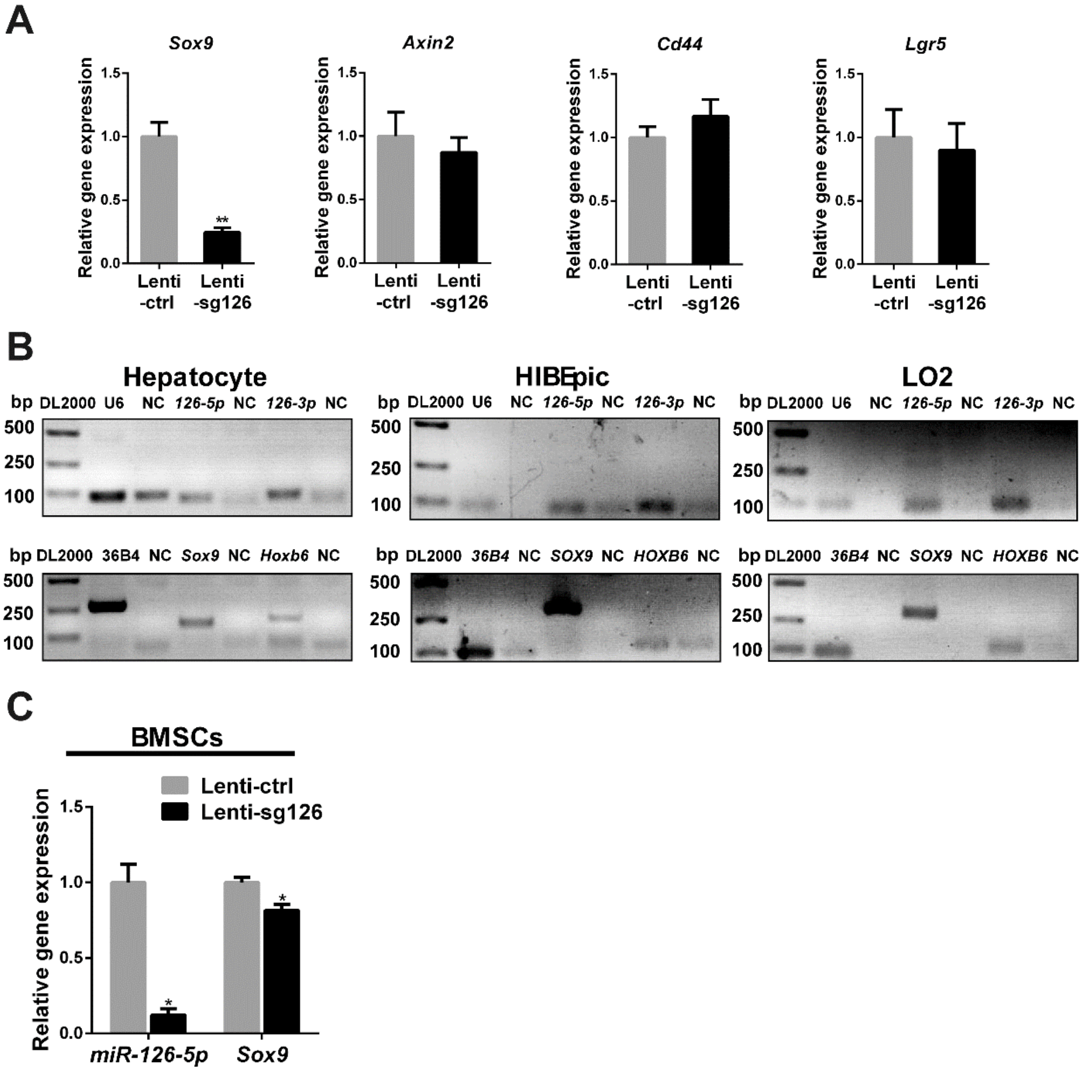

Figure S1. ***miR-126*** **controls stem cell-specific gene** ***Sox9*** **expression** ***in vitro*** **and** ***in vivo*****, related to Figure 1.**

(A) C3H10 cells were infected with Lentivirus-control (Lenti-ctrl) or Lentivirus-sg126 (Lenti-sg126) to obtain stable cell lines. Quantification of *Sox9*, *Axin2*, *Cd44* and *Lgr5* expression in Lenti-ctrl C3H10 and Lenti-sg126 C3H10.

(B) PCR expression analysis of mature *miR-126-3p*, *miR-126-5p*, *HOXB6*, and *SOX9* in mouse primary hepatocytes (left), HIBEpic cells (middle) and LO2 (right).

(C) BMSCs were infected with Lentivirus-control (Lenti-ctrl) or Lentivirus-sg126 (Lenti-sg126) to obtain stable cell lines. Quantification of *miR-126-5p* and *Sox9* expression in Lenti-ctrl BMSCs and Lenti-sg126 BMSCs.

Data are expressed as means ± SD, n = 3 independent experiments containing three replicates. Significant difference is presented at the levels of $*p < 0.05$, $**p < 0.01$ by two-tailed Student's t-test.

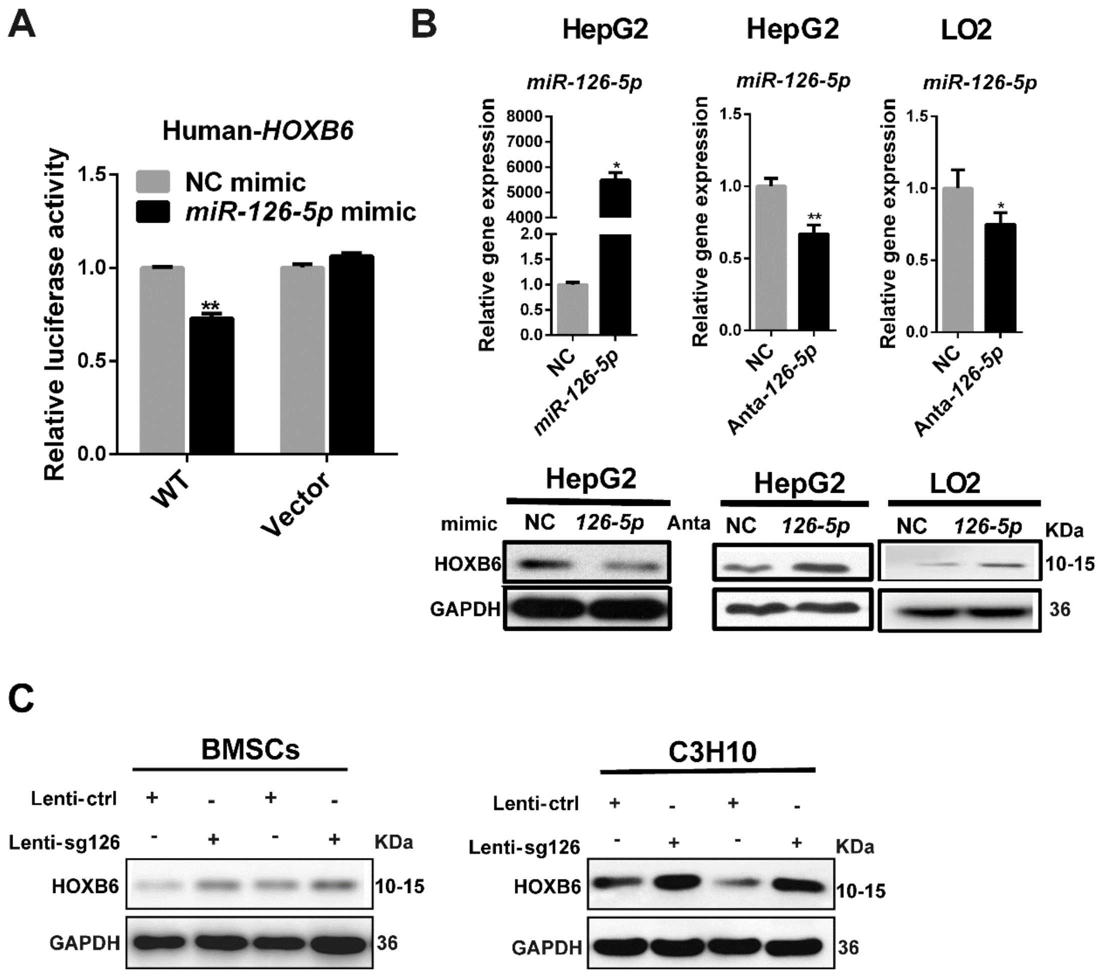

Figure S2. ***Hoxb6*** **is a target of** ***miR-126-5p*****, related to Figure 2.**

(A) Relative luciferase activity assays of luciferase reporters with human *HOXB6* 3 ′ UTR and psiCHECK2 were performed after co-transfection with *miR-126-5p* mimics or NC mimics in Hela cells.

(B) HepG2 cells or LO2 cells were transiently transfected with *miR-126-5p* mimics, antago*miR-126-5p* (Anta-126-5p) or stable negative control (NC). The mRNA levels of *miR-126-5p* were detected by qRT-PCR. The protein levels of HOXB6 were detected by western blot.

(C) BMSCs and C3H10 cells were infected with Lentivirus-control (Lenti-ctrl) or Lentivirus-sg126 (Lenti-sg126) to obtain stable cell lines. Western blotting analysis was performed to detect HOXB6 after deletion of *miR-126* in BMSCs and C3H10 cells.

Data are expressed as means ± SD, n = 3 independent experiments containing three replicates. Significant difference is presented at the levels of $^*p < 0.05$, $^{**}p < 0.01$ by two-tailed Student's t-test.

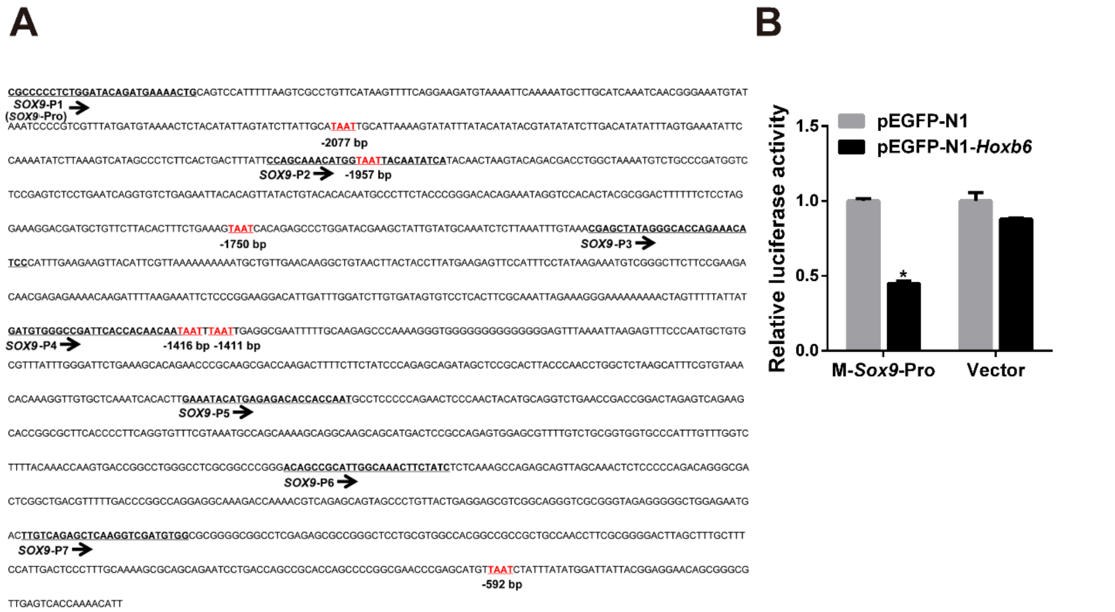

Figure S3. HOXB6 negatively regulates *SOX9* trans-activity, related to Figure 4.

(A) *SOX9*-Pro (1700bp) promoter sequence containing six putative HOXB6-binding sites (TAAT, red): I (-2077bp), II (-1957bp), III (-1750bp), IV (-1416bp), V (-1411bp), and VI (-592bp). The promoter truncated fragments were amplified by PCR. The primers were underlined.

(B) Relative luciferase activity analysis of the *Sox9* promoter–reporter constructs. A full-long fragment of the mouse *Sox9* promoter was co-transfected into HEK-293T cells with the phRL-TK and pEGFP-N1, or pEGFP-N1-*Hoxb6* eukaryotic expression plasmids. Forty-eight hours post transfection, the cell extracts were prepared and analyzed for luciferase activity.

Data are expressed as means ± SD, n = 3 independent experiments containing three replicates. Significant difference is presented at the levels of $*p < 0.05$ by two-tailed Student's t-test.

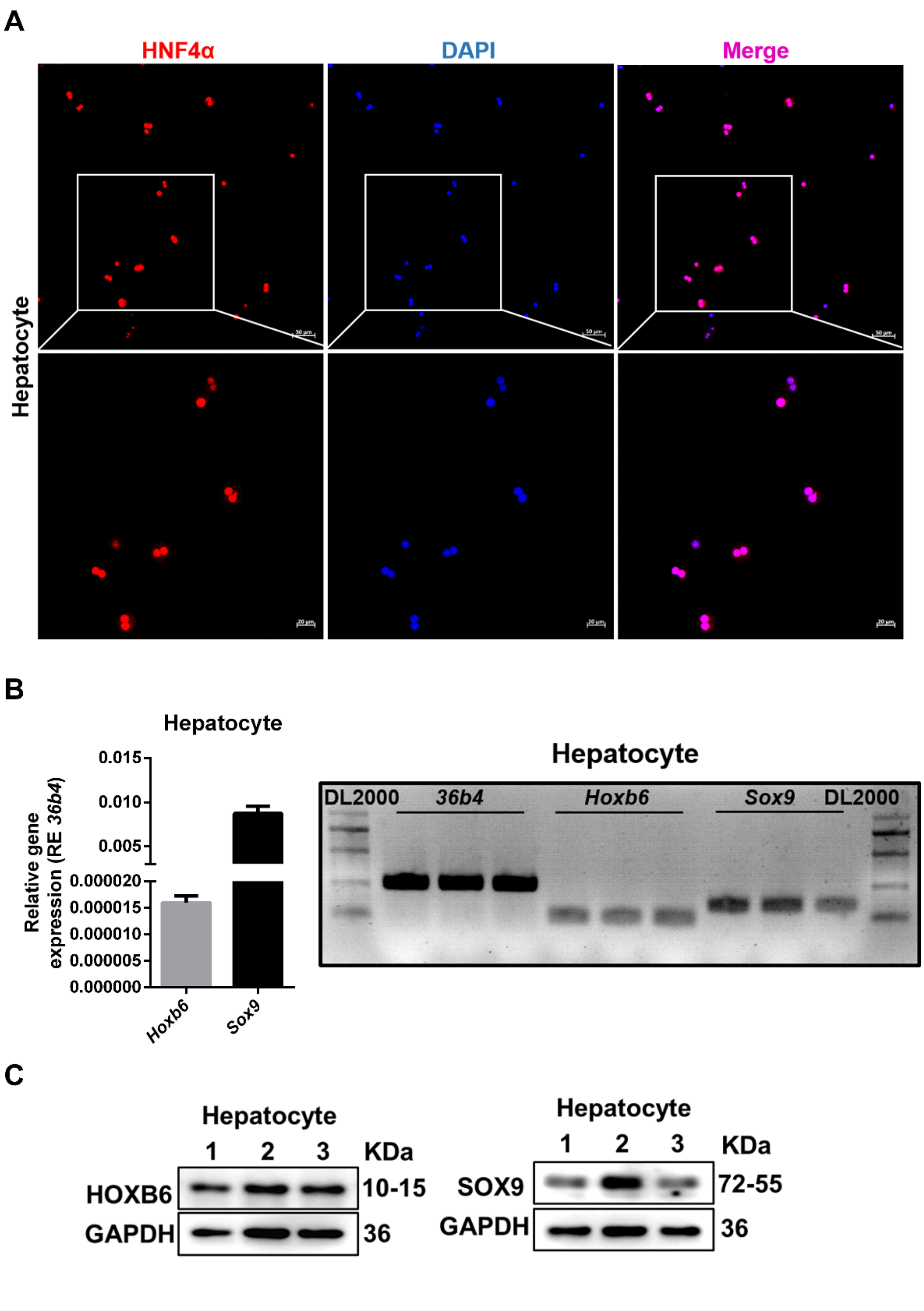

Figure S4. The expression of HOXB6 and SOX9 in primary hepatocyte, related to Figure 5. Primary hepatocytes were isolated from normal fresh liver material obtained.

(A) Immunofluorescence micrographs of typical primary hepatocyte. hepatocyte sections labelled with antibodies against HNF4α. Blue (DAPI) shows nuclei. Red (HNF4α) marks hepatocytes. Scale bar represents 20μm.

(B) Normal mouse primary hepatocytes expression levels of *Hoxb6* and *Sox9* were measured by qRT-PCR and PCR.

(C) Normal mouse primary hepatocytes expression levels of HOXB6 and SOX9 were measured by western blot.

Data are expressed as means ± SD, n = 3 mice per group containing three replicates.

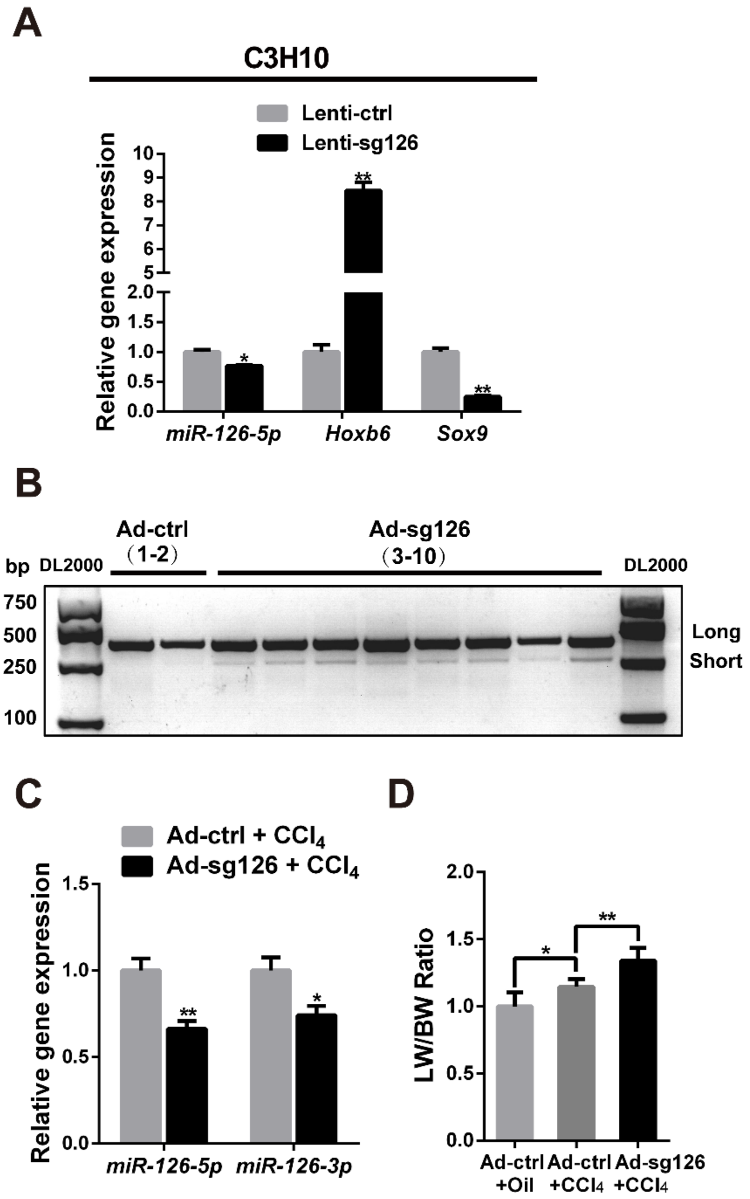

Figure S5. Deletion of *miR-126* suppresses functions of SOX9$^+$ LPCs by targeting *Hoxb6*, related to Figure 6.

(A) C3H10 cells were infected with Lentivirus-control (Lenti-ctrl) or Lentivirus-sg126 (Lenti-sg126) to obtain stable cell lines. Quantification of *miR-126-5p*, *Hoxb6*, and *Sox9* expression in Lenti-ctrl C3H10 and Lenti-sg126 C3H10.

(B) PCR cleavage assays were performed with genomic DNA from liver samples from mice at day 7 post infection with Ad.ctrl (lanes 1–2) and Ad.sg126 (lanes 3–10).

(C) Hepatic expression levels of *miR-126-3p* and *miR-126-5p* genes were measured in Ad-ctrl-treated or Ad-sg126-treated mice after CCl_4 injury by qRT-PCR.

(D) Effect of *miR-126* deletion on the LW/BW ratio after CCl_4 injury.

Data are expressed as means ± SD, n = 3 independent experiments containing three replicates (A), n = 6 mice per group containing three replicates (C and D). Significant difference is presented at the levels of *$p < 0.05$, **$p < 0.01$ by two-tailed Student's t-test.

Supplemental Tables

Table S1. Oligonucleotide Sequences for *miR-126a* and *Hoxb6* siRNA in this study, Related to Figure 1, Figure 2, Figure 3, and Figure 6.

Gene name	Primer sequence (5'-3')
miR-126-5p mimics sense	CAUUAUUACUUUUGGUACGCG
miR-126-5p mimics antisense	CGUACCAAAAGUAAUAAUGUU
M-*Hoxb6*-siRNA sense	CCACUGGAAGGAGCACAUATT
M-*Hoxb6*-siRNA antisense	UAUGUGCUCCUUCCAGUGGTT
Negative control sense	UUCUCCGAACGUGUCACGUTT
Negative control antisense	ACGUGACACGUUCGGAGAATT
miR-126a-5p-antagomir	CGCGUACCAAAAGUAAUAAUG

Table S2. Probe information for RNAscope® assay in this study, Related to Figure 1 and Figure 5.

Official symbol	Cat No.	Channel	Detection Kit
Sox9	401051	C3	RNAscope® Multiplex Fluorescent Reagent Kit v2
Hnf4α	497651	C2	
3-Plex Positive Control Probe-Mm	320881	C3	
3-Plex Negative Control Probe (*Dapb*)	320871	C3	

Table S3. Primer sequences for reverse transcription PCR and qRT-PCR in this study, Related to Figure 1, Figure 3, and Figure 5.

Gene name	Primer sequence (5'-3')
RT-loop-*miR126-5p*	GTCGTATCCAGTGCAGGGTCCGAGGTATTCGCACTGGATAC GACCGCGTA
RT-loop-*miR126-3p*	GTCGTATCCAGTGCAGGGTCCGAGGTATTCGCACTGGATAC GACCGCATT
miR-126a-5p-F	GGCGACGGCATTATTACTTTTG
miR-126a-3p-F	AAGGTTGTTCGTACCGTGAGT
UR	CAGTGCAGGGTCCGAGGT
U6-F	GCTTCGGCAGCACATATACTAA
U6-R	CGAATTTGCGTGTCATCCTT
H-*GAPDH*-RT-F	CTCTGGTAAAGTGGATATTG
H-*GAPDH*-RT-R	GGTGGAATCATATTGGAAC
H-*SOX9*-RT-F	TCCTCAGGCTTTGCGATTT
H-*SOX9*-RT-R	TGCTCGGGCACTTATTGG
H-*HOXB6*-RT-F	CTACCGCGAGAAAGAGTCGG
H-*HOXB6*-RT-R	TGTTGCACGAATTCATCCGC
M-*36b4*-RT-F	TGGAGACAAGGTGGGAGCC
M-*36b4*-RT-R	CACAGACAATGCCAGGACGC
M-*Sox9*-RT-F	TGCAAGCTGGCAAAGTTGAT
M-*Sox9*-RT-R	TCAGTTCACCGATGTCCACG
M-*Hoxb6*-RT-F	CCTACACACGCTACCAGACC
M-*Hoxb6*-RT-R	TGAGACGCACTGAGCAGTTT
M-*Cyp7a1*-RT-F	GGGGATTGCTGTGGTAG
M-*Cyp7a1*-RT-R	CAGGGAGTTTGTGATGAAG
M-*E-cad*-RT-F	TGATGATGCCCCCAACACTC
M-*E-cad*-RT-R	CCAAGCCCTTGGCTGTTTTC

Table S4. Primer sequences for luciferase reporter assays and ChIP assays in this study, Related to Figure 2 and Figure 4.

Gene name	Primer sequence (5'-3')
m-*Hoxb6*-3UTR -F	GCGCTCGAGTGAGAATCGGACTCACTTGAT
m-*Hoxb6*-3UTR-R	GCGCGCGGCCGCTTGCGAGTTTTCACATCTTTA
m-*Hoxb6*-3UTR-mut1-F	GATTATGATGATGATGATAAGCCGCCTAAAGATGTGAAA ACTCG
m-*Hoxb6*-3UTR-mut1-R	CGAGTTTTCACATCTTTAGGCGGCTTATCATCATCATCAT AATC
m-*Hoxb6*-3UTR-mut2-F	GGTAGCGCTATTAATATTATCGGCGCGGATTATGATGAT GATGATAAGCCGCCTAAAG
m-*Hoxb6*-3UTR-mut2-R	CCGCGCCGATAATATTAATAGCGCTACCGAAGTATTTCA CGTCCGGAGCTAAGACAAG
H-*SOX9*-Pro-full-long-F	GGGGTACCCGCCCCCTCTGGATACAGATGAAAACTG
H-*SOX9*-Pro-full-long-R	CCCAAGCTTAATGTTTTGGTGACTCAACGCCCGCTG
H-*SOX9*-P1-F	GGGGTACCCGCCCCCTCTGGATACAGATGAAAACTG
H-*SOX9*-P1-R	CCCAAGCTTGAGACTCGGAGACCATCGGGCAGAC
H-*SOX9*-P2-F	GGGGTACCCCAGCAAACATGGTAATTACAATATCA
H-*SOX9*-P2-R	CCCAAGCTTGGATGTTTCTGGTGCCCTATAGC
H-*SOX9*-P3-F	GGGGTACCCGAGCTATAGGGCACCAGAAACATCC
H-*SOX9*-P3-R	CCCAAGCTTAATTAAATTATTGTTGTGGTGAATCGGCC
H-*SOX9*-P4-F	GGGGTACCGATGTGGGCCGATTCACCACAACAAT
H-*SOX9*-P4-R	CCCAAGCTTCGGTTCAGACCTGCATGTAGTTGGGAG
H-*SOX9*-P5-F	GGGGTACCGAAATACATGAGAGACACCACCAAT
H-*SOX9*-P5-R	CCCAAGCTTGTTTGCTAACTGCTCTGGCTTTG
H-*SOX9*-P6-F	GGGGTACCACAGCCGCATTGGCAAACTTCTATC
H-*SOX9*-P6-R	CCCAAGCTTGAAAGCAAAGCTAAGTCCCCGCG
H-*SOX9*-P7-F	GGGGTACCTTGTCAGAGCTCAAGGTCGATGTGG
H-*SOX9*-P7-R	CCCAAGCTTAATGTTTTGGTGACTCAACGCCC
H-*SOX9*-P6-1-F	GGGGTACCACAGCCGCATTGGCAAACTTCTATC
H-*SOX9*-P6-1-R	CCCAAGCTTGCTCTGACGTTTTGGTCTTTGCCT
H-*SOX9*-P6-2-F	GGGGTACCGTTTTTGACCCGGCCAGG
H-*SOX9*-P6-2-R	CCCAAGCTTCACATCGACCTTGAGCTCTGAC
H-*SOX9*-P6-3-F	GGGGTACCGAGGGGGCTGGAGAATGACTT
H-*SOX9*-P6-3-R	CCCAAGCTTGAAAGCAAAGCTAAGTCCCCG
H-*SOX9*-P6-3-1--F	GGGGTACCGAGGGGGCTGGAGAATGACTT
H-*SOX9*-P6-3-1--R	CCCAAGCTTCCGCCCCGCGCCA
H-*SOX9*-P6-3-2--F	GGGGTACCGGTCGATGTGGCGCGG
H-*SOX9*-P6-3-2--R	CCCAAGCTTCCGTGGCCACGCAGGAG
H-*SOX9*-P6-3-3--F	GGGGTACCGCTCCTGCGTGGCCACG
H-*SOX9*-P6-3-3--R	CCCAAGCTTGAAAGCAAAGCTAAGTCCCCG
H-*SOX9*-P6-3-ChIP-F	CTGGAGAATGACTTGTCAGAGC
H-*SOX9*-P6-3-ChIP-R	AAAGCTAAGTCCCCGCGAA

H: Human M: Mouse F: Forward R: Reverse

Supplemental Experimental Procedures

Mice and Injury Regimens

Adult C57BL/6J male mice (8 weeks old) were housed in a pathogen-free animal facility under a standard 12 hours light/dark cycle. Mice were given standard rodent chow and water ad libitum. For CCl_4 injury experiments, CCl_4 was diluted with paraffin oil to achieve a final concentration of 20%, and the diluted CCl_4 was injected into mice intraperitoneal at the dose of 2 ml/kg body weight, twice per week for four weeks. Control mice were provided with an equal volume of oil vehicle. Bromodeoxyuridine (BrdU) (Sigma-Aldrich, St. Louis, MO) was injected at the dose of 50 mg/kg 2 hours before sacrifice. After mice were killed, liver tissues were frozen in liquid nitrogen or fixed in 10% buffered formalin and embedded in paraffin and serum were collected. All procedures followed the Huazhong Agricultural University Guidelines for the Care and Use of Laboratory Animals.

Cell Culture and Transient Transfection

The cell lines used in this study included HepG2, HIBEpic, LO2, BMSC, HeLa, and C3H10. All cells were seeded into 6-well or 24-well plates, and grown in high glucose DMEM (Hyclone, Logan, UT, USA) supplied with 10% (vol/vol) fetal bovine serum (FBS; Gibco BRL, Grand Island, NY, USA) and 1% (vol/vol) penicillin-streptomycin. The following day, cells were transfected with plasmid, mimics, or siRNA. Transient transfection was performed using Lipofectamine 2000 or Lipofectamine RNAIMAX (Invitrogen, Carlsbad, CA). Cells were harvested and the luciferase activity was determined by using a dual-luciferase reporter assay system in accordance with the manufacturer's instructions (Promega, Madison, WI). Luciferase activities were normalized by co-transfection of the control Renilla luciferase plasmid, phRL-TK. The primer sequences were listed in Table S1.

Immunofluorescent Analysis

Liver samples were fixed in 4% paraformaldehyde (PFA) and processed by paraffin embedding and sectioning. Livers were embedded in identical orientation to allow direct comparison of tissue. The fixed tissues were dehydrated and embedded in paraffin. Tissue blocks were cut to 5 μm sections. After extensively washing with PBS, the slides were blocked with PBS-10% goat serum for 90 min. SOX9 antibody (AB5535, Millipore, USA), HOXB6 antibody (sc-166950, Santa Cruz Biotechnology), and hepatocyte nuclear factor (HNF) 4α antibody (Ab41898, Abcam, Cambridge, England) were diluted in PBS, and these antibodies were used to incubate sections at 4℃ overnights. Slides were washed three times with PBS and incubated with corresponding secondary antibodies diluted in PBS for 1 hour, followed by PBS washes. The incubated slides were added with DAPI for nuclear staining and mounting. Images was acquired with a laser scanning confocal microscope (LSM710, Carl Zeiss Microscopy GmbH), were analyzed by Zen software with fixed parameters.

Western Blots

For whole-cell protein extraction, liver tissues were prepared in lysis buffer (Beyotime, China) according to the manufacturer's instructions. Protein lysates were separated by 10% or 12% SDS-PAGE (20 μg each lane). Next, the gel was transferred to polyvinylidene fluoride (PVDF) membranes (Millipore, USA). After being blocked with 5% skimmed milk or 5% BSA in Tris-buffered saline/Tween-20 (TBST), the membranes were incubated overnight with the anti-SOX9 (AB5535, Millipore, USA), anti-HOXB6 (sc-166950X, Santa Cruz Biotechnology), anti-GAPDH (60004-I-Ig, Proteintech, Chicago, USA), or Tag-3*Flag-antibody (66008-3-Ig, Proteintech, Chicago, USA) at 4℃. And then, the membranes were incubated with the corresponding HRP-conjugated secondary antibodies at room temperature for 1.5 hours. Finally, the membranes were visualized with enhanced chemiluminescence (ECL) (Bio-Rad, USA).

Lentivirus, Adenovirus, and Adeno-Associated Virus (AAV) Plasmid Construction and Production

The lentivirus plasmids and adenovirus plasmids were constructed and produced, as described previously. We initially screened candidate CRISPR gRNAs targeting sequences in sgRNA1 and -3 in mice upstream of the pre-miR-126a gene in NIH 3T3 cells. One of them formed a pair of sgRNAs with sgRNA2. The guide RNA targeting sgRNA3 displayed ~50% mutagenesis at the on-target site in *pre-miR-126a*, as determined by PCR and a T7 endonuclease 1 (T7EN1) cleavage assay. To completely overexpress HOXB6 in vivo, the pHBAAV-CMV-HOXB6-3flag-T2A-ZsGreen plasmid was constructed based on pHBAAV-CMV-MCS-3flag-T2A-ZsGreen (Hanbio, Shanghai, China) in which we inserted *Hoxb6*-CDS sequence into the multiple cloning site (CMV). The constructed plasmids were named pAAV-*Hoxb6* and

pAAV-ctrl. The two recombinant AAV plasmids were transfected into HEK293 cells with pAAV-RC and pHelper by using Lipofectamine 2000 transfection reagent (Invitrogen, Carlsbad, CA). AAV-*Hoxb6* and AAV-ctrl (Hanbio, Shanghai, China) were packaged in AAV-293 cells. The recombinant AAV in the AAV-293 cells were purified, and the titer of virus was measured by qRT-PCR. The titer range of stock solutions of AAV-*Hoxb6* and AAV-ctrl were 1.2~1.6X10^{12} vg/mL.

Isolation of Periportal (PP) and Perivenous (PV) Hepatocytes

Hepatocytes were obtained from the fed adult C57BL/6J male mice. Our experiments followed the procedures described previously with the usual digitonin collagenase perfusion technique. Intact mouse liver cells from the PV region were isolated by collagenase perfusion after first destroying the PP region by a brief portal infusion of digitonin. PP cells were isolated after retrograde digitonin infusion, as described previously (Berry and Friend, 1969; Gautam et al., 1987; Lindros and Penttila, 1985; Quistorff, 1985; Tordjmann et al., 1996).

Plasmid Construction

The 3'UTR of *Hoxb6*, which contained the predicted target site for *miR-126-5p*, was cloned into the psicheck-2 luciferase reporter vector which was cleaved at Not I and Xho I sites. The 3'UTR regions containing the potential binding sites and mutant sites of *Hoxb6* were cloned into the luciferase reporter system. The constructed vectors were designated WT and Mut. According to the published *Hoxb6* gene CDS sequence from GenBank, specific primers containing restriction enzyme sites were designed and amplified by PCR. After being digested with restriction endonucleases Kpn I and BamH I, the fragments were ligated into pcDNA3.1(+) to construct the HOXB6 overexpression vector. At ~3kb immediately upstream of the *SOX9* transcriptional start site, we constructed a series of truncated fragments into the vectors, then defined them as *SOX9*-Pro (~1.7kb, the region from -2242 to -535), *SOX9*-P1 (-2242 to -1888), *SOX9*-P2 (-1970 to -1669), *SOX9*-P3 (-1694 to 1407), *SOX9*-P4 (-1441 to 1126), *SOX9*-P5 (-1190 to -903), *SOX9*-P6 (-953 to -664), and *SOX9*-P7 (-771 to -535), *SOX9*-P6-1 (-953 to -832), *SOX9*-P6-2 (-873 to -748), *SOX9*-P6-3 (-790 to -664), *SOX9*-P6-3-1 (-790 to 737), *SOX9*-P6-3-2 (-749 to 704), and *SOX9*-P6-3-3 (-721 to -664). The primer sequences were listed in Supporting Table S4.

Supplemental References

Berry, M.N., and Friend, D.S. (1969). High-yield preparation of isolated rat liver parenchymal cells: a biochemical and fine structural study. The Journal of cell biology *43*, 506-520.

Gautam, A., Ng, O.C., and Boyer, J.L. (1987). Isolated rat hepatocyte couplets in short-term culture: structural characteristics and plasma membrane reorganization. Hepatology *7*, 216-223.

Lindros, K.O., and Penttila, K.E. (1985). Digitonin-collagenase perfusion for efficient separation of periportal or perivenous hepatocytes. The Biochemical journal *228*, 757-760.

Quistorff, B. (1985). Gluconeogenesis in periportal and perivenous hepatocytes of rat liver, isolated by a new high-yield digitonin/collagenase perfusion technique. The Biochemical journal *229*, 221-226.

Tordjmann, T., Berthon, B., Combettes, L., and Claret, M. (1996). The location of hepatocytes in the rat liver acinus determines their sensitivity to calcium-mobilizing hormones. Gastroenterology *111*, 1343-1352.
